# Exploring the biogeography, morphology, and phylogeny of the condylostomatid ciliates (Alveolata, Ciliophora, Heterotrichea), with establishment of four new *Condylostoma* species and a revision including redescriptions of five species found in China

**DOI:** 10.1007/s42995-024-00223-3

**Published:** 2024-06-26

**Authors:** Yong Chi, Fan Wei, Danxu Tang, Changjun Mu, Honggang Ma, Zhe Wang, Khaled A. S. Al-Rasheid, Hunter N. Hines, Xiangrui Chen

**Affiliations:** 1 School of Marine Sciences, Ningbo University, Ningbo, 315211 China; 2 Institute of Evolution & Marine Biodiversity, and Key Laboratory of Evolution & Marine Biodiversity (Education Ministry), Ocean University of China, Qingdao, 266003 China; 3 Marine College, Shandong University, Weihai, 264209 China; 4 Weishan Special Aquaculture Base, Jining, 277600 China; 5 Zoology Department, College of Sciences, King Saud University, Riyadh, 11451 Saudi Arabia; 6 Harbor Branch Oceanographic Institute, Florida Atlantic University, Florida, 34946 USA

**Keywords:** *Condylostoma*, *Condylostomides*, New species, Phylogeny, Revision, Taxonomy

## Abstract

Species of the ciliate class Heterotrichea Stein, 1859 are a cosmopolitan group of unicellular eukaryotic microorganisms, many of which have been widely used as models in various fields of research such as regenerative biology, functional ecology, environmental toxicology, and symbiotic behavior. However, species identification in the heterotrich family Condylostomatidae, especially the most species-rich and type genus *Condylostoma* Bory de Saint-Vincent, 1824, remains challenging due to incomplete original descriptions, few reliable distinguishing characters, and overlapping features between different species. This study presents an updated revision of *Condylostoma* and its related genus *Condylostomides* da Silva Neto, 1994 based on descriptions of five species, including nine populations collected from China, using both morphological and molecular methods. The main findings are as follows: (1) 43 nominal species and about 130 populations are reviewed, resulting in the recognition of 30 valid species of *Condylostoma* and eight valid species of *Condylostomides*; (2) keys, synonyms, biogeographic distributions and amended/improved diagnoses of all valid species are provided; (3) based on the available data, four new *Condylostoma* species (*C. marinum* sp. nov., *C. petzi* sp. nov., *C. villeneuvei* sp. nov., and *C. microstomum* sp. nov.), one new combination (*Condylostomides minimus* (Dragesco, 1954) comb. nov. & nom. corr.), and two corrected names (*Condylostoma ancestrale* Villeneuve-Brachon, 1940 nom. corr. and *Condylostomides nigrus* (Dragesco, 1960) nom. corr.) are suggested; (4) cryptic species are detected and proposed for the first time to form the *Condylostoma curvum* species complex; (5) three highly confusing *Condylostoma* species, *C. kris*, *C. spatiosum*, and *C. minutum*, are redefined for the first time based on modern taxonomic methods; (6) a ‘flagship’ species, *Condylostomides coeruleus*, is recorded for the first time from the continent of Asia, substantially expanding its biogeography; (7) ciliature adjacent to the distal end of the paroral membrane within the family Condylostomatidae is uniformly defined as frontal membranelles and is classified into three patterns according to the arrangement of kinetosomes, which serve as important key features.

**Supplementary Information:**

The online version contains supplementary material available at 10.1007/s42995-024-00223-3.

## Introduction

Ciliated protists, a diverse group of single-celled organisms, are vital components of various aquatic and terrestrial environments where they are important contributors to nutrient cycling and energy transfer (Hu et al. 2019; Lu et al. 2023; Lynn 2008; Ma et al. 2022a; Wang et al. 2022; Ye et al. 2021a; Zhang et al. 2022a). Members of the class Heterotrichea Stein 1859, a primitive group of ciliates characterized by their large cell size, somatic dikinetids associated with postciliodesmata, and a well-developed oral apparatus composed of an adoral zone of membranelles and a paroral membrane, have been widely used as model organisms in numerous biological disciplines including regeneration, ecology, nuclear function, and symbiotic relationships (Ahsan et al. 2022; Chi et al. 2021; Fernandes et al. 2016; Hao et al. 2022; Shazib et al. 2014; Yan et al. 2019; Ye et al. 2022).

The family Condylostomatidae is a relatively well-studied family within the Heterotrichea, and several studies on the diversity of its five genera, *Chattonidium* Villeneuve-Brachon, 1937, *Condylostentor* Jankowski, 1978, *Condylostoma* Bory de Saint-Vincent, 1824, *Condylostomides* da Silva Neto, 1994, and *Linostomella* Aescht in Foissner et al., 1999, have been reported in recent years (Chen et al. 2007; Chi et al. 2020b; Fernandes et al. 2015; Jin et al. 2021; Kim et al. 2012; Modeo et al. 2006; Shao et al. 2006; Yan et al. 2015). The type genus, *Condylostoma*, is the most speciose of these genera and is commonly found in brackish or marine habitats. It is easily recognized by its large cell size and prominent buccal region (Chen et al. 2007; Fernandes et al. 2015; Kim et al. 2012; Shao et al. 2006; Yan et al. 2015). Although the genus *Condylostoma* has a research history spanning over two centuries, with species recognizable in texts from as early as the 1780’s (Müller 1786), it remains one of the most confusing taxa among ciliates in terms of species identification and separation. Song et al. (2003) summarized the reasons for this as follows: (1) many species exhibit variable morphological characters (e.g., cell shape, size, pattern of ciliature); 2) only a few characters can be reliably used for species separation; (3) the morphology of most species has not been fully described; (4) many species have overlapping key features such as the number of somatic kineties, cell shape, and cell size; and (5) a mass of misinterpretations or inadequate descriptions have accumulated in the literature as a result of these problems. So far, only four *Condylostoma* species, namely *C. arenarium*, *C. curvum*, *C. elongatum*, and *C. tropicum*, have been defined using an integrative taxonomic approach that includes living morphology, stained preparations, and molecular data (Fernandes et al. 2015; Yan et al. 2015). Nevertheless, with the rapid development of molecular methodologies in ciliate research, large amounts of molecular data on *Condylostoma* have been submitted to the GenBank database in recent years (Chen et al. 2019; Gao et al. 2016; Guo et al. 2008; Shazib et al. 2014; Zhou et al. 2010). However, due to the lack of reliable data for taxonomic identification, many of these sequences are likely assigned to the wrong species, which further exacerbates the understanding of the systematics of *Condylostoma*. The last revision to the genus *Condylostoma*, over 90 years ago, predates modern methods of morphological investigation including several commonly used fixatives and histological staining techniques and, of course, the molecular era, and was based only on 10 nominal species (Kahl 1932). Therefore, a comprehensive revision of *Condylostoma* is needed to better understand the evolutionary relationships, morphology, and biogeography of species within this group.

Unlike *Condylostoma*, which mainly comprise marine species, the genus *Condylostomides* da Silva Neto, 1994 is generally encountered in freshwater or terrestrial soil habitats (da Silva Neto 1994; Foissner 2016; Foissner et al. 2002; Hines et al. 2020). The overall morphology of these two genera is very similar and is characterized by a prominent buccal area, which has been a contributing factor to potential misidentifications (Foissner 2016; Foissner et al. 2002; Kahl 1932; Penard 1922). Interestingly, *Condylostomides coeruleus* was perceived as an endemically distributed species in the original report based on soil samples from Venezuela (Foissner 2016). Subsequently, however, Hines et al. (2020) discovered this ‘flagship’ ciliate species on the North American continent, indicating that additional sampling is needed to fully reveal its true geographical distribution.

Here we conducted a comprehensive reassessment of 43 nominal species and about 130 populations belonging to *Condylostoma* and *Condylostomides*. Ultimately, we recognized 30 valid *Condylostoma* species and eight valid *Condylostomides* species, and provided the synonyms, diagnoses, and biogeography for each. Drawing on the present review and the redescriptions of four highly confusable *Condylostoma* species (*C. kris*, *C. curvum*, *C. spatiosum*, and *C. minutum*) and one *Condylostomides* species (*C. coeruleus*), we proceeded to refine the diagnostic features of both genera. Finally, based on current phylogenetic analyses inferred from SSU rDNA sequences, we re-evaluated the relationships between species within the family Condylostomatidae and revealed for the first time the presence of cryptic species in *Condylostoma curvum*.

## Material and methods

### Sample collection and observation

Five species, i.e., *Condylostoma kris* (two populations), *C. curvum* (three populations), *C. spatiosum*, *C. minutum* (two populations), and *Condylostomides coeruleus*, were collected from Qingdao, Ningbo, and Lake Weishan Wetland in China. The collection sites and dates are shown in Table 1, and the geographical information and physicochemical parameters are shown in Fig. 4B–J. All samples were taken from the surface layer of sediment using a pipette and transferred into Petri dishes for processing in the laboratory.

**Table 1:** Sampling information and molecular data of nine populations from this study

Species	Collection data		SSU rDNA data		
	Site	Date	Length	GC (%)	GB number
*Condylostoma kris* pop.1*	Coastal area off Qingdao, China	16th Jan, 2019	1650	46.61	MT175508
*Condylostoma kris* pop.2	A brackish lake, Ningbo, China	15th Jul, 2019	1558	47.37	OR553808
*Condylostoma curvum* pop.1	A brackish lake, Ningbo, China	4th Dec, 2019	1560	48.01	OR553809
*Condylostoma curvum* pop.2	Estuary area, Ningbo, China	28th Nov, 2019	1552	47.74	OR553810
*Condylostoma curvum* pop.3	An artificial river, Ningbo, China	1st May, 2020	1551	47.90	OR553811
*Condylostoma spatiosum*	Coastal area off Qingdao, China	13th Apr, 2019	1619	46.39	OR553812
*Condylostoma minutum* pop.1	An open beach, Ningbo, China	11th Jul, 2019	1567	46.78	OR553813
*Condylostoma minutum* pop.2	A brackish lake, Ningbo, China	15th Nov, 2018	1563	46.77	OR553814
*Condylostomides coeruleus*	A downstream river flowing through the Lake Weishan Wetland, China	15th Jun, 2020	1657	45.81	OR553815

^*^Molecular data reported in Chi et al. (2021)

Living cells were haphazardly selected from the original samples and observed at 100–1000× magnification using both bright field and differential interference contrast microscopy (Olympus BX53; Zeiss AXIO Imager. D2) and photographed with Olympus DP74 and Axiocam 506 color cameras. The protargol staining method (Wilbert 1975) was used to determine the ciliary pattern. The protargol powder was synthesized according to Pan et al. (2013). Hoechst 33,342 solution was used to reveal the nuclear apparatus (Jiang et al. 2019). Counts and measurements of stained specimens were conducted at 100× and 1000× magnifications. Drawings of living and stained cells were accomplished with the help of Adobe Photoshop. Terminology and systematics are mainly according to Chi et al. (2021), Lynn (2008), and Shazib et al. (2014).

### DNA extraction and gene sequencing

One cell of each population was isolated under a dissecting microscope and washed five times in filtered habitat water to remove potential contaminants. Genomic DNA was extracted from the single cleaned cell using a DNeasy Blood & Tissue Kit (QIAGEN, Hilden, Germany) following the manufacturer’s instructions but modified by using 1/4 of the suggested volume for each solution (Chi et al. 2022a). SSU rDNA was amplified using Q5 Hot Start high-fidelity DNA polymerase (NEB, Ipswich, MA, USA) with the universal eukaryotic primers, 18 s-F (5’- AACCTGGTTGATCCTGCCAGT-3’) and 18 s-R (5’-TGATCCTTCTGCAGGTTCACCTAC-3’) (Medlin et al. 1988; Ye et al. 2021b). PCR amplifications were according to the following procedure: 1 cycle of initial denaturation at 98 ℃ for 30 s, followed by 18 cycles of amplification (98 ℃, 10 s; 69–51 ℃ touchdown, 30 s; 72 ℃, 1 min), and another 18 cycles (98 ℃, 10 s; 51 ℃, 30 s; 72 ℃, 1 min), with a final extension of 72 ℃ for 5 min (Ma et al. 2022b; Zhang et al. 2022b). PCR products were checked using agarose gel and then sequenced bidirectionally in TSINGKE (Qingdao, China). Sequence fragments were assembled into contigs using Seqman (DNAStar).

### Molecular analyses

Apart from the eight new sequences produced during this study, all sequence data used in the phylogenetic inference analyses were obtained from the GenBank database (accession numbers are provided in Fig. 5A). Five karyorelictean species were used as the outgroup. Sequence alignments were performed using MAFFT with the L-INS-I strategy (Katoh and Standley 2013). Ambiguously aligned regions were manually edited using BioEdit (Hall 1999). The final alignment, including 1586 sites, was used to construct phylogenetic trees.

A maximum likelihood (ML) tree was constructed by IQ-TREE v2.0 with 10,000 ultrafast bootstrap, using SYM + I + G4 as the best-fit model (Minh et al. 2020). Bayesian inference (BI) analysis was carried out using MrBayes version 3.2.7a on XSEDE (Ronquist et al. 2012) on the CIPRES Science Gateway (Miller et al. 2010). GTR + I + G was selected as the best model by MrModeltest version 2.2 according to the Akaike Information Criterion (Nylander 2004). Markov chain Monte Carlo (MCMC) simulations were run for ten million generations at a sample frequency of 100 generations, with the first 25% discarded as burn-in. Convergence of the Bayesian analysis was determined by the average standard deviation of split frequencies (< 0.01) (Ronquist et al. 2011). MEGA 5.2 (Tamura et al. 2011) was used to visualize the tree topology. Nucleotide differences were analyzed by BioEdit (Hall 1999).

## Results and discussion

### ZooBank registration of present work

urn:lsid:zoobank.org:pub:E73DACE1-B1C4-4EB4-A0B8-22A704B2973E.

### Genus *Condylostoma* Bory de Saint-Vincent, 1824

#### Synonyms

*Kondyliostoma*, *Kondylostoma*, *Codyliostoma* (See Aescht 2001 for details).

#### Improved diagnosis

Medium-sized (about 100 µm) to very large (up to 2.5 mm) heterotrich ciliates, freely motile, usually crawling on substrates or between sediment particles, occasionally swimming. Cell moderately contractile; when swimming or crawling slender ribbon-shaped, elongate-ellipsoidal or fusiform; when fixed and contracted, usually ellipsoidal or ovoidal. Somatic ciliature holotrichous, somatic cilia arranged in evenly spaced longitudinal rows, several shortened kineties form a suture on ventral side near posterior end of cell. Buccal area prominent with conspicuous cavity, adoral zone of membranelles at left margin of peristome, proximal region spiraling down to cytostome. Prominent paroral membrane on the opposite side of buccal cavity. Frontal membranelles at distal end of paroral membrane. Macronucleus usually moniliform, rarely nodular, segmented or vermiform. Contractile vacuole absent. Cortical granules colorless or lightly colored, e.g., brownish, yellowish, or greenish. Only one species with symbiotic green algae. Inhabiting mainly marine or brackish water, rarely freshwater biotopes.

#### Type species

*Condylostoma patens* (Müller, 1786) Dujardin, 1841.

#### Etymology

The generic name is a composite of the Greek words “condylo = kondylo” (knuckle, knuckle-like knob) and “stoma” (mouth), meaning a ciliate with distinct mouth. Neuter gender.

#### Identification characteristics

Recent studies of *Condylostoma* species (Chen et al. 2007; Fernandes et al. 2015; Kim et al. 2012; Shao et al. 2006; Song et al. 2003; Yan et al. 2015), including the present investigation, have allowed for a more precise understanding of the taxonomic significance of various features. Among the main living characters, the cell shape, nuclear apparatus, and cortical granules play crucial roles in distinguishing among different species. Additionally, specific characteristics that are only recognizable in silver-stained specimens, such as the number of somatic kineties and the pattern of frontal membranelles, are also important for accurate species identification.

**Fig. 1:**
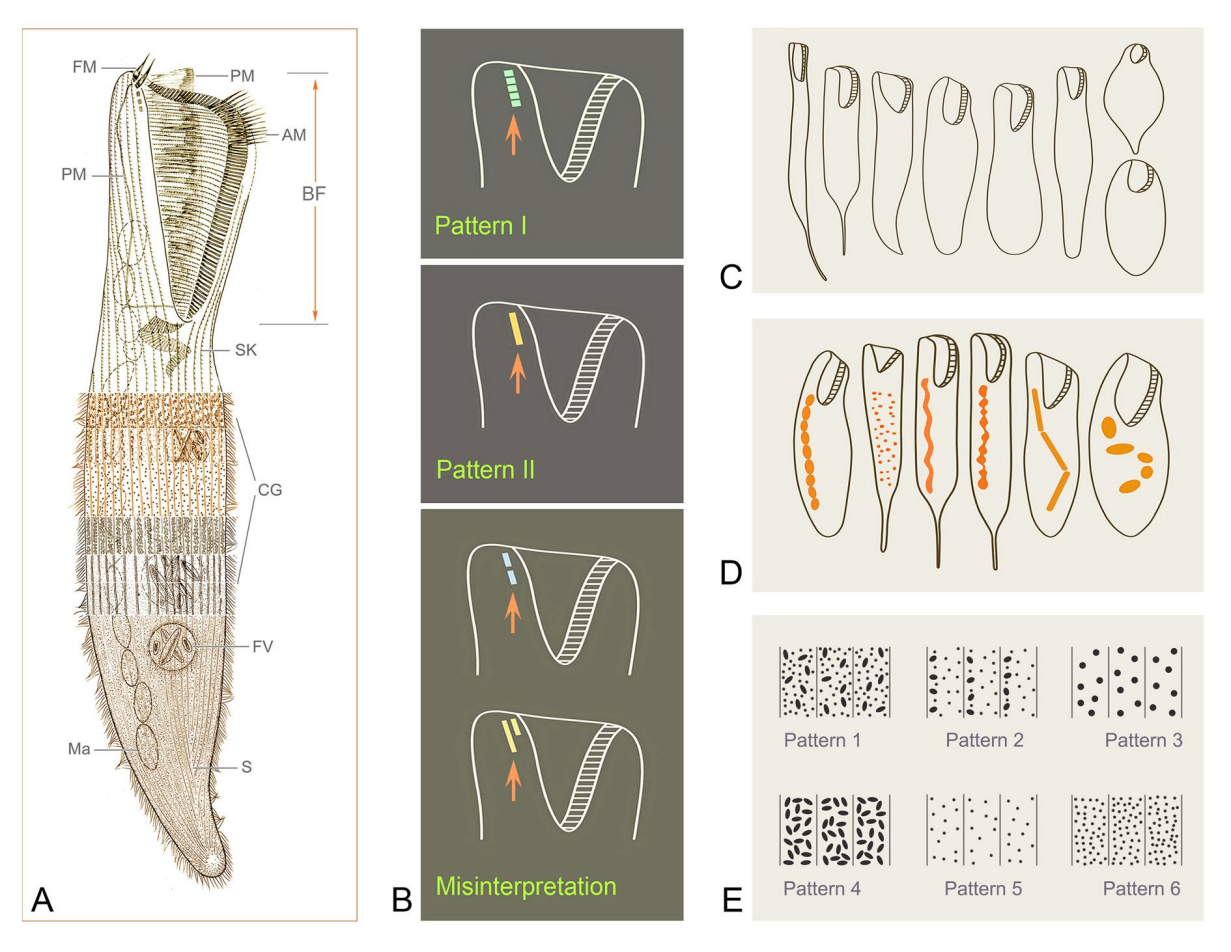
Introduction to morphological characters of *Condylostoma*. **A** A typical idealized individual to show the key morphological structures. **B** Kinetosome arrangement patterns of frontal membranelles (arrows). **C** Outlines of different cell shapes. **D** Various macronuclear shapes in orange. **E** Various patterns of cortical granules. *AM* adoral membranelles, *BF* buccal field, *CG* cortical granules, *FM* frontal membranelles, *FV* food vacuole, *Ma* macronucleus, *PM* paroral membrane, *S* suture, *SK* somatic kineties

***Cell shape:*** The cell shape is highly variable, both between and within species. In fully extended or free-swimming forms, at least four typical cell shapes can be recognized (Fig. 1C): (1) ribbon-shaped or slender with a sharp posterior end or needle-like tail; (2) flattened slender with a rounded posterior end; (3) flattened ellipsoidal with a rounded posterior end; and (4) elongated cylindrical with a rounded posterior end. Some other peculiarities can also occur, such as in *Condylostoma subterraneum* the cell of which is triangular with a long tail.

***Nuclear apparatus:*** The shape of the macronucleus is a reliable character for identifying different *Condylostoma* species. In the majority of species, the macronucleus is moniliform, and is formed by a chain of beads or nodules connected by thread-like extensions of the nuclear membrane. However, the macronucleus can also take other forms, such as many scattered spherical nodules in *C. fjeldi*, a single ribbon-shaped macronucleus in *C. enigmaticum*, or a multi-nodular macronucleus that can be considered a special ribbon-shaped type in *C. acutum*. In some cases the macronucleus may be composed of only a few isolated, spherical or sausage-like nodules (Fig. 1D).

***Cortical granules:*** Like most other heterotrichs, the cortex of *Condylostoma* has distinct striations due to broad stripes of cortical granules that alternate with clear furrows containing the ciliary rows. Two size classes of granules can be roughly distinguished: small (< 1 μm) and large (> 1 μm) size. These granules are usually spherical or ellipsoidal, but sometimes fusiform or short rod-shaped. They can be colorless or slightly colored, such as gray, green, brown, or red-brown. The size, shape, and density of granules between two adjacent ciliary rows are useful characters to distinguish species. According to the available data, cortical granules can be roughly classified into six patterns (Fig. 1A, E): (1) Pattern 1 with two types: Type I, large, ellipsoidal, loosely arranged; Type II, small, spherical, densely arranged, such as in *C. kris*; (2) Pattern 2 with two types: Type I, large, fusiform, loosely arranged in one row; Type II, small, spherical, loosely arranged, such as in *C. enigmaticum*; (3) Pattern 3 with one type: large, spherical to ellipsoidal, loosely arranged, such as in *C. elongatum*; (4) Pattern 4 with one type: large, ellipsoidal, densely arranged, such as in *C. curvum*; (5) Pattern 5 with one type: small, near-spherical, loosely arranged, such as in *C. pauculum*; (6) Pattern 6 with one type: small, ellipsoidal, densely arranged, such as in *C. arenarium*.

***Cell size:*** The size varies depending on contractility or nutritional status, and thus it is considered a weak character for species identification. Three size classes can be roughly distinguished: small (< 400 μm), medium (400–1000 μm), and large (> 1000 μm).

***Number of somatic kineties and adoral membranelles:*** Some available data suggest that there is slight variability in the number of somatic kineties and adoral membranelles both between and within populations. Specifically, smaller cells tend to have fewer than larger ones. Most species have a relatively stable range of these characters, although there is also some variability in certain species, possibly due to some data being based on living cells and the variable number of shortened somatic kineties in some specimens. Nonetheless, the numbers of somatic kineties and adoral membranelles are generally considered reliable characters for distinguishing *Condylostoma* species, based on the current state of knowledge.

**Fig. 2:**
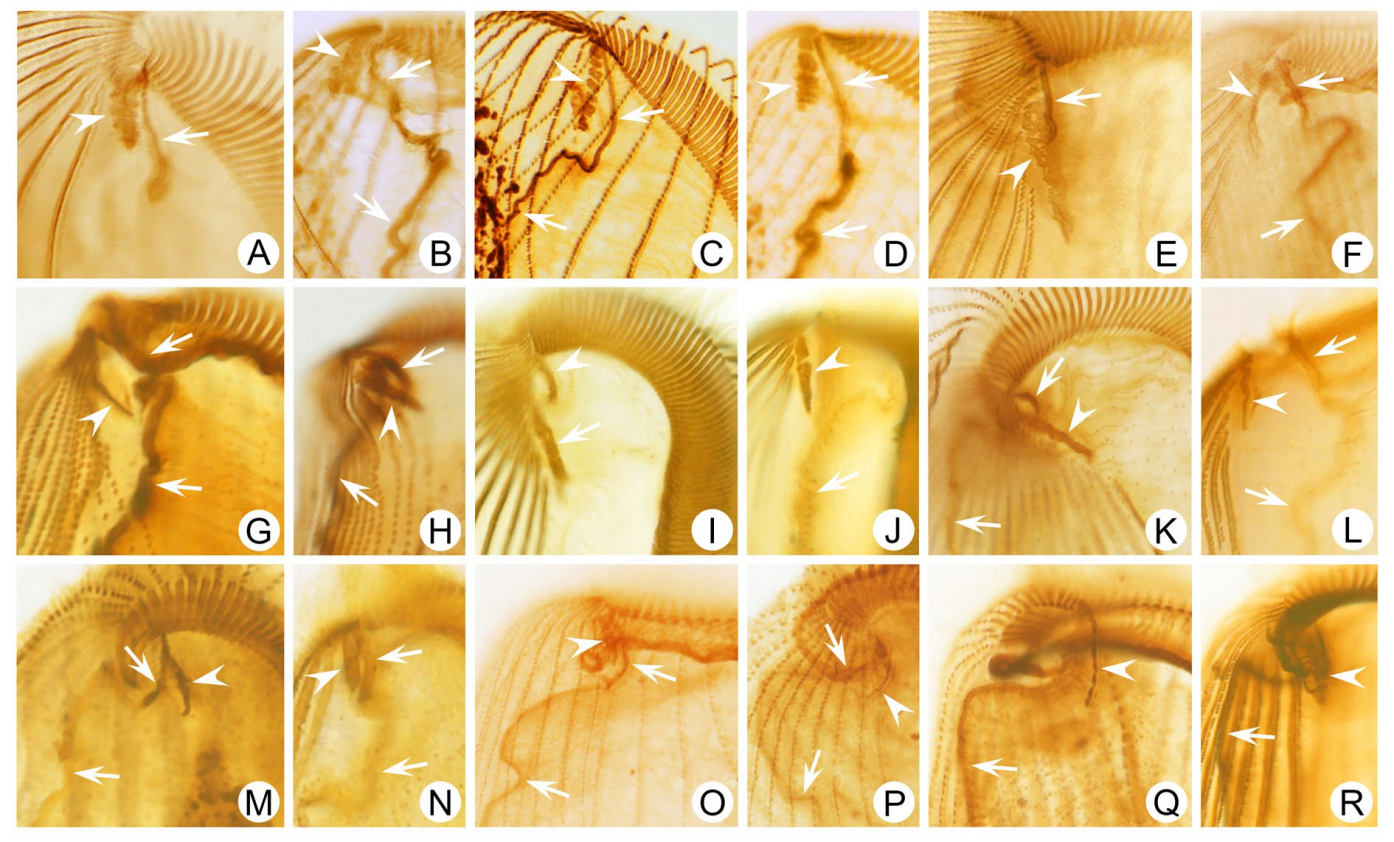
Comparison of frontal membranelles among different *Condylostoma* species. **A**,** B** *C. kris* pop.1 (**A**) and pop.2 (**B**) from this study.** C**,** D** *C. curvum* pop.1 (**C**) and pop.2 (**D**) from this study.** E**,** F** *C. spatiosum* from this study.** G**,** H** *C. minutum* pop.2 from this study. **I**, **J** *C. magnum* reported by Song and Wilbert (1997), as a synonym of *C. spatiosum* in the present study. **K**, **L** *C. spatiosum* reported by Shao et al. (2006). **M**, **N** *C. minutum* reported by Chen et al. (2007). **O**, **P** *C. tropicum* reported by Yan et al. (2015). **Q**, **R** *C. elongatum* reported by Yan et al. (2015). Arrows mark the paroral membrane; arrowheads mark the frontal membranelles

***Frontal membranelles:*** The frontal membranelles are located at the distal end of paroral membrane and have thus far been referred to as “frontal cirri” until the present study. However, the term “frontal cirri” is commonly used in descriptions of hypotrichs and euplotids and is considered a unique characteristic of these groups. Our results indicate that there are two patterns of kinetosome arrangement in this structure in *Condylostoma*, with one being block-like (Pattern I) (Fig. 2A–D) and the other being strip-like (Pattern II) (Fig. 2E–R). The latter pattern is obviously not suitable to be referred to as frontal cirri. Additionally, in the closely related genus *Condylostomides*, which has a similar morphology to *Condylostoma*, this structure is also present in the same position and is referred to as “frontal membranelles”. Hence, to avoid confusion and to maintain consistency, we propose using the term “frontal membranelles” to refer to the ciliary organelle located at the distal end of paroral membrane in both *Condylostoma* and *Condylostomides*. However, it should be noted that the frontal membranelles in *Condylostomides* differ from Patterns I and II described above. In *Condylostomides*, they are closely connected to the paroral membrane and do not seem to represent a completely separate structure. Hence, it can be considered as another unique pattern of frontal membranelles (Pattern III) within the family Condylostomatidae.

The frontal membranelles are a valuable taxonomic feature in *Condylostoma*. Unfortunately, it is not easy to detect in vivo and was usually overlooked until the widespread use of silver staining methods. Four patterns have been reported within the genus *Condylostoma*: Pattern I, Pattern II, and two misinterpreted patterns (Fig. 1B). The pattern of two fragmented frontal membranelles was derived from a possibly extruded specimen of *C. magnum* (Song and Wilbert 1997), which is regarded as a synonym of *C. spatiosum* in the present work (Fig. 2I, J). As for the pattern of two parallel frontal membranelles (Shao et al. 2006), it has been confirmed that the second frontal membranelle, located near the distal end of paroral membrane, is homologous to the paroral membrane (Chen et al. 2007) (Fig. 2K, L). Additionally, the slightly broadened distal end of the paroral membrane was also observed in our species. Therefore, both of the misinterpreted patterns are actually Pattern II.

**Fig. 3:**
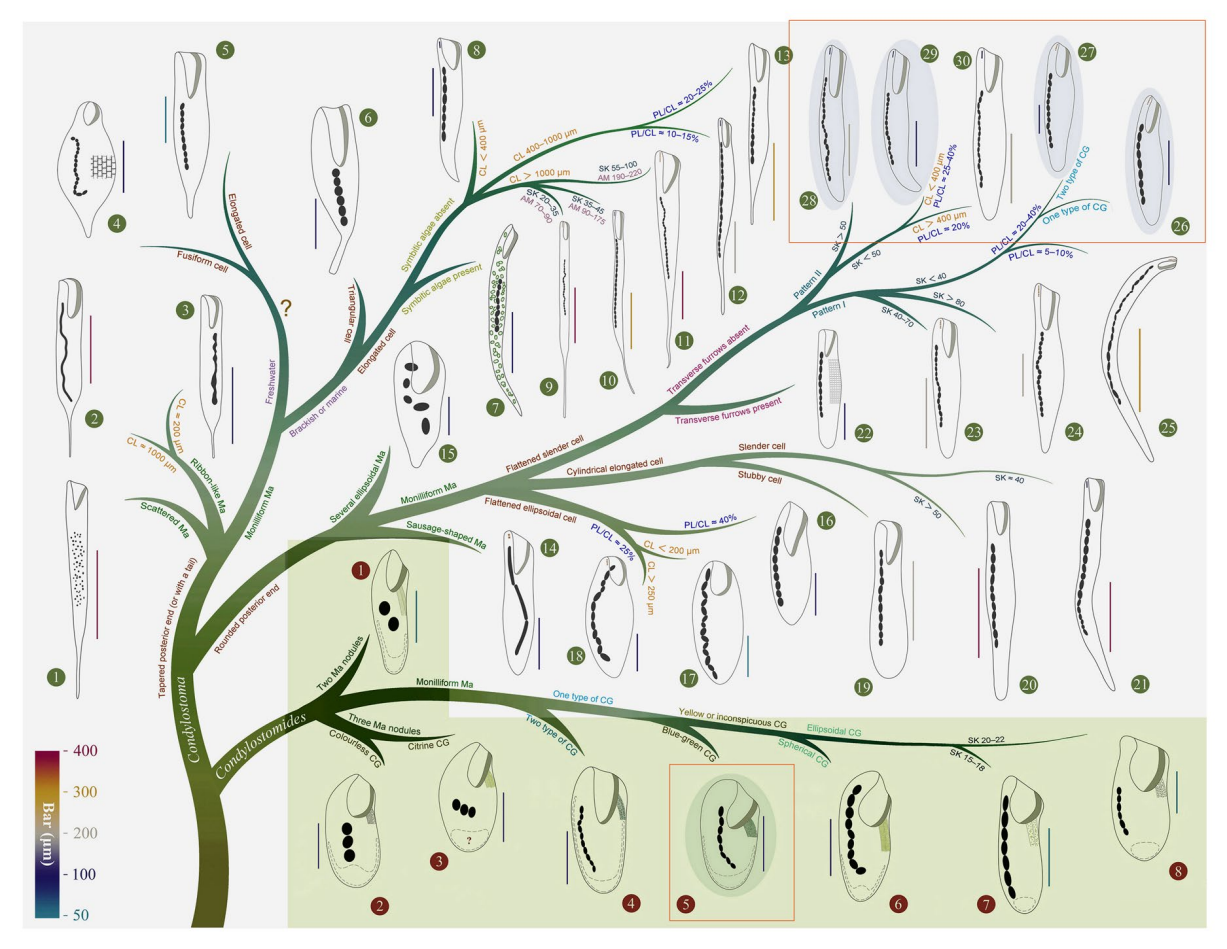
Illustrated key to valid *Condylostoma* and *Condylostomides* species. Green solid circles indicate *Condylostoma* species: 1, *C. fjeldi*; 2, *C. enigmaticum*; 3, *C. acutum*; 4, *C. kasymovi*; 5, *C. caudatum*; 6, *C. subterraneum*; 7, *C. tenue*; 8, *C. pauculum*; 9, *C. longicaudatum*; 10, *C. elongatum*; 11, *C. reichi*; 12, *C. tropicum*; 13, *C. remanei*; 14, *C. psammophium*; 15, *C. kahli*; 16, *C. granulosum*; 17, *C. ancestrale* nom. corr.; 18, *C. vorax*; 19, *C. marinum* sp. nov.; 20, *C. patens*; 21, *C. petzi* sp. nov.; 22, *C. rugosum*; 23, *C. villeneuvei* sp. nov.; 24, *C. magnum*; 25, *C. microstomum* sp. nov.; 26, *C. curvum*; 27, *C. kris*; 28, *C. spatiosum*; 29, *C. minutum*; 30, *C. arenarium*. Red solid circles indicate *Condylostomides* species: 1, *C. luteus*; 2, *C. tardus*; 3, *C. trinucleatus*; 4, *C. nigrus* nom. corr.; 5, *C. coeruleus*; 6, *C. etoschensis*; 7, *C. terricola*; 8, *C. minimus* comb. nov. & nom. corr. Question mark indicates the doubtful validity of two freshwater *Condylostoma* species. *AM* adoral membranelles, *CL* cell length, *CG* cortical granules, *Ma* macronucleus, *PL* peristome length, *SK* somatic kineties

Based on the aforementioned analyses and criteria, we conducted a reassessment of 35 nominal *Condylostoma* species (including approximately 120 populations), utilizing available morphological data such as published illustrations and key diagnostic characters. Through this comprehensive examination, the results are summarized as follow: (1) a total of 30 valid species were recognized, four of which are new (Fig. 3); (2) *Condylostoma patulum* was listed as a synonym of *C. patens*; (3) five nominal *Condylostoma* species, *C. luteum*, *C. minima*, *C. nigra*, *C. tardum*, and *C. terricola*, have been or should be transferred to *Condylostomides* (Foissner 2016; Foissner et al. 2002); (4) three nominal *Condylostoma* species, *C. stagnale*, *C. vastum*, and *C. vorticella*, have been or should be transferred to *Linostomella* (Chi et al. 2020b; Jin et al. 2021).

#### Key to valid *Condylostoma* species

Note: The numbers within parentheses correspond to the order of species in synonym lists, diagnoses, and distributions.

- **1** Gradually tapered at posterior portion or forming a conspicuous tail …………………..………….. **2**
  - **1.1** Rounded at posterior end ……………………………………………..…………………….. **12**
- **2** Moniliform macronucleus …………………………………………………………………………… **4**
  - **2.1** Scattered spherical macronucleus …………………………………….……………(1) *C. fjeldi*
  - **2.2** Ribbon-like macronucleus ……………………………………………..…………………….. **3**
- **3** Cell length almost 1000 μm………………………………………….….…………(2)* C. enigmaticum*
  - **3.1** Cell length about 200 μm…………………………………..….…………………(3) *C. acutum*
- **4** Brackish or marine habitats …………………………………………………………………………. **6**
  - **4.1** Freshwater habitat ……………………………………………………………………………. **5**
- **5** Fusiform cell shape ……………………………………………………………………………….(4)* C. kasymovi*
  - **5.1** Elongated cell shape ………….………..……………………………………..(5) *C. caudatum*
- **6** Elongated cell shape ………………………………………………………………………….……… **7**
  - **6.1** Triangular cell shape …………………………………………..………….(6) *C. subterraneum*
- **7** Symbiotic algae absent ……………………………………………………………………………… **8**
  - **7.1** Symbiotic algae present …………………………………………….……….……..(7) *C. tenue*
- **8** Cell size large, more than 1000 μm …………………………………………….……………………. **9**
  - **8.1** Cell size medium, about 400–1000 μm …………………………………….…………….… **11**
  - **8.2** Cell size small, less than 400 μm ……………………………………….…….(8) *C. pauculum*
- **9** Peristome length about 20% of cell length ……………………………………….……………….. **10**
  - **9.1** Peristome length about 10% of cell length ……………….………..……(9) *C. longicaudatum*
- **10** Somatic kineties 35–45, adoral membranelles 90–175 …………………..……….(10) *C. elongatum*
  - **10.1** Somatic kineties 55–100, adoral membranelles 190–220 ……….…….………..(11)* C. reichi*
- **11** Peristome length about 10–15% of cell length ………………………….…….…….(12) *C. tropicum*
  - **11.1** Peristome length about 20–25% of cell length ……………………………….(13)* C. remanei*
- **12** Moniliform macronucleus ………………………………………………………………………… **13**
  - **12.1** Sausage-shaped macronucleus with three fragments ………………….(14) *C. psammophium*
  - **12.2** Several nearly ellipsoidal macronuclei ………………………………………….(15) *C. kahli*
- **13** Flattened ellipsoidal cell shape, the ratio of cell length to width less than 3 …..……………………. **14**
  - **13.1** Cylindrical elongated cell shape, the ratio of cell length to width greater than 3 ……………. **16**
  - **13.2** Flattened slender cell shape, the ratio of cell length to width greater than 3 ………………….. **18**
- **14** Peristome length about 25% of cell length …………………………………………………..…… **15**
  - **14.1** Peristome length about 40% of cell length ………………..………….…..(16) *C. granulosum*
- **15** Cell size less than 200 μm ……………………………………………………..(17) *C. ancestrale* nom. corr.
  - **15.1** Cell size more than 250 μm …………………….……………..…….…………....(18) *C. vorax*
- **16** Cell slender, widest in the middle ……………………………..………….…….…………………. **17**
  - **16.1** Cell stubby, widest at the rear portion ………………………..….…(19) *C. marinum* sp. nov.
- **17** Somatic kineties more than 50 ………………………………………….……………..(20)* C. patens*
  - **17.1** Somatic kineties about 40 ……………………………………….………………..(21) *C. petzi* sp. nov.
- **18** Transverse furrows absent on the cortex ………………………………………………………….. **19**
  - **18.1** Transverse furrows present on the cortex ……………………………………(22) *C. rugosum*
- **19** Frontal membranelles with Pattern I arrangement …………………………..…….……………… **20**
  - **19.1** Frontal membranelles with Pattern II arrangement ………………..………………………….… **23**
- **20** Somatic kineties usually less than 40 …………………………………………………………..… **21**
  - **20.1** Somatic kineties usually 40–70 ………………………….……….(23) *C. villeneuvei* sp. nov.
  - **20.2** Somatic kineties greater than 80 ……………………………….……………(24) *C. magnum*
- **21** Peristome large, about 1/5–2/5 of cell length ……………….……………..………………….…. **22**
  - **21.1** Peristome small, about 1/15–1/10 of cell length ………….…..(25) *C. microstomum* sp. nov.
- **22** Size usually less than 400 μm, one type of cortical granules ………………………..(26) *C. curvum*
  - **22.1** Size usually more than 400 μm, two types of cortical granules ….………………(27) *C. kris*
- **23** Somatic kineties less than 50 ………………………………………………………….………….. **24**
  - **23.1** Somatic kineties greater than 50 ………………………………….………..(28) *C. spatiosum*
- **24** Size less than 400 μm, peristome about 1/4–2/5 of cell length ………….…………(29) *C. minutum*
  - **24.1** Size more than 400 μm, peristome about 1/5 of cell length …….….……..(30) *C. arenarium*

#### Synonym lists, diagnoses, and geographical distributions of valid *Condylostoma* species

Note: In the list of synonyms for each species, we have directly cited the unaltered species names (as well as author names) from the original sources. This is to demonstrate the transition of species names from historical references to the contemporary era, despite several identifiable errors that are subsequently present throughout.

**1.** ***Condylostoma fjeldi*** **Hartwig, 1973**

[1] 1955 *Condylostoma remanei* Spiegel, 1932 (type B)—Fjeld, Nytt Mag. Zool. 3: 50–57 [Figs. 33, 37, 38] (type B of *C. remanei* as synonym).

[2] 1973 *Condylostoma fjeldi* n. sp.—Hartwig, Abh. Math.-Naturwiss. Kl., Akad. Wiss. Lit., Mainz 18: 46, 47 [Figs. 12, 13] (establishment of this species based on a German population).

[3] 1977 *Condylostoma fjeldi* Hartwig, 1973—Hartwig & Parker, J. Mar. Biol. Ass. U.K. 57: 746 (brief redescription without illustration).

Amended diagnosis: Cell size variable, 500–1070 μm; elongated with a long-pointed tail; peristome length occupies 1/7–1/5 of cell length; about 100–200 spherical macronuclei scattered throughout the cytoplasm; cortical granules large, ellipsoidal, irregularly arranged in two or three rows between adjacent somatic kineties (Pattern 4); 22–46 somatic kineties; marine habitat.

Distribution: Drøbak, Norway (Fjeld 1955); Sylt Island, Germany (Hartwig 1973); North Yorkshire, UK (Hartwig and Parker 1977).

Comments: Fjeld (1955) described two types of *C. remanei*: type A with a moniliform macronucleus and type B with numerous scattered macronuclei as shown in *C. fjeldi*. We agree with Hartwig (1973) who listed type B of *C. remanei* as a synonym of *C. fjeldi*.

**2.** ***Condylostoma enigmaticum*** **Dragesco, 1954**

[1] 1954 *Condylostoma enigmatica*, n. sp.—Dragesco, Bull. Soc. Zool. Fr. 79: 68 [Fig. 3a] (original description).

[2] 1960 *Condylostoma enigmatica* Dragesco—Dragesco, Trav. Stn. Biol. Roscoff (N.S.) 12: 295 [Fig. 156] (more detailed description based on the original population).

[3] 2015 *Condylostoma enigmaticum* Dragesco, 1954 nom. corr.—Yan et al., Eur. J. Protistol. 51: 68 (mandatory change of gender ending to be consistent with neuter genus name, no new population description).

Amended diagnosis: Cell size 900–990 μm; elongated with a long and slender caudal part; peristome length about 1/4 of cell length (from original drawing); macronucleus ribbon-like; two types of cortical granules: Type I, large, fusiform, loosely arranged in a single row, Type II, small, loosely arranged (Pattern 2); about 35 somatic kineties (from original drawing); marine habitat.

Distribution: Roscoff, France (Dragesco 1954).

**3.** ***Condylostoma acutum*** **Dragesco, 1960**

[1] 1960 *Condylostoma acuta* n. sp.—Dragesco, Trav. Stn. Biol. Roscoff (N.S.) 12: 297 [Fig. 153] (original description).

[2] 1999 *Condylostoma acuta* Dragesco 1960—Al-Rasheid, Arab Gulf J. Scient. Res. 17: 130 [Fig. 10] (brief redescription based on living and protargol-stained specimens).

[3] 2015 *Condylostoma acutum* Dragesco, 1960 nom. corr.—Yan et al., Eur. J. Protistol. 51: 68 (mandatory change of gender ending to be consistent with neuter genus name, no new population description).

Amended diagnosis: Cell size 170–220 μm; slender with a tapered tail; peristome length about 1/5 of cell length; macronucleus ribbon-like; cortical granules large, spherical to ellipsoidal, loosely arranged (Pattern 3); 14–22 somatic kineties; marine sand habitat.

Distribution: Carantec, France (Dragesco 1960); Jubail, Saudi Arabia (Al-Rasheid 1999).

**4.** ***Condylostoma kasymovi*** **Alekperov, 1984**

[1] 1984 *Condylostoma kasymovi* Alekperov, sp. n.—Alekperov, Zool. Zh. 63: 1733 [Figs. G–I] (original description).

Amended diagnosis: Cell size about 300 μm; fusiform with a pointed posterior end, covered with quadrangular argentophilic meshes; peristome length about 1/6 of cell length (from original drawing); macronucleus moniliform with 15–17 nodules, 3–5 micronuclei; 70–75 adoral membranelles; 65–70 somatic kineties; freshwater habitat.

Distribution: Absheron Peninsula, Azerbaijan (Alekperov 1984).

**5. *Condylostoma*** ***caudatum* Lauterborn, 1908**

[1] 1908 *Condylostoma caudatum* nov. spec.—Lauterborn, Z. Wiss. Zool. 90: 659 [Fig. 20] (original description based on living cells).

[2] 1932 *Condylostoma caudatum* Lauterborn, 1908—Kahl, Tierwelt Dtl. 25: 456 [Figs. S. 454, 10] (short review, no new population description).

Amended diagnosis: Cell size 150–200 μm; elongated, narrowed at the posterior end into a short tail; peristome length about 1/5 of cell length; macronucleus moniliform; freshwater habitat.

Distribution: Upper Rhine and Heidelberg, Germany (Lauterborn 1908).

**6.** ***Condylostoma*** ***subterraneum*** **Lepsi, 1962**

[1] 1962 *Condylostoma subterraneum* n. sp.—Lepsi, Zool. Anz. 168: 465 [Fig. 27] (original description based on living cells).

Amended diagnosis: Cell size 340 μm; posterior region triangular with a conspicuous tail; peristome length about 1/3 of cell length (from original drawing); macronucleus moniliform with six nodules; about 20 somatic kineties, spiraled after fixation; locomotion by swimming; marine habitat.

Distribution: Black Sea shore, Romania (Lepsi 1962).

**7. *Condylostoma tenue*** **Fauré-Fremiet, 1958**

[1] 1958 *Condylostoma tenuis* n. sp.—Fauré-Fremiet, Hydrobiologia 10: 43–48 [Fig. 1] (original description).

**Fig. 4:**
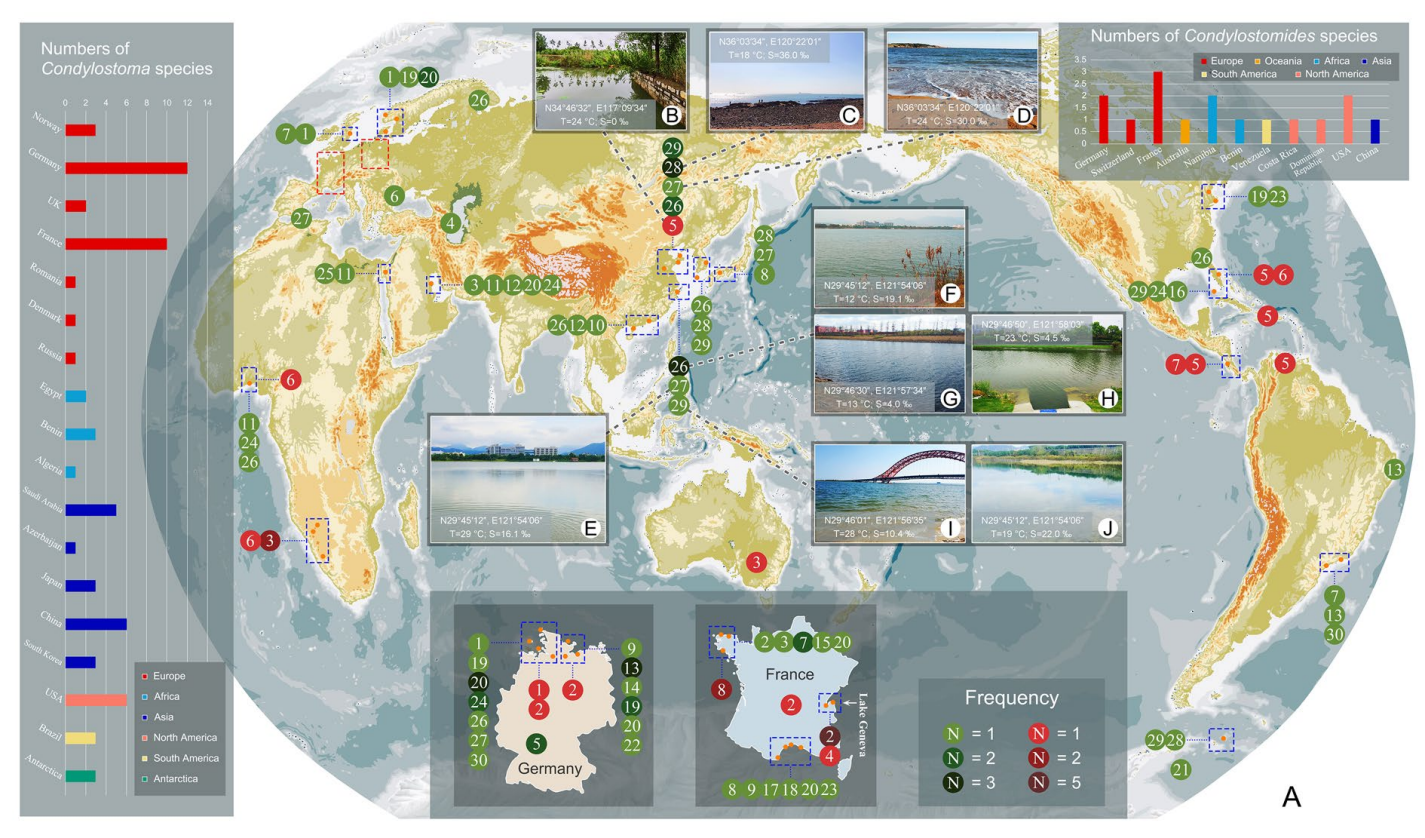
**A** The global geographic distribution of all valid *Condylostoma* and *Condylostomides* species. Green solid circles indicate *Condylostoma* species: 1, *C. fjeldi*; 2, *C. enigmaticum*; 3, *C. acutum*; 4, *C. kasymovi*; 5, *C. caudatum*; 6, *C. subterraneum*; 7, *C. tenue*; 8, *C. pauculum*; 9, *C. longicaudatum*; 10, *C. elongatum*; 11, *C. reichi*; 12, *C. tropicum*; 13, *C. remanei*; 14, *C. psammophium*; 15, *C. kahli*; 16, *C. granulosum*; 17, *C. ancestrale* nom. corr.; 18, *C. vorax*; 19, *C. marinum* sp. nov.; 20, *C. patens*; 21, *C. petzi* sp. nov.; 22, *C. rugosum*; 23, *C. villeneuvei* sp. nov.; 24, *C. magnum*; 25, *C. microstomum* sp. nov.; 26, *C. curvum*; 27, *C. kris*; 28, *C. spatiosum*; 29, *C. minutum*; 30, *C. arenarium*. Red solid circles indicate *Condylostomides* species: 1, *C. luteus*; 2, *C. tardus*; 3, *C. trinucleatus*; 4, *C. nigrus* nom. corr.; 5, *C. coeruleus*; 6, *C. etoschensis*; 7, *C. terricola*; 8, *C. minimus* comb. nov. & nom. corr. **B**–**J** Sampling sites of populations collected during the present study. **B** Sampling site of *Condylostomides coeruleus*. **C** Sampling site of *C. spatiosum*. **D**, **E** Sampling sites of *C. kris* pop.1 (**D**) and pop.2 (**E**). **F**–**H** Sampling sites of *C. curvum* pop.1 (**F**), pop.2 (**G**), and pop.3 (**H**). **I**,** J** Sampling sites of *C. minutum* pop.1 (**I**) and pop.2 (**J**)

[2] 1963 *Condylostoma tenuis* Fauré-Fremiet, 1958—Dragesco, Cah. Biol. Mar. 4: 253–255 [Fig. 4] (detailed redescription based on living observation and silver staining).

[3] 1970 *Condylostoma tenuis* Fauré-Fremiet, 1958—Kattar, Zool. Biol. Mar. 27: 163 [Fig. 17] (simple redescription based on living cells and protargol-stained specimens).

[4] 1977 *Condylostoma tenuis* Fauré-Fremiet, 1958—Kattar & Parker, J. Mar. Biol. Assoc. U.K. 57: 745 (short redescription without illustration).

[5] 2015 *Condylostoma tenue* Fauré-Fremiet, 1958 nom. corr.—Yan et al., Eur. J. Protistol. 51: 68 (mandatory change of gender ending to be consistent with neuter genus name, no new population description).

Amended diagnosis: Cell size variable, 165–600 μm; elongated and gradually tapered at posterior portion; ratio of peristome length to cell length variable, from 1/12 to 1/4; macronucleus moniliform with 6–11 nodules, at least 2 or 3 micronuclei; cortical granules small, spherical, loosely arranged (Pattern 5); symbiotic algae present; 20–24 somatic kineties; marine habitat.

Distribution: Concarneau, France (Fauré-Fremiet 1958); Roscoff, France (Dragesco 1963); Santos, São Paulo, Brazil (Kattar 1970); North Yorkshire, UK (Hartwig and Parker 1977).

Comments: It is noteworthy that the cell size and the ratio of peristome length to cell length exhibit significant variation among different populations. Additionally, the two populations reported by Dragesco (1963) and Kattar (1970) do not demonstrate an obvious tapered posterior end. Considering that only one species has been found to have symbiotic algae thus far, we suggest that these are populations of *Condylostoma tenue*. However, further research is needed to confirm this.

**8. *Condylostoma pauculum*** **Ozaki & Yagiu in Yagiu, 1944**

[1] 1944 *Condylostoma pauculum* Ozaki & Yagiu, 1942—Yagiu, J. Sci. Hirosima Univ. (Ser. B, Div. 1) 10: 177–182 [Figs. 6, 7, S2, S3] (validated the name proposed at the 17th annual meeting of Japanese zoologists in 1942 based on the detailed description).

[2] 1960 *Condylostoma remanei* var. *oxyoura* n. var.—Dragesco, Trav. Stn. Biol. Roscoff (N.S.) 12: 295 [Fig. 155] (synonym proposed in present work).

Amended diagnosis: Cell size 200–400 μm; elongate-ellipsoidal or nearly cylindrical and narrowed posteriorly into a pointed end; peristome length about 1/5–1/4 of cell length; macronucleus moniliform with 7–10 nodules, micronuclei spherical; cortical granules small, near-spherical, pale yellowish-green, loosely arranged (Pattern 5); 48–52 adoral membranelles; 27–30 somatic kineties; frontal membranelles composed of a single stripe of kinetosomes (Pattern II); brackish sand habitat.

Distribution: Onomichi, Japan (Yagiu 1944); Camargue, France (Dragesco 1960).

Comments: Al-Rasheid (1996) listed *C. remanei* var. *oxyoura* as a synonym of *C. remanei*. However, these two taxa can be clearly distinguished in terms of cell size (ca. 400 μm vs. 500–800 μm), posterior cell shape (narrowed into inverted triangle vs. narrowed into a tail), and habitat (brackish with a salinity of 4‰ vs. marine) (Dragesco 1960; Spiegel 1926). Instead, *C. remanei* var. *oxyoura* closely matches *C. pauculum* in having a pointed posterior end, about 28 somatic kineties, and a brackish habitat (Yagiu 1944). Therefore, we suggest that *C. remanei* var. *oxyoura* is a synonym of *C. pauculum*.

**9. *Condylostoma*** ***longicaudatum*** **Dragesco, 1996**

[1] 1928 *Condylostoma longissimum* Grimm?—Kahl, Arch. Hydrobiol. 19: 196–199 [Fig. 36] (two size types of specimens, the larger one should be listed as a synonym of *C. longicaudatum*; see comments on *C. remanei*).

[2] 1932 *Condylostoma remanei* Spiegel, 1928 (nom. n.)—Kahl, Tierwelt Dtl. 25: 456 [Fig. S. 454, 5] (two size types of specimens, the larger one should be listed as a synonym of *C. longicaudatum*; see comments on *C. remanei*).

[3] 1996 *Condylostoma longicaudata* n. sp.—Dragesco, Cah. Biol. Mar. 37: 276–279 [Figs. 55–76] (detailed description based on living and protargol-stained cells).

[4] 2015 *Condylostoma longicaudatum* Dragesco, 1996 nom. corr.—Yan et al., Eur. J. Protistol. 51: 69 (mandatory change of gender ending to be consistent with neuter genus name, no new population description).

Amended diagnosis: Cell size 1200–1600 μm; elongated and flattened with a long and thin tail; peristome small, about 1/10 (or 1/8 according to the drawing of German population) of cell length; macronucleus moniliform with 14–30 nodules, 4–18 micronuclei; 70–90 adoral membranelles; 20–32 somatic kineties; frontal membranelles composed of 8 or 9 blocks of kinetosomes (Pattern I); brackish or marine habitat.

Distribution: Oldesloe, Germany (Kahl 1928); Sète, France (Dragesco 1996).

**10. *Condylostoma*** ***elongatum*** **Yan et al., 2015**

[1] 2015 *Condylostoma elongatum* spec. nov.—Yan et al., Eur. J. Protistol. 51: 70–72 [Figs. 3, 4] (morphology and phylogeny based on living observations, silver-stained preparations, and molecular data).

Amended diagnosis: Cell size 1000–1200 μm; ribbon-shaped with a long tail; peristome length about 1/5 of cell length; macronucleus moniliform with 12–22 nodules; cortical granules large, spherical, colorless, loosely arranged (Pattern 3); 94–171 adoral membranelles; 37–43 somatic kineties; frontal membranelles composed of a single stripe of kinetosomes (Pattern II); marine habitat.

Distribution: Techeng Island at Zhanjiang, China (Yan et al. 2015).

**11. *Condylostoma reichi*** **Wilbert & Kahan, 1981**

[1] 1981 *Condylostoma reichi* n. spec.—Wilbert & Kahan, Arch. Protistenkd. 124: 81 [Figs. 7, 8] (original description based on living cells and protargol preparations).

[2] 1986 *Condylostoma reichi* Wilbert & Kahan, 1981—Dragesco & Dragesco-Kernéis, Faune Tropicale: p399 [Fig. 112] (redescription based on a Cotonou population).

[3] 1999 *Condylostoma reichi* Wilbert and Kahan 1981—Al-Rasheid, Arab Gulf J. Scient. Res. 17: 134 [Fig. 19] (short description with one illustration of a protargol-stained specimen).

Amended diagnosis: Cell size highly variable from 1000–2500 μm; tadpole-shaped and flattened ventrally with an enlarged anterior region and a progressively pointed posterior region; peristome conspicuously large, 1/5 to 1/4 of cell length; macronucleus moniliform with 15–35 nodules, 30–35 micronuclei; 190–220 adoral membranelles; 55–98 somatic kineties; frontal membranelles composed of about 11 blocks of kinetosomes (Pattern I); brackish to hypersaline habitats.

Distribution: Solar Lake, Egypt (Wilbert and Kahan 1981); Cotonou, Benin (Dragesco and Dragesco-Kernéis 1986); Jubail, Saudi Arabia (Al-Rasheid 1999).

**12. *Condylostoma*** ***tropicum*** **Yan et al., 2015**

[1] 1999 *Condylostoma longicaudata* Dragesco 1996—Al-Rashied, Arab Gulf J. Scient. Res. 17: 133 [Figs. 13–16] (simple description with illustrations of protargol-stained specimens; synonym proposed in present work).

[2] 2010 *Condylostoma* sp. 1—Zhou et al., Acta Zootax. Sin. 35: 519 [Fig. 4] (SSU rDNA sequence provided as an unspecified species without morphological description).

[3] 2015 *Condylostoma tropicum* spec. nov.—Yan et al., Eur. J. Protistol. 51:68–70 [Figs. 1, 2] (the establishment of this species based on the population of Zhou et al. (2010) with a detailed morphological description).

Amended diagnosis: Cell size 400–650 μm; slender with a long tail; peristome length 10–15% of cell length; macronucleus moniliform with 8–22 nodules; cortical granules large, spherical, colorless, loosely arranged (Pattern 3); 76–111 adoral membranelles; 26–36 somatic kineties; frontal membranelles composed of a single stripe of kinetosomes (Pattern II); brackish or marine habitat.

Distribution: Jubail, Saudi Arabia (Al-Rasheid 1999); the estuary of Pearl River in Nanshan District, Guangzhou, China (Zhou et al. 2010).

Comments: Before Yan et al. (2015) established this species based on comprehensive morphological data of a Chinese population, the SSU rDNA sequence of this population had been released for exploring the phylogenetic position of *Condylostoma* (Zhou et al. 2010). In the present work, we noticed that a population of *C. longicaudatata* from Saudi Arabia (Al-Rasheid 1999) differed significantly from the original description (Dragesco 1996), especially in size (400–600 μm vs. 1500 μm), but closely resembled *C. tropicum* in terms of various morphological characters. Therefore, we suggest that the Saudi Arabian population is conspecific with *C. tropicum*.

**13. * Condylostoma remanei*** **Spiegel in Kahl, 1932**

[1] 1926 *Condylostoma caudatum* nov. spec.—Spiegel, Arch. Protistenkd. 55: 187, 188 [Fig. C] (homonym of *C. caudatum* Lauterborn, 1908).

[2] 1928 *Condylostoma longissimum* Grimm?—Kahl, Arch. Hydrobiol. 19: 196–199 [Fig. 36] (two size types of specimens, the smaller of which is identified as *C. remanei*).

[3] 1932 *Condylostoma remanei* Spiegel, 1928 (nom. n.)—Kahl, Tierwelt Dtl. 25: 456 [Fig. S. 454, 4] (two size types of specimens, the smaller of which is identified as *C. remanei*).

[4] 1970 *Condylostoma remanei* Spiegel, 1928—Kattar, Zool. Biol. Mar. 27: 162 [Fig. 16] (simple redescription).

Amended diagnosis: Cell size 500–800 μm; elongated with a tapering posterior end or a tail; peristome length 1/5–1/4 of cell length; macronucleus moniliform with about 12 nodules; 24–32 somatic kineties; brackish or marine habitat.

Distribution: Kieler Förde, Germany (Spiegel 1926); Oldesloe, Germany (Kahl 1928, 1932); São Paulo and Recife, Brazil (Kattar 1970).

Comments: When he established this species, Spiegel (1926) was unaware that the name had been occupied by *Condylostoma caudatum* Lauterborn, 1908. Subsequently, Kahl (1932) renamed it *C. remanei* with the agreement of Dr. Spiegel. *Condylostoma remanei* was the first marine species in this genus reported to have a pointed tail, which led to the identification of many morphologically similar populations as being conspecific with it, making this one of the most confusing species.

There are two size types of individuals described in Kahl (1928, 1932). We agree with Yan et al. (2015) and Dragesco (1996) to identify the larger one as *Condylostoma longicaudatum* and the small one as *C. remanei*. In addition, Kahl (1932) also observed two individuals from Kiel with a length of 350 μm, however, due to the lack of key morphological data and illustrations, we are unable to determine their identity. A Brazilian population reported by Kattar (1970) is similar in appearance to *C. pauculum*, however it has a cell length of more than 500 μm (vs. 400 μm) and lives in marine habitat (vs. brackish with a salinity of 4‰), thus we retain this as a population of *C. remanei* pending further investigations. It should also be noted that Villeneuve-Brachon (1940) reported a population collected from freshwater in France. Considering that most of freshwater populations of *Condylostoma* have been proven to be misidentified (Foissner 2016; Foissner et al. 2002), its identity should be verified by future research. Finally, there are still many reports related to *C. remanei* that are not included in present work, e.g., see Al-Rasheid (1996) and the list in Hartwig and Parker (1977), which cannot be reasonably identified due to the lack of sufficiently detailed descriptions and illustrations as well as unreliable reported data.

**14. *Condylostoma*** ***psammophilum*** **Bock, 1952**

[1] 1952 *Condylostoma psammophilum* n. sp.—Bock, Zool. Anz. 149: 113, 114 [Fig. 15] (original description).

Amended diagnosis: Cell size 270–300 μm; cylindrical with a blunt end; peristome length about 1/4 of cell length; macronucleus sausage-shaped, split into three segments; somatic kineties slightly spiraled; frontal membranelles composed of two blocks of kinetosomes (Pattern I); marine habitat.

Distribution: Stollergrund, Kieler Förde, Germany (Bock 1952).

**15. * Condylostoma kahli*** **Dragesco, 1960**

[1] 1960 *Condylostoma kahli* n. sp.—Dragesco, Trav. Stn. Biol. Roscoff (N.S.) 12: 299, 300 [Fig. 160] (original description based on living cells).

Amended diagnosis: Cell size about 360 μm; inverted pyriform with short spines at posterior end; peristome closed apically, occupying about 2/5 (from original drawing) of cell length; about five nearly ellipsoidal macronuclei scattered throughout the cytoplasm; small cortical granules; marine habitat.

Distribution: Roscoff, France (Dragesco 1960).

**16. * Condylostoma granulosum*** **Bullington, 1940**

[1] 1940 *Condylostoma granulosum* n. sp.—Bullington, Pap. Tortugas Lab. 32: 191–193 [Figs. 7, 8] (original description based on living cells).

Amended diagnosis: Cell size variable from 140 to 590 μm, on average about 290 μm; ellipsoidal and flattened, anterior end truncated, posterior bluntly rounded; peristome prominent, about 2/5 of cell length (from original drawing); macronucleus moniliform with 8 or 9 nodules; cortical granules densely arranged between somatic kineties; about 32 somatic kineties; brackish habitat.

Distribution: Bush Key, Florida, USA (Bullington 1940).

Comments: Petz et al. (1995) reported an Antarctic form as *Condylostoma granulosum*. However, based on its extremely variable size (590–2140 μm) and number of somatic kineties (28–65), we agree with Song et al. (2003) that it was misidentified and may contain several different closely related morphotypes.

**17.** ***Condylostoma ancestrale*** **Villeneuve-Brachon, 1940 nom. corr.**

[1] 1940 *Condylostoma ancestralis* n. sp.—Villeneuve-Brachon, Arch. Zool. Exp. Gén. 82: 56–58 [Figs. XX–XXII] (detailed original description based on live and stained specimens).

[2] 1979 *Procondylostoma ancestralis* Villeneuve-Brachon, 1940—Jankowski, Trudy Zool. Inst., Leningr. 86: 7 (transferred as type species, no description).

[3] 2001 *Procondylostoma ancestrale* nom. corr.—Aescht, Denisia 1: 131 (mandatory change of gender ending to be consistent with neuter genus name, no description).

Amended diagnosis: Cell size small, 130–175 μm; wide and short with bluntly rounded anterior and posterior ends; peristome length about 1/4 of cell length; macronucleus moniliform with 11–15 nodules, 15–28 spherical micronuclei; 30–35 somatic kineties; brackish or marine habitat.

ZooBank registration: urn:lsid:zoobank.org:act:189F153B-D7D8-4633–9639-D805F92612E0.

Distribution: Banyuls-sur-Mer, France (Villeneuve-Brachon 1940).

Comments: According to the original report, *Condylostoma ancestrale* nom. corr. has two parallel elongate rows formed by the coalescing kinetosomes at the base of the peristome. This structure appears simpler than cirri, suggesting that it may represent the primitive species of this genus (Villeneuve-Brachon 1940). Jankowski (1979) established the genus *Procondylostoma* for this species based on the absence of cirri and classified it into the order Hypotrichia.

As we discussed earlier, the distal end of the paroral membrane broadens in some *Condylostoma* species, giving it the appearance of the stripy frontal membranelles. Therefore, it is possible that the author misidentified the distal end of the paroral membrane as the second frontal membranelle. Therefore, we propose that this species should be transferred back to *Condylostoma*, and *Procondylostoma* should be considered invalid.

**18.** ***Condylostoma vorax*** **Villeneuve-Brachon, 1940**

[1] 1940 *Condylostoma vorax* n. sp.—Villeneuve-Brachon, Arch. Zool. Exp. Gén. 82: 66–70 [Figs. XXX–XXXII] (original description based on living and stained specimens).

Amended diagnosis: Cell size 250–400 μm; ellipsoidal and flattened with bluntly rounded anterior and posterior ends; peristome length about 1/4 of cell length (from original drawing); macronucleus moniliform with 12 nodules, 8–12 vesicular micronuclei; 30–34 somatic kineties; frontal membranelles composed of four blocks of kinetosomes (Pattern I); brackish or marine habitat.

Distribution: Near Étang de Thau, France (Villeneuve-Brachon 1940).

**19.** ***Condylostoma marinum*** **sp. nov.**

[1] 1858 *Kondylostoma patens*—Claparède & Lachmann, Mém. Inst. Natn. Genev. 5 (Jahr 1857): 244–246 [Fig. XII, 3] (misidentification with simple description based on living cells).

[2] 1867 *Condylostoma patens* Dujard.—Stein, Engelmann pp. 174–177 [Fig. I, 1–4] (detailed description).

[3] 1902 *Condylostoma patens* Müller.—Calkins, Bull. U. S. Fish Comm. 21 (year 1901): 449 [Fig. 45] (simple description).

[4] 1932 *Condylostoma* (*Trichoda*) *patens* (O. F. Müller, 1786) Dujardin, 1841—Kahl, Tierwelt Dtl. 25: 453 [Fig. S. 454, 1] (short review based on a German population).

[5] 1932 *Condylostoma arenarium* Spiegel, 1926—Kahl, Tierwelt Dtl. 25: 455 [Fig. S. 454, 25] (containing three different morphotypes; the forms about 500 μm in size and with an enlarged posterior end should be classified as *C. marinum* sp. nov.).

Diagnosis: Cell size 200–565 μm; mostly cylindrical, posterior portion expanded into a sac-like shape; peristome length 1/6–1/4 of cell length; macronucleus moniliform with 8–25 nodules (from drawings); more than 40 somatic kineties; marine habitat.

Type material: Information unavailable.

Etymology: “*marinum*” was given by Claparède and Lachmann (1858) who first discovered it. See the Comments section of *Condylostoma patens*.

ZooBank registration: urn:lsid:zoobank.org:act:FC1714B3-E2B9-4AA0-8EF8-4F41AF00A713.

Distribution: Bergen, Norway (Claparède and Lachmann 1858); Wismar, Germany (Stein 1867); Woods Hole, USA (Calkins 1902); Kieler Förde and Sylt, Germany (Kahl 1932).

Comments: See the Comments section of *Condylostoma patens* for details on the establishment of this species. In addition to the inclusion of *C. patens* morphotypes with an enlarged posterior end, we note that there are three different morphotypes of the species named *C. arenarium* with 4 or 5 “frontal cirri” and 20–32 somatic kineties (Kahl 1932): Type 1, 400–700 μm in size, typical *C. arenarium*-like individuals, listed here as a synonym of *C. kris* (see the Comments section of *C. arenarium*); Type 2, 200 μm in size, has been listed as a synonym of *C. curvum* (Song et al. 2003); Type 3, 500 μm in size and with an enlarged posterior end. Kahl (1932) mentioned that the “frontal cirri” of Type 3 are weakly visible compared to the other two types, leading us to exclude this feature in the species diagnosis. Similarly, it is inadvisable to include the number of somatic kineties (20–32) in the species diagnosis due to the inclusion of multiple morphotypes. Therefore, we believe it is reasonable to list the Type 3 as a synonym of *C. marinum* sp. nov.

**20.** ***Condylostoma patens*** **(Müller, 1786) Dujardin, 1841**

[1] 1786 *Trichoda patens*—Müller, N. Mölleri p.181 [Figs. XXVI, 1, 2] (simple original description with illustrations).

[2] 1841 *Kondylostoma patens*—Dujardin, Librairie Encyclopédique de Roret pp 516–518 [Fig. XII, 2] (short review and redescription).

[3] 1858 *Kondylostoma patulum*—Claparède & Lachmann, Mém. Inst. Natn. Genev. 5 (Jahr 1857): 246 [Fig. XII, 4] (simple description based on living cells, synonym proposed in present study).

[4] 1932 *Condylostoma patulum* Clap. & L. 1858—Kahl, Tierwelt Dtl. 25: 453 [Figs. S. 454, 9] (simple description, synonym proposed in present study).

**Fig. 5:**
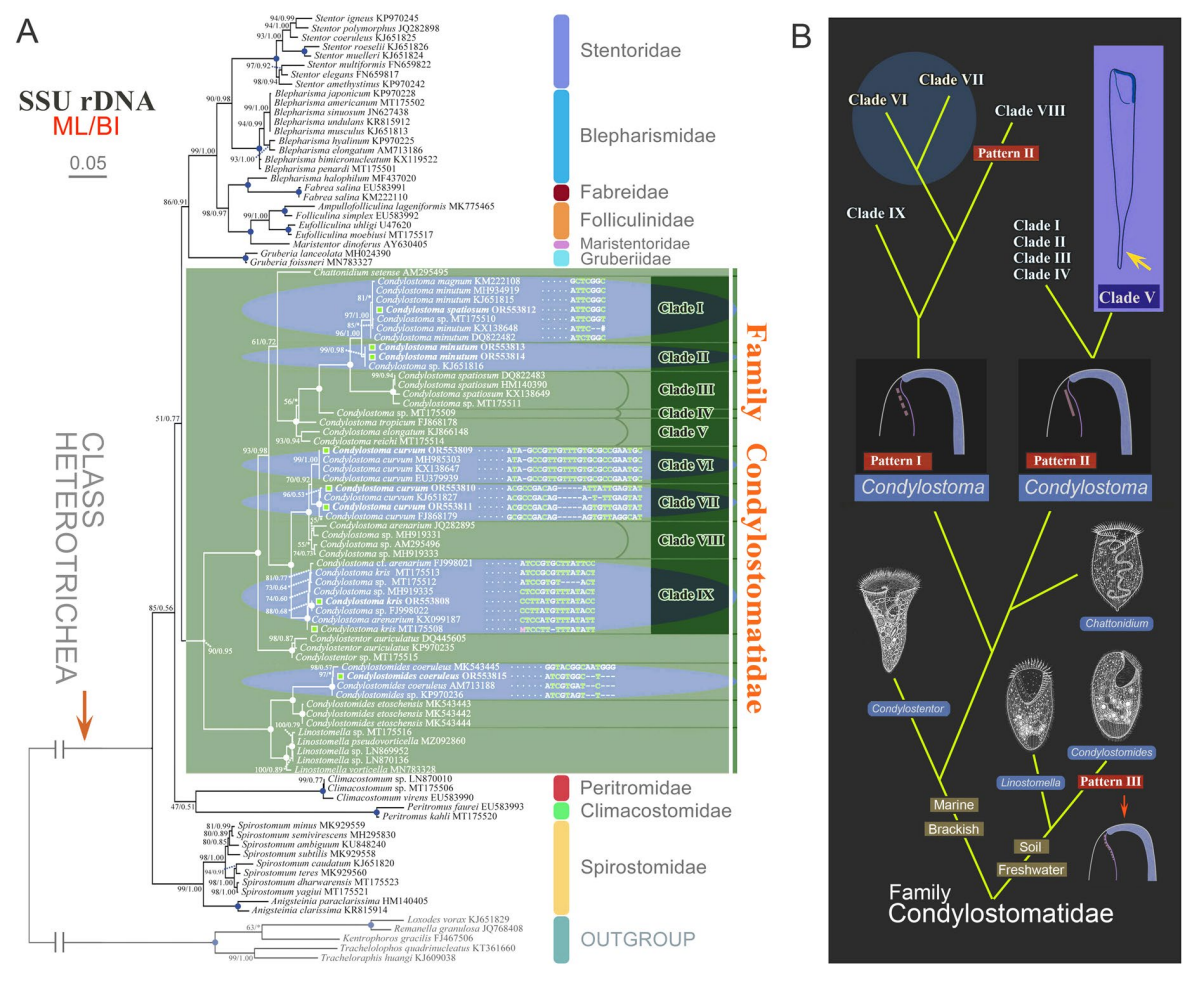
**A** Maximum likelihood (ML) tree based on SSU rDNA sequences of heterotrichous ciliates. Numbers near branches show the bootstrap values from ML and posterior probabilities from Bayesian inference (BI). Disagreements between ML and BI are shown by asterisks. Fully supported clades are marked with solid circles. The nine populations investigated in the present study are marked with a green square before the species, the eight in bold being newly sequenced. The scale bar corresponds to five substitutions per 100 nucleotide positions. Sites not available are shown by octothorpe in the comparison of nucleotide difference. **B** Cladogram of the family Condylostomatidae based on present SSU rDNA phylogenetic tree. Yellow arrow indicates species in Clade V have a tail

[5] 1963 *Condylostoma patulum* Clap. et Lachm., 1858—Dragesco, Cah. Biol. Mar. 4: 255–257 [Fig. 5] (detailed description based on observations of both living and silver stained specimens).

[6] 1999 *Condylostoma patens* Dujardin 1841—Al-Rasheid, Arab Gulf J. Scient. Res. 17: 133 [Fig. 17] (brief redescription without illustration of living cell).

Amended diagnosis: Cell size variable from 500 to 1500 μm; cylindrical and vermiform in shape, widest in mid-region and narrowed slightly towards ends which are rounded; peristome length 1/6–1/5 of cell length; macronucleus moniliform with 7–17 nodules (from drawings); cortical granules rod-shaped, densely arranged between somatic kineties; 50–64 adoral membranelles; 48–80 somatic kineties, usually 50–60; brackish or marine habitat.

Distribution: various kinds of sea water, Denmark (Müller 1786); Sète, France (Dujardin 1841); Bergen, Norway (Claparède and Lachmann 1858); Neuwerk, Sylt, Heligoland, and Kiel, Germany (Kahl 1932); Plounéour-Trez, France (Dragesco 1963); Jubail, Saudi Arabia (Al-Rasheid 1999).

Comments: The type species, *Condylostoma patens*, was originally reported by (Müller 1786) under the name *Trichoda patens*. Although its description is brief, an easily recognizable illustration was provided, which leads us to recognize its identifying features as a cylindrical cell slightly expanding in the middle and the peristome occupying 1/6–1/5 of cell length (Type I). However, numerous subsequent researchers appear to have overlooked this illustration and mistakenly identified a type of organism whose posterior portion expanded into a saccular shape (Type II) as *C. patens* (Calkins 1902; Claparède and Lachmann 1858; Kahl 1932; Stein 1867). Claparède and Lachmann (1858) misidentified Type II as *C. patens* and regarded a population with a relatively smaller peristome as a new species, *C. patulum*. Furthermore, descriptions and illustrations of *C. patulum* provided by the original and subsequent reports (Calkins 1902; Claparède and Lachmann 1858; Kahl 1932; Stein 1867) are consistent with of *C. patens* Type I in terms of cell shape and oral region. Thus, we propose that *C. patulum* is a synonym of *C. patens* and that *C. patens* Type II represents a separate species. In addition, we noticed that in Claparède and Lachmann’s (1858) report on *C. patens* Type II, it was mentioned that “if one day it can be proved that his species is different from the *C. patens* reported by Dujardin (1841), then his species can be named *C. marinum*”. Thus, we here classify individuals with a sac-like posterior end as novel species, *C. marinum* sp. nov.

We need to mention that a Saudi Arabian population with an elongate cell shape, peristome occupying one-fifth of cell length, and 60–73 somatic kineties resembles both *Condylostoma patens* and *C. spatiosum* (Al-Rasheid 1999). This population, however, can be distinguished from *C. spatiosum* by the number of adoral membranelles (50–64 vs. 80–200 in *C. spatiosum*). In addition, the number of adoral membranelles in the type species (*C. patens*) has not been reported, thus we classify this Saudi Arabian population as *C. patens* pending further information.

Maupas (1883) reported an Algerian population under the name of *Condylostoma patens*, however this is inconsistent with *C. patens* in terms of cell size (305–495 μm) and shape (flattened and slender), ratio of peristome to cell length (almost 30%), and number of somatic kineties (about 25), but matches *C. kris* very well, especially in the patterns of frontal membranelles (Pattern I) and cortical granules (Pattern 1). We therefore propose to classify this Algerian population as a synonym of *C. kris*. Several other populations have been reported under the name *C. patens* (Al-Rasheid 1996, 1997; Dragesco 1960; Fauré-Fremiet 1912), however, we could not confidently verify their identity due to the lack of information on key diagnostic characters in these works.

Wilbert and Kahan (1981) found a population collected from Solar Lake (Egypt) and identified it as *Condylostoma patulum*. This population is, however, easily distinguishable from other congeners with a rounded posterior end by its small peristome (1/15–1/10 of cell length), number of adoral membranelles (60–70), number of somatic kineties (36–42), and frontal membranelles composed of six blocks of kinetosomes (Pattern I). Thus, we suggest that this population be classified as a new species, *C. microstomum* sp. nov.

Petz et al. (1995) reported an unidentified *Condylostoma* species from Antarctica, which resembles *C. patens* only by having a vermiform cell that is more than 1 mm long and with a rounded posterior end. However, it can be separated from *C. patens* by the cell shape (anterior right and left margins parallel, gradually narrowed to posterior end vs. anterior slightly narrowed, widest in the middle), arrangement of cortical granules (loose vs. dense), ratio of peristome length to cell length (< 1/6 vs. 1/6–1/5), numbers of adoral membranelles (ca. 180 vs. 50–64) and somatic kineties (39 vs. 48–80), and habitat (hypersaline vs. brackish to marine). We therefore propose that this Antarctic population represents a novel species *C. petzi* sp. nov.

**21.** ***Condylostoma petzi*** **sp. nov.**

[1] 1995 *Condylostoma* sp.—Petz, Stapfia 40: 107, 108 [Fig. 32a–e] (detailed description based on living and protargol-stained specimens, see Comments section of *C. patens*).

Diagnosis: Cell size more than 1200 μm; elongate and vermiform, right and left margins parallel in anterior cell half, posteriorly tapering; peristome length about 15% of cell length; macronucleus moniliform with about 15 nodules; cortical granules large, circular to ellipsoid, colorless, loosely arranged in two rows between adjacent somatic kineties (Pattern 3); adoral zone with about 180 adoral membranelles; about 39 somatic kineties; frontal membranelles composed of a single stripe of kinetosomes (Pattern II); hypersaline habitat.

Type material: A protargol slide has been deposited in the Oberösterreichische Landesmuseum (LI), A-4040 Linz, Austria.

Etymology: We dedicate this species to Dr. Wolfgang Petz, the discoverer.

ZooBank registration: urn:lsid:zoobank.org:act:8A40544F-1C9D-4E2A-83AE-2A6F33F6EE08.

Distribution: Weddell Sea, Antarctica (Petz et al. 1995).

**22.** ***Condylostoma rugosum*** **Kahl, 1932**

[1] 1932 *Condylostoma rugosum* spec. n.—Kahl, Tierwelt Dtl. 25: 455 [Fig. S. 454, 7] (simple original description based on living cells).

Amended diagnosis: Cell size 300–350 μm; elongated with a bluntly rounded posterior end; numerous irregular transverse furrows disrupting somatic kineties on the cortex; peristome length about 1/5 of cell length (from original drawing); macronucleus moniliform; cortical granules rod-shaped, densely arranged between somatic kineties; 20–32 somatic kineties; marine habitat.

Distribution: Sylt, Germany (Kahl 1932).

**23.** ***Condylostoma villeneuvei*** **sp. nov.**

[1] 1940 *Condylostoma arenarium* Spiegel, 1926—Villeneuve-Brachon, Arch. Zool. Exp. Gén. 82: 58–66 [Figs. XXIII–XXIX] (detailed description based on live and stained specimens).

[2] 1972 *Condylostoma arenarium* Spiegel, 1926—Borror, Acta Protozool. 10: 59–61 [Figs. 46, 47] (redescription based on live and stained specimens).

Diagnosis: Cell size 170–480 μm; flattened and elliptical with blunt ends; peristome length about 1/4–1/3 of cell length; macronucleus moniliform with 13–30 nodules, 16–23 vesicular micronuclei; 40–70 somatic kineties; frontal membranelles composed of 4–6 blocks of kinetosomes (Pattern I); brackish or marine habitat.

Type material: Information unavailable.

Etymology: We dedicate this species to Dr. Simone Villeneuve-Brachon, the discoverer.

ZooBank registration: urn:lsid:zoobank.org:act:5ECCC17E-6758-42EF-900C-B245A65CD174.

Distribution: Ditch on the side of the road from Sète to Agde, France (Villeneuve-Brachon 1940); New Hampshire, USA (Borror 1972).

**24.** ***Condylostoma magnum*** **Spiegel, 1926**

[1] 1926 *Condylostoma magnum* nov. spec.—Spiegel, Arch. Protistenkd. 55: 186, 187 [Fig. B] (original description based on living cells).

[2] 1932 *Condylostoma magnum* Spiegel, 1926—Kahl, Tierwelt Dtl. 25: 453, 454 [Fig. S. 454, 2] (short review).

**Fig. 6:**
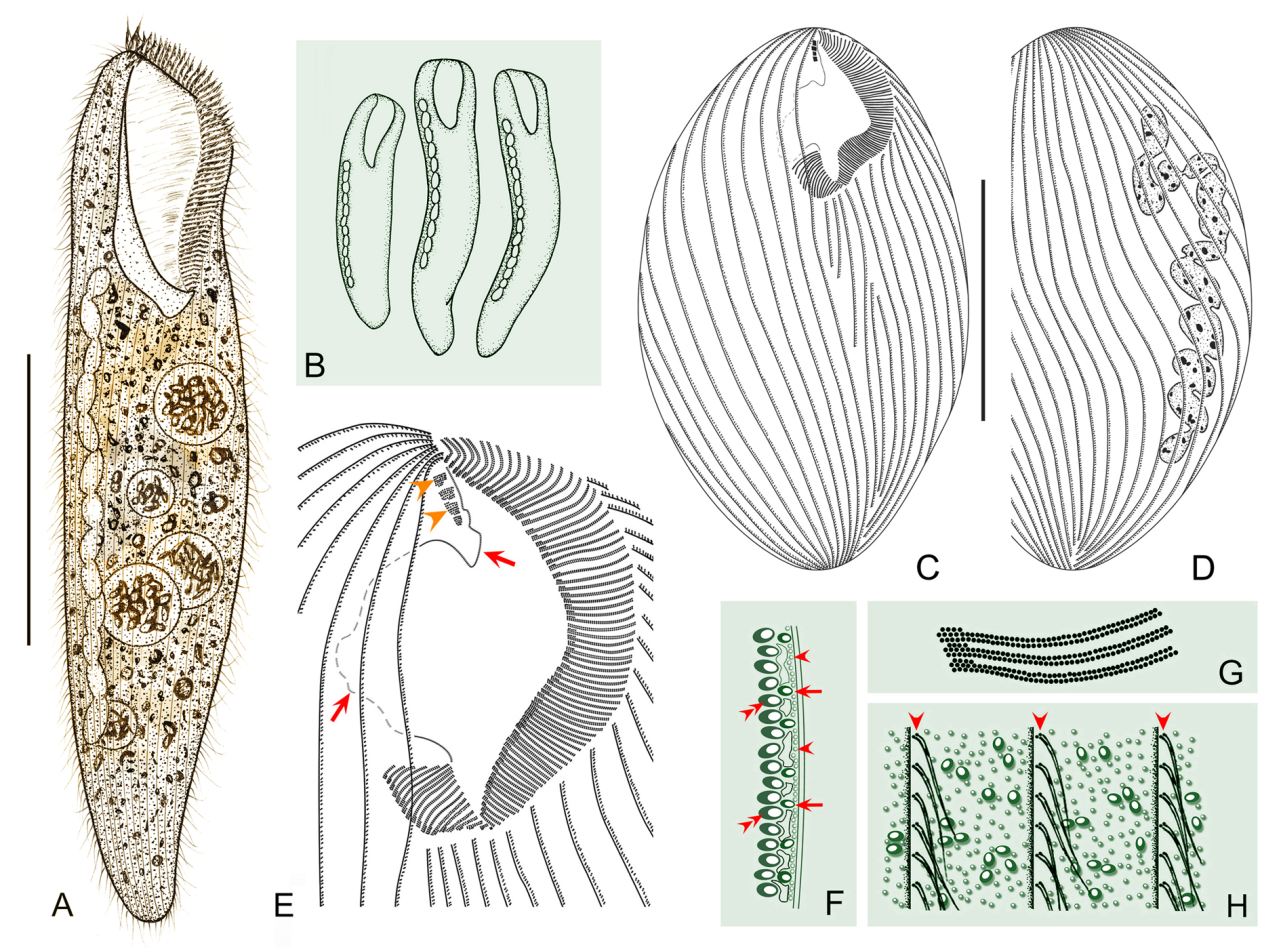
Schematic drawings of Chinese populations of *Condylostoma kris* from life (**A**,** B**,** F**,** H**) and after protargol staining (**C**–**E**, **G**). **A** General view of a representative individual. **B** Different cell shapes and presence of moniliform macronuclei. **C**, **D** Ventral (**C**) and dorsal (**D**) views of the same specimen, showing the ciliature and macronucleus. **E** Detailed view of anterior portion of cell, arrows mark the paroral membrane, arrowheads mark the frontal membranelles. **F** Tangential section of the cortex, arrows mark the ellipsoidal cortical granules (Type I), arrowheads show the spherical cortical granules (Type II), double-arrowheads indicate the ellipsoidal unknown structures (possibly mitochondria). **G** Each adoral membranelle is composed of three rows (two long and one short) of basal bodies. **H** Two types of cortical granules and each basal body of dikinetids bears a cilium (arrowheads). Scale bars: 150 μm

[3] 1940 *Condylostoma magnum* Spiegel 1926—Bullington, Pap. Tortugas Lab. 32: 189–191 [Figs. 5, 6] (detail redescription based on living cells).

[4] 1986 *Condylostoma magnum* Spiegel, 1926—Dragesco & Dragesco-Kernéis, Faune Tropicale: pp 395–397 [Fig. 111] (redescription based on live and silver-stained specimens).

[5] 1999 *Condylostoma magnum* Spiegel 1926—Al-Rasheid, Arab Gulf J. Scient. Res. 17: 132 [Fig. 12] (simple description without illustration of living cell).

Amended diagnosis: Cell size highly variable, 500–1650 μm, usually 500–1100 μm; elongated and flattened with a narrowed posterior portion; peristome length 1/5–1/3 of cell length; macronucleus moniliform with 14–45 nodules, usually 14–24; cortical granules densely arranged in a single row between adjacent somatic kineties; 90–100 adoral membranelles; 72–110 somatic kineties, usually 80–100; frontal membranelles composed of seven blocks of kinetosomes (Pattern I); brackish or marine habitat.

Distribution: Heligoland, Germany (Kahl 1932; Spiegel 1926); Bush Key, Florida, USA (Bullington 1940); Cotonou, Benin (Dragesco and Dragesco-Kernéis 1986); Jubail, Saudi Arabia (Al-Rasheid 1999).

Comments: Song and Wilbert (1997) reported a Chinese population with the following characters: cell size 450–800 μm; peristome about 1/4–1/3 of cell length; 47–56 somatic kineties; about 150–200 adoral membranelles; frontal membranelles composed of a single stripe of kinetosomes (Pattern II). However, this population is distinctly different from the original report by having significantly fewer somatic kineties (47–56 vs. 80) (Spiegel 1926), and can also be clearly distinguished from the Benin population which has seven kinetosomes blocks (Pattern I) (Dragesco and Dragesco-Kernéis 1986). In contrast, the Chinese population matches well with *Condylostoma spatiosum* in various characters except the number of adoral membranelles (150–200 vs. 80–153). Considering that this character is highly variable among different populations of this species, we believe it is reasonable to classify the Chinese population as *Condylostoma spatiosum*. Similarly, an Antarctic population with a small cell size, about 30 somatic kineties, frontal membranelles composed of a single stripe of kinetosomes (Pattern II), and about 120 adoral membranelles was identified by Wilbert and Song (2005) as *Condylostoma* cf. *magnum*. It bears a closer resemblance to *Condylostoma minutum* than *C. magnum*, so we here classify it as *C. minutum*.

**25.** ***Condylostoma microstomum*** **sp. nov.**

[1] 1981 *Condylostoma patulum* Clap. et Lachm. 1858—Wilbert & Kahan, Arch. Protistenkd. 124: 81–85 [Fig. 9] (description based on living cells and protargol preparations; see Comments section of C. patens).

Diagnosis: Cell size 500–800 μm; slender, tapering slightly to rear, with a rounded posterior end; peristome small, 1/15–1/10 of cell length; macronucleus moniliform with 15–24 nodules; 60–70 adoral membranelles; 36–42 somatic kineties; frontal membranelles composed of six blocks of kinetosomes (Pattern I); marine or hypersaline habitat.

Type material: Information unavailable.

Etymology: “*microstomum*” refers to the comparatively small peristome.

ZooBank registration: urn:lsid:zoobank.org:act:58F9D922-40BF-4594-B942-BDE7B5A4A12F.

Distribution: Solar Lake, Egypt (Wilbert and Kahan 1981).

**26.** ***Condylostoma curvum*** **Burkovsky, 1970**

[1] 1932 *Condylostoma arenarium* Spiegel, 1926—Kahl, Tierwelt Dtl. 25: 455 [Fig. S. 454, 6] (containing three different morphotypes; the small form has been listed as a synonym of *C. curvum* by Song et al. 2003).

[2]* 1960 *Condylostoma arenarium* Spiegel—Dragesco, Trav. Stn. Biol. Roscoff (NS) 12: 292, 293 [Fig. 154] (containing two different morphotypes; proposed here that small forms are conspecific with *C. curvum*; see Comments section of *C. arenarium*).

[3] 1961 *Condylostoma arenarium* Spiegel, 1926—Borror, PhD Dissertation pp 67–69 [Figs. 108–110] (simple description based on living cells, synonym proposed by Song et al. 2003).

[4] 1970 *Condylostoma curva* sp. nov.—Burkovsky, Acta Protozool. 8: 58, 59 [Fig. 14] (original description).

[5] 1986 *Condylostoma arenarium* Spiegel, 1926—Dragesco & Dragesco-Kernéis, Faune Tropicale: pp 393–395 [Fig. 110] (description based on live and silver-stained specimens; synonym proposed by Song et al. 2003).

[6]* 1999 *Condylostoma arenarium* Spiegel 1926—Al-Rasheid, Arab Gulf J. Scient. Res. 17: 131, 132 [Fig. 11] (brief redescription based on living and protargol-stained specimens; see Comments section of *C. arenarium*).

[7] 2003 *Condylostoma curva* Burkovsky, 1970—Song et al., J. Eukaryot. Microbiol. 50: 459–461 [Figs. 27–38] (detailed morphological description based on three populations).

[8] 2011 *Condylostoma curva* Burkovsky, 1970—Chen et al., Acta Hydrobiol. Sin. 35: 923 [Figs. 49–53] (redescription from live and protargol-stained specimens).

[9] 2012 *Condylostoma curva* Burkovsky, 1970—Kim et al., Anim. Syst. Evol. Divers. 28: 150–153 [Figs. 1, 2] (redescription based on live and protargol-stained specimens).

[10] 2015 *Condylostoma curvum* Burkovsky, 1970—Yan et al., Eur. J. Protistol. 51: 72–74 [Fig. 5] (mandatory change of gender ending to be consistent with neuter genus name; detailed description, including molecular data).

Improved diagnosis: Cell size variable from 120 to 505 μm, usually 150–400 μm; elongated and flattened with a rounded posterior end; peristome length to cell length variable, 20–45%; macronucleus moniliform with 5–17 nodules, 14–20 micronuclei; cortical granules large, ellipsoidal, brown to grayish green, densely arranged (Pattern 4); 60–115 adoral membranelles; 20–42 somatic kineties; frontal membranelles composed of 4–8 blocks of kinetosomes (Pattern I); brackish or marine habitat.

Distribution: Neuwerk, Germany (Kahl 1932); Alligator Harbor, Florida, USA (Borror 1961); Kandalaksha Bay, Russia (Burkovsky 1970); Lake Nokoue, Cotonou, Benin (Dragesco and Dragesco-Kernéis 1986); Qingdao, (Chen et al. 2011; Song et al. 2003), Guangzhou (Yan et al. 2015), Ningbo (Present study), China; Gyeongsangbuk-do, South Korea (Kim et al. 2012).

Comments: *Condylostoma curvum* was originally reported by Burkovsky (1970), and has been redescribed several times (Chen et al. 2011; Kim et al. 2012; Song et al. 2003; Yan et al. 2015). Considering the high variability in the morphological characters of *Condylostoma*, it is reasonable to summarize the recognizable features of *C. curvum* by combining the characters of all populations with reliable descriptions: (1) small-sized (< 400 μm); (2) cell shape flattened and elongated, posterior end rounded, anterior end slightly truncated; (3) oral region about 25–45% of cell length; (4) macronucleus moniliform; 5) 20–42 somatic kineties; (6) 60–112 adoral membranelles; (7) frontal membranelles with Pattern I arrangement; (8) cortical granules with Pattern 4 arrangement; (9) brackish or marine habitat. The three Chinses populations described in the present study closely resemble the other populations, except for the cell size of population 1 (maximum length 505 μm) (Fig. 9A, D). As cell size is thought to be population-dependent and the average size of population 1 is less than 400 μm, we believe that our identification of the three Chinese populations is correct.

Due to the variable morphological characters among *Condylostoma* species and the lack of information on the frontal membranelles in the original description of *C. arenarium*, subsequent populations of *C. arenarium* were often misidentified as *C. curvum* (see the Comments section of *C. arenarium*). Recently, Fernandes et al. (2015) redefined *C. arenarium* based on modern methods, enabling the differentiation of the two species based on the arrangement of frontal membranelles (Pattern I in *C. curvum* vs. Pattern II in *C. arenarium*) and cell size (small size, typically < 400 μm in *C. curvum* vs. medium size, usually > 400 μm in *C. arenarium*).

Four other *Condylostoma* species with flattened slender cells with a rounded posterior end and Pattern I arrangement of frontal membranelles should be compared with *C. curvum*, namely *C. villeneuvei* sp. nov., *C. magnum*,* C. microstomum* sp. nov., and *C. kris* (Fig. 3). *Condylostoma curvum* can be clearly distinguished from *C. villeneuvei* sp. nov. and *C. magnum* by the number of somatic kineties (20–42 vs. 40–70 in *C. villeneuvei* sp. nov. vs. 72–110 in *C. magnum*) (Borror 1972; Burkovsky 1970; Dragesco and Dragesco-Kernéis 1986; Spiegel 1926; Villeneuve-Brachon 1940). It also can be separated from *C. magnum* by its small-sized cell (< 400 μm vs. 500–1650 μm, usually 500–1100 μm) (Bullington 1940). *Condylostoma microstomum* sp. nov. can be distinguished from *C. curvum* by its cell shape (slender ribbon-like vs. elongated ellipsoidal in *C. curvum*), cell size (500–800 μm vs. 120–505 μm in *C. curvum*), and the ratio of peristome length to cell length (ca. 5–10% vs. 20–45% in *C. curvum*) (Wilbert and Kahan 1981). *Condylostoma kris* is usually medium-sized (> 400 μm vs. small size, < 400 μm, in *C. curvum*) and cortical granules with Pattern 1 (vs. Pattern 4 in *C. curvum*), thus distinguishing it from *C. curvum*.

**27.** ***Condylostoma kris*** **Ozaki & Yagiu in Yagiu, 1944**

[1] 1883 *Condylostoma patens* (Muller spec.).—Maupas, Arch. Zool. Exp. Gén. 1: 521–543 [Fig. XXII, 1–7] (detailed living observation, synonym proposed in present study; see Comments section of *C. patens*).

[2] 1932 *Condylostoma arenarium* Spiegel, 1926—Kahl, Tierwelt Dtl. 25: 455 [Fig. S. 454, 3, 24] (containing three different morphotypes; Type 1 is suggested as a synonym of *C. kris* in the present study).

[3] 1944 *Condylostoma kris* Ozaki & Yagiu, 1942—Yagiu, J. Sci. Hirosima Univ. (Ser. B, Div. 1) 10: 172–177 [Figs. 4, 5, S2, S3] (validated the name proposed at 17th annual meeting of Japanese zoologists in 1942 based on the detailed description).

[4]* 1972 *Condylostoma arenarium* Spiegel, 1926—Agamaliev, Acta Protozool. 10: 19 [Fig. 8] (description based on live and silver-stained specimens, see Comments section of *C. arenarium*).

Improved diagnosis: Cell size 305–700 μm, usually > 400 μm; elongated and flattened with a rounded posterior end; peristome 25–30% of cell length; macronucleus moniliform with 11–21 nodules; two types of cortical granules, Type I, large, ellipsoidal, grayish-green to yellowish-green, loosely arranged, Type II, small, spherical, colorless to grayish, densely arranged (Pattern 1); 80–114 adoral membranelles; 28–48 somatic kineties; frontal membranelles composed of 4–7 blocks of kinetosomes (Pattern I); brackish or marine habitat.

Distribution: Algiers, Algeria (Maupas 1883); Sylt, Germany (Kahl 1932); Onomichi, Japan (Yagiu 1944); Qingdao and Ningbo, China (Present study).

Comments: Like the remarks on *Condylostoma spatiosum* in Shao et al. (2006), *C. kris* was first mentioned at an annual meeting of Japanese zoologists in 1942 and was cited as *C. kris* Ozaki & Yagiu, 1942 in the subsequent detailed description (Yagiu 1944). According to the regulations of ICZN (1999), the original report (1942) is a nomen nudum, and hence the valid name should be cited as *C. kris* Ozaki & Yagiu in Yagiu, 1944.

The original description of *Condylostoma kris*, which was based on fixed material with a certain degree of shrinkage, gives the diagnostic characters as follows: (1) medium size in vivo (325–410 μm in fixed cells); (2) slender ellipsoidal and slightly flattened cell shape; (3) peristome length approximately a quarter of cell length; (4) moniliform macronucleus; (5) 36–48 somatic kineties; (6) about 80 adoral membranelles; (7) kinetosomes of frontal membranelles arranged in Pattern I; (8) pale yellowish-green (depending on microscopy method used) cortical granules, about 1 μm in diameter (0.6–0.8 μm after fixation); (9) marine habitat (Yagiu 1944). Given that the number of adoral membranelles varies among different populations and that the description of the Japanese population was based on both live and fixed cells (without using silver staining methods), we believe the number of adoral membranelles (84–114) falls within a reasonable range for the two Chinese populations. In addition, Yagiu (1944) observed only one type of cortical granules with a loosely-spaced arrangement, which is consistent with Type I of cortical granules in Chinese populations. However, considering the Type II cortical granules are extremely fine and light-colored, these may have been overlooked due to the limitations of the microscopes available. Therefore, we consider the identification of the two Chinese populations as *C. kris* is correct.

As discussed in the Comments section of *Condylostoma curvum*, *C. kris* shares a general appearance with four other species that possess a Pattern I arrangement of frontal membranelles, namely *C. villeneuvei* sp. nov., *C. magnum*, *C. microstomum* sp. nov., and *C. curvum*. *Condylostoma kris* can be clearly separated from *C. villeneuvei* sp. nov. and *C. magnum* by the number of somatic kineties (28–48 vs. 40–70 in *C. villeneuvei* sp. nov. vs. 72–110 in *C. magnum*) (Borror 1972; Dragesco and Dragesco-Kernéis 1986; Spiegel 1926; Villeneuve-Brachon 1940). Additionally, it can be differentiated from *C. villeneuvei* sp. nov. by its cell size (305–700 μm, usually medium-sized vs. 170–480 μm, usually small-sized in *C. villeneuvei* sp. nov.). *Condylostoma microstomum* sp. nov. can be distinguished from *C. kris* by its slenderer, ribbon-like cell (vs. ellipsoidal) and in having a smaller peristome relative to cell length (ca. 5–10% vs. 25–30% in *C. kris*) (Wilbert and Kahan 1981). According to the new observations reported here, *Condylostoma kris* can be distinguished from *C. curvum* by its larger cell size (305–700 μm, usually medium-sized vs. 120–505 μm, usually small-sized in *C. curvum*) and the pattern of cortical granules (Pattern 1 vs. Pattern 4 in *C. curvum*) (Burkovsky 1970; Yan et al. 2015).

**28.** ***Condylostoma spatiosum*** **Ozaki & Yagiu in Yagiu, 1944**

[1] 1944 *Condylostoma spatiosum* Ozaki & Yagiu, 1942—Yagiu, J. Sci. Hirosima Univ. (Ser. B, Div. 1) 10: 163–171 [Figs. 1–3, S1, S3] (validated the name proposed at 17th annual meeting of Japanese zoologists in 1942 based on a detailed description).

[2] 1997 *Condylostoma magnum* Spiegel, 1926—Song & Wilbert, Arch. Protistenkd. 148: 417, 418 [Fig. 3] (misidentification with detailed morphological description, see Comments section of *C. magnum*).

[3] 2006 *Condylostoma spatiosum* Ozaki and Yagiu in Yagiu, 1944—Shao et al., Eur. J. Protistol. 42: 10–19 [Figs. 1–5] (detailed morphological description based on living observation and protargol staining).

[4] 2008 *Condylostoma spatiosum* Ozaki and Yagiu in Yagiu, 1944—Wilbert & Song, J. Nat. Hist. 42: 990 [Fig. 10A, B] (brief redescription).

[5] 2012 *Condylostoma spatiosum* Ozaki and Yagiu in Yagiu, 1944—Kim et al., Anim. Syst. Evol. Divers. 28: 157–159 [Figs. 5, 6] (detailed redescription based on living observation and protargol staining).

Improved diagnosis: Cell size highly variable from 400 μm to more than 1000 μm, usually 400–800 μm; ellipsoidal and dorsoventrally slightly flattened, posterior end tapered and narrowly rounded; ratio of peristome length to cell length variable, from 16 to 40%, usually 25–35%; macronucleus moniliform with 11–31 nodules; cortical granules usually large, ellipsoidal, yellowish-green or dark gray, densely arranged (Pattern 4); 80–200 adoral membranelles; 47–74 somatic kineties; frontal membranelles composed of a single stripe of kinetosomes (Pattern II); brackish or marine habitat.

Distribution: Onomichi, Japan (Yagiu 1944); Qingdao, China (Shao et al. 2006; Song and Wilbert 1997); King George Island, Antarctica (Wilbert and Song 2008); Ulsan, South Korea (Kim et al. 2012); Qingdao, China (Present study).

Comments: *Condylostoma spatiosum* was originally described by Yagiu (1944) with the following characteristics: (1) medium to large size (364–1320 μm in fixed material); (2) cell with a truncated anterior end and a rounded posterior end; (3) peristome length about a quarter of cell length; (4) moniliform macronucleus; (5) 47–64 somatic kineties; (6) about 80 adoral membranelles; (7) three cirri-like projections in front of the paroral membrane; (8) one type of pale yellowish-green (depending on method of microscopy used) cortical granules, about 1 μm in diameter (0.4–0.6 μm after fixation); (9) marine habitat. Our present population closely matches the original description in terms of cell size and shape, general morphology, and habitat, except for the number of adoral membranelles (115–177 vs. ca. 80) and the arrangement of kinetosomes of the frontal membranelles (Pattern II vs. three cirri-like projection, i.e., Pattern I). These differences have also been observed in subsequent redescriptions (Kim et al. 2012; Shao et al. 2006; Song and Wilbert 1997; Wilbert and Song 2008), where a higher number of adoral membranelles (ca. 110–200) and frontal membranelles with Pattern II were reported. It is important to note that the original description was based on both live and fixed cells (without using silver staining methods), which could have led to the overlooking or misinterpretation of these two characters. Considering the consistent redescription of these characters in subsequent reports, we believe that our identification is correct.

There are two additional *Condylostoma* species, namely *C. minutum* and *C. arenarium*, which have a general cell shape that is similar to *C. spatiosum* and a Pattern II arrangement of frontal membranelles. These, however, can be easily differentiated from *C. spatiosum* by the number of somatic kineties (47–74 vs. 26–48 in *C. minutum* and 28–45 in *C. arenarium*) (Bullington 1940; Chen et al. 2007; Fernandes et al. 2015; Kim et al. 2012; Spiegel 1926). In addition, *Condylostoma minutum* is smaller in size (200–435 μm vs. 400 μm to > 1000 μm in *C. spatiosum*), while *Condylostoma arenarium* has a medium-sized cell and generally possesses a shorter buccal field relative to the cell length (20% vs. 25–35% in *C. spatiosum*).

**29.** ***Condylostoma minutum*** **Bullington, 1940**

[1] 1940 *Condylostoma minutum* n. sp.—Bullington, Pap. Tortugas Lab. 32: 193–194 [Fig. 9] (original description based on living cells).

**Fig. 7:**
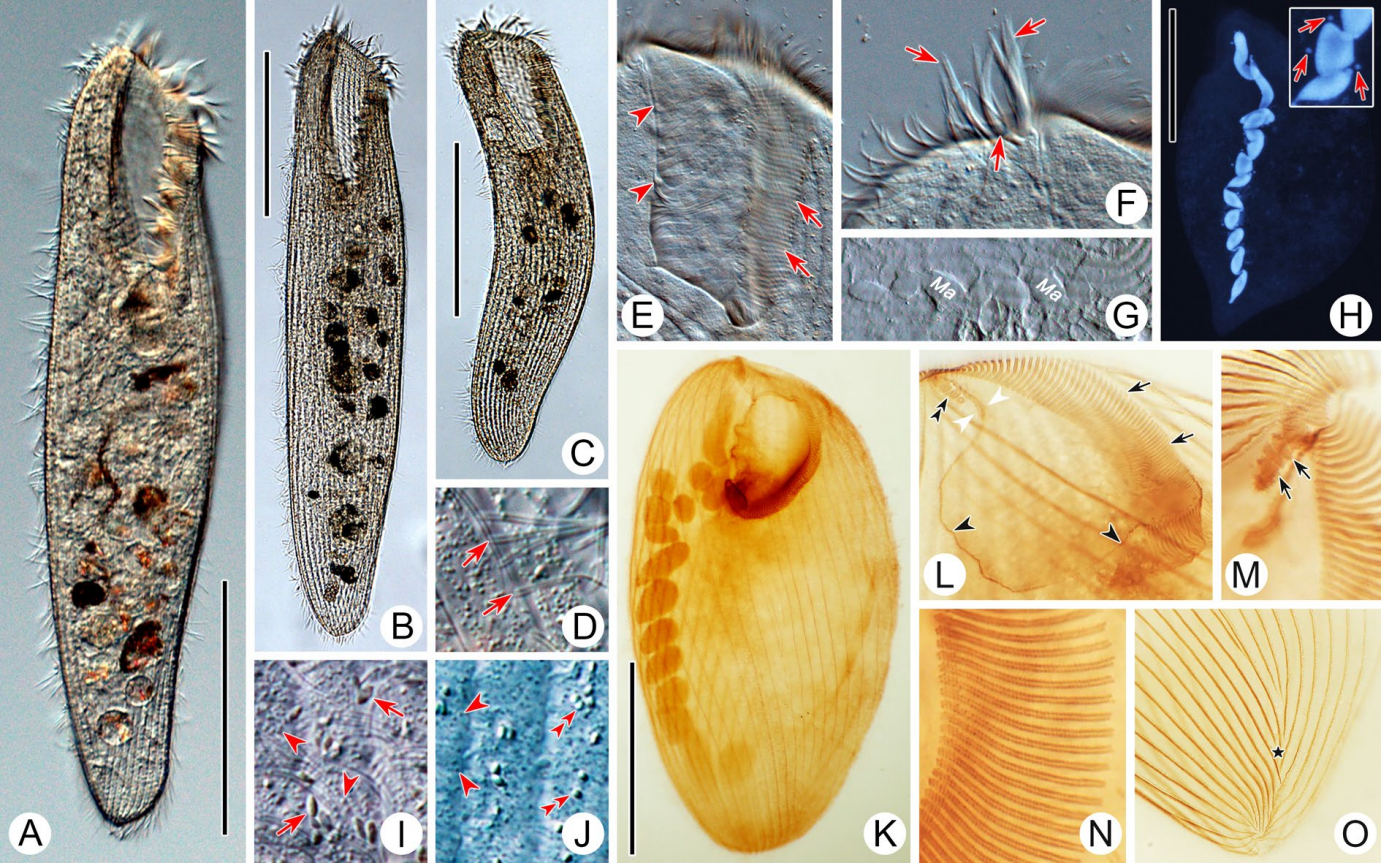
Photomicrographs of *Condylostoma kris* pop.1 from life (**A**–**G**,** I**,** J**), after protargol staining (**K**–**O**), and after Hoechst 33,342 staining (**H**). **A**–**C** Various individuals to show different cell shapes. **D** Details of cilia (arrows). **E** Buccal region, arrows mark the adoral zone of membranelles, arrowheads show the paroral membrane. **F** Anterior portion of cell, arrows mark the frontal membranelles. **G** Moniliform macronucleus. **H** Hoechst 33,342-stained individual to show the nuclear apparatus, arrows mark the micronuclei. **I**,** J** Details of cortex, arrows mark the ellipsoidal unknown structures (possibly mitochondria), arrowheads show the Type II of cortical granules, double-arrowheads indicate the Type I of cortical granules. **K** Ventral view of a representative specimen, to show the ciliature and macronucleus. **L** Ventral view of anterior portion, arrows mark the adoral zone of membranelles, arrowheads show the paroral membrane with occasional slightly broaden distal end (white arrowheads), double-arrowhead indicates the frontal membranelles. **M** Details of frontal membranelles (arrows). **N** Details of adoral membranelles. **O** Ventral view of posterior portion, asterisk marks the suture. *Ma* macronucleus. Scale bars: 150 μm

[2] 2005 *Condylostoma* cf. *magnum* Spiegel, 1926—Wilbert & Song, J. Nat. Hist. 39: 950–952 [Fig. 7A–C] (misidentification based on an Antarctic population, see Comments section of *C. magnum*).

[3] 2007 *Condylostoma minutum* Bullington, 1940—Chen et al., Acta Protozool. 46:299–305 [Figs. 9–12] (detailed morphological description based on living observation and protargol staining).

[4] 2012 *Condylostoma minutum* Bullington, 1940—Kim et al., Anim. Syst. Evol. Divers. 28: 154–157 [Figs. 3, 4] (detailed redescription based on living observation and protargol staining).

Improved diagnosis: Cell size 200–435 μm; elongated and flattened with a rounded posterior end; peristome length about 25–40% of cell length; macronucleus moniliform with 9–22 nodules; cortical granules large, ellipsoidal, greenish-yellow or gray green, densely arranged (Pattern 4); 67–120 adoral membranelles; 26–48 somatic kineties; frontal membranelles composed of a single stripe of kinetosomes (Pattern II); brackish or marine habitat.

Distribution: Bush Key, Florida, USA (Bullington 1940); King George Island, Antarctic (Wilbert and Song 2005); Qingdao, Yantai (Chen et al. 2007), and Ningbo (Present study), China; Jeju-do, South Korea (Kim et al. 2012).

Comments: *Condylostoma minutum* was first described by Bullington (1940) based on observations of cells in vivo, but without details of several diagnostic features. Later, Chen et al. (2007) redescribed it based on three Chinese populations using observations of both live and protargol-stained specimens and provided an improved diagnosis. Combining the original and subsequent investigations (Bullington 1940; Chen et al. 2007; Kim et al. 2012), this species can be recognized by the following characters: (1) small size, 200–390 μm in vivo; (2) cell flattened and slender; (3) peristome length 25–40% of cell length; (4) moniliform macronucleus; (5) 26–44 somatic kineties; (6) 67–107 adoral membranelles; (7) frontal membranelles with Pattern II arrangement; (8) cortical granules with Pattern 4; (9) brackish or marine water habitat. Our present populations closely resemble the previously described populations in terms of all morphological characters with the exception of the color of the cortical granules (gray green vs. dark-gray in Chen et al. 2007 vs. greenish yellow in Kim et al. 2012). As mentioned earlier, this feature usually varies among different observers based on their equipment, such as the magnification combined with the lighting and microscopy technique used, resulting in a rather subjective interpretation. Nevertheless, it still deserves attention in future research as it has been found to be a useful distinguishing character in numerous taxa (Hao et al. 2022; Ma et al. 2022b; Shao et al. 2023).

Within the genus *Condylostoma*, *C. minutum* is most similar to *C. spatiosum* and *C. arenarium* in terms of cell shape and frontal membranelles. However, *C. minutum* can be separated from *C. spatiosum* by its small cell size (200–435 μm vs. 400 μm to > 1000 μm) and in having fewer somatic kineties (26–48 vs. 47–74) (Kim et al. 2012; Shao et al. 2006; Yagiu 1944). Individuals of *C. arenarium* are larger (350–600 μm vs. 200–435 μm) and have a smaller peristome relative to cell size (ca. 20% vs. 25–40%) than *C. minutum* (Fernandes et al. 2015; Spiegel 1926).

**30.** ***Condylostoma arenarium*** **Spiegel, 1926**

[1] 1926 *Condylostoma arenarium* nov. spec.—Spiegel, Arch. Protistenkd. 55: 184–186 [Fig. A] (original description based on living cells).

[2]* 1960 *Condylostoma arenarium* Spiegel—Dragesco, Trav. Stn. Biol. Roscoff (NS) 12: 292, 293 (containing two different morphotypes; large forms without illustration provisionally retained; see Comments section).

[3]* 1973 *Condylostoma arenarium* Spiegel, 1926—Hartwig, Akad. Wiss. Lit., Mainz 18: 44 [Fig. 10] (short description, see Comments section).

[4] 2015 *Condylostoma arenarium* Spiegel, 1926—Fernandes et al., J. Eukaryot. Microbiol. 62: 723–731 [Figs. 1–6] (detailed morphological description and phylogenetic analyses).

Amended diagnosis: Cell size 350–600 μm; elongated ellipsoidal with a slightly tapered posterior end; peristome length about 1/5 of cell length; macronucleus moniliform with 13–20 nodules; cortical granules small, ellipsoidal, green-yellowish, densely arranged (Pattern 6); 83–145 adoral membranelles; 28–45 somatic kineties; frontal membranelles composed of a single stripe of kinetosomes (Pattern II); marine habitat.

Distribution: Heligoland, Germany (Spiegel 1926); Guanabara Bay, Rio de Janeiro, Brazil (Fernandes et al. 2015).

Comments: *Condylostoma arenarium* was originally reported based on living cells but lacked a description of the ciliature on the right side of the paroral membrane (Spiegel 1926). Subsequently, it was identified as having several frontal cirri-like structures (Pattern I) (Borror 1961, 1972; Dragesco and Dragesco-Kernéis 1986; Kahl 1932; Villeneuve-Brachon 1940). However, a recent detailed study, including living observation, protargol staining, scanning electron microscopy, and phylogenetic analyses, redefined *C. arenarium* as having one strip-like “frontal cirri” structure (Pattern II) (Fernandes et al. 2015). Therefore, all populations under the name *C. arenarium*, except for the original and Brazilian populations, need to be reassessed, particularly those with several frontal cirri-like structures (Pattern I).

As discussed in the Comments section of *Condylostoma marinum* sp. nov., Kahl (1932)’s report included three morphotypes, with Types 2 and 3 being synonymous with *C. curvum* and *C. marinum* sp. nov., respectively. Considering the frontal membranelles (Pattern I) and cell size (400–700 μm), *C. arenarium*-like Type 1 corresponds well with *C. kris*, thus we suggest classifying Type1 as a synonym of *C. kris*. We only included cell size in the diagnosis and did not consider other morphological data due to the existence of multiple morphotypes. Additionally, we agree with the proposal by Song et al. (2003) to classify the populations reported by Borror (1961) and Dragesco and Dragesco-Kernéis (1986) as synonyms of *C. curvum*.

It has been suggested in previous studies (Borror 1972; Dragesco and Dragesco-Kernéis 1986; Fernandes et al. 2015) that there may be at least two species within *Condylostoma arenarium*, particularly with regard to the French and American populations (Borror 1972; Villeneuve-Brachon 1940) that possess more somatic kineties (40–70 vs. 28–45) than other populations (Dragesco 1960; Fernandes et al. 2015; Hartwig 1973; Spiegel 1926). Thus we agree with the inferences of Borror (1972) and Dragesco and Dragesco-Kernéis (1986) in proposing the establishment of a new species, *C. villeneuvei* sp. nov., for these two populations.

There are four other populations under the name of *Condylostoma arenarium* in which the ciliature at right side of paroral membrane was undescribed (Agamaliev 1972; Al-Rasheid 1999; Dragesco 1960; Hartwig 1973). There are large overlaps between *C. minutum* and *C. curvum*, as well as between *C. arenarium* and *C. kris*, in terms of cell size and shape, ratio of peristome to cell length, and number of somatic kineties. However, we can only attempt to classify these populations (marked with asterisks in the synonym list) based on weakly supported reasons and have not included the associated morphological data in the diagnoses and geographical distributions. The individuals reported by Dragesco (1960) have two sizes. We propose that the larger one (300–600 μm) be retained in *C. arenarium* because it has only one type of cortical granules (vs. two types in *C. kris*), and that the smaller one (ca. 200 μm) should be classified as *C. curvum* due to its slightly stubby cell shape. Similarly, the North Sea population (Hartwig 1973) which has only one type of cortical granules, should be retained as *C. arenarium*. In contrast, two types of cortical granules appear to present in the drawing of the Azerbaijani population (Agamaliev 1972), which can serve as evidence for provisionally listing it as a synonym of *C. kris*. In his description of *C. arenarium,* Al-Rasheid (1999) noted that it resembles the Cotonou population (Dragesco and Dragesco-Kernéis 1986). Since the latter is here classified as *C. curvum*, thus we suggest that Saudi Arabian population should also be recognized as *C. curvum*.

### Genus *Condylostomides* da Silva Neto, 1994

A clear definition is lacking, therefore an improved diagnosis is provided here based on the present and previous studies.

#### Improved diagnosis

Medium-sized condylostomatid heterotrich, usually 100–300 µm; freely motile, crawling on substrates or between sediment particles. Cell non-contractile, ellipsoidal, obliquely truncated at anterior end. Somatic ciliature holotrichous, including some shortened kineties; suture absent. Buccal area prominent, adoral zone of membranelles at left margin of peristome. Frontal membranelles connected to distal end of paroral membrane. Macronucleus moniliform or composed of two or three ellipsoidal nodules. Contractile vacuole present at posterior end, with two collecting canals extending along margin of cell to anterior end. Cortical granules usually pigmented, mostly yellow or blue-green. Mainly soil or freshwater habitat.

#### Type species

*Condylostomides tardus* (Penard, 1922) Foissner et al., 2002

#### Identification characteristics

*Condylostomides* species had been discovered before the genus was established in 1994, however, they were classified as *Condylostoma* due to their close resemblance in general morphology, particularly the prominent buccal area (da Silva Neto 1994; Dragesco 1954, 1960; Foissner 1995; Kahl 1932; Penard 1922). While *Condylostoma* species are commonly found in marine or brackish water, members of *Condylostomides* typically inhabit freshwater or soil habitat. A notable distinguishing feature is the presence of a contractile vacuole at the posterior end in *Condylostomides*, which is absent in *Condylostoma*. Additionally, protargol-stained specimens reveal further distinctions between the two genera, such as the absence of suture in *Condylostomides* (present in *Condylostoma*), the ciliation of dikinetids (only one basal body ciliated in *Condylostomides* vs. both basal bodies ciliated in *Condylostoma*), and the arrangement of kinetosomes of the frontal membranelles (Pattern III in *Condylostomides* vs. Patterns I and II in *Condylostoma*).

Currently, the macronucleus and cortical granules, i.e., their shape, arrangement, and color, serve as important identifying features within the genus *Condylostomides* (Foissner et al. 2002). Based on the review of *Condylostoma* and *Condylostomides* in the present work, we propose the transfer of *Condylostoma minima* to *Condylostomides*, resulting in a total of eight valid *Condylostomides* species (Fig. 3).

#### Key to valid *Condylostomides* species

Note: The numbers within parentheses correspond to the order of species in synonym lists, diagnoses, and distributions.

- **1** Moniliform macronucleus…………………………………………………………………………… **3**
  - **1.1** Three macronuclear nodules …………………………………………………………………. **2**
  - **1.2** Two macronuclear nodules ……………………………………………………….(1) *C. luteus*
- **2** Colorless cortical granules …………………………………………………………..…..(2) *C. tardus*
  - **2.1** Citrine cortical granules ……………………………………………………(3) *C. trinucleatus*
- **3** One type of cortical granules …………………………………………………………………….… **4**
  - **3.1** Two types of cortical granules …………………………………………(4) *C. nigrus* nom. corr.
- **4** Yellow or inconspicuous cortical granules ………………………………………………………… **5**
  - **4.1** Blue-green cortical granules …………………………………………………(5) *C. coeruleus*
- **5** Ellipsoidal to spindle-shaped cortical granules arranged in loosely spaced rows …………………. **6**
  - **5.1** Spherical cortical granules arranged in closely spaced rows ……………….(6) *C. etoschensis*
- **6** 15–18 somatic kineties ……………………………………………………………….(7) *C. terricola*
  - **6.1** 20–22 somatic kineties ………………………………(8) *C. minimus* comb. nov. & nom. corr.

#### Synonym lists, diagnoses, and distributions of all valid *Condylostomides* species

Note: In the list of synonyms for each species, we have directly cited the unaltered species names (as well as author names) from the original sources. This is to demonstrate the transition of species names from historical references to the contemporary era, despite several identifiable errors that are subsequently present throughout.

**1.** ***Condylostomides luteus*** **(Kahl, 1932) Foissner et al., 2002**

[1] 1932 *Condylostoma luteum* spec. n.—Kahl, Tierwelt Dtl. 25: 457 [Fig. S. 454, 8] (original description based on live and stained cells).

[2] 2002 *Condylostomides luteus* (Kahl, 1932) nov. comb.—Foissner et al., Denisia 5: 899 (species transfer, no new description).

Diagnosis summarized from original population: Cell size 100–130 μm; narrowly sac-shaped, anterior portion slightly widened, posterior end bluntly rounded; peristome about 1/3 of cell length; two macronuclear nodules; one type of yellowish cortical granules arranged in approximately four closely spaced rows between adjacent kineties; freshwater habitat.

Distribution: Hamburg, Germany (Kahl 1932).

**2.** ***Condylostomides tardus*** **(Penard, 1922) Foissner et al., 2002**

[1] 1922 *Condylostoma tardum* sp. n.—Penard, Georg & Cie pp 202–204 [Fig. 201] (original description based on living cells).

[2] 1932 *Condylostoma tardum* Penard, 1922—Kahl, Tierwelt Dtl. 25: 457 [Fig. S. 454, 11, 23] (simple redescription based on living cells).

[3] 1960 *Condylostoma tardum* Pénard—Dragesco, Trav. Stn. Biol. Roscoff (NS) 12: 298, 299 [Fig. 159] (short redescription based on a French population).

[4] 1994 *Condylostomides grolieri* gen. n., sp. n.—da Silva Neto, Acta Protozool. 33: 150–156 [Figs. 1–31] (establishment of new genus based on detailed morphological description, junior synonym proposed and declared as the type species).

[5] 2002 *Condylostomides tardus* (Penard, 1922) nov. comb.—Foissner et al., Denisia 5: 899 (species transfer, no new description).

Amended diagnosis: Cell size 170–400 μm; ellipsoidal with a wide anterior part; peristome length about 1/3–2/5 of cell length; three macronuclear nodules; cortical granules colorless; about 30 adoral membranelles; 27–35 somatic kineties; freshwater habitat.

Distribution: Geneva (four sites: l'Ariana, Florissant, Bernex, Rouelbeau), Switzerland (Penard 1922); Hamburg and Oldesloe, Germany (Kahl 1932); Lake Geneva, Excenevex (near Thonon), France (Dragesco 1960); Auvergne, France (da Silva Neto 1994).

Comments: The genus *Condylostomides* was established by da Silva Neto (1994) with *C. grolieri* as the type species, however, the author did not make any comparisons with other similar taxa. We agree with the analyses of Foissner et al. (2002) that: (1) *C. grolieri* should be listed as a junior synonym of *C. tardus*; (2) *C. tardus* possesses colorless cortical granules. As a result, we agree with the change of the type species of this genus to *C. tardus*.

**3.** ***Condylostomides trinucleatus*** **Foissner et al., 2002**

[1] 2002 *Condylostomides trinucleatus* nov. spec.—Foissner et al., Denisia 5: 899–904 [Figs. 195, 408] (detailed morphological description based on living observations, protargol preparations, and scanning electron microscopy).

Diagnosis summarized from original population: Cell size 170–270 × 100–135 μm; ellipsoidal to bursiform, anterior end bluntly pointed, posterior end broadly rounded; peristome about 2/5 of cell length (from original drawing); invariably three macronuclear nodules; one type of cortical granules, brilliant-citrine; 25–40 adoral membranelles; 30 somatic kineties on average (exact number could not be determined); soil or freshwater habitat.

Distribution: Murray River, Australia; Bukaos River and Bambatsi Guest Farm, Namibia (Foissner et al. 2002).

**4.** ***Condylostomides nigrus*** **(Dragesco, 1960) nom. corr.**

[1] 1960 *Condylostoma nigra* n. sp.—Dragesco, Trav. Stn. Biol. Roscoff (NS) 12: 297, 298 [Fig. 158] (original description based on living cells).

[2]* 1996 *Condylostoma nigra* Dragesco 1960—Al-Rasheid, Arab Gulf J. Sci. Res. 14: 757 [Fig. 3k] (short description).

[3] 2016 *Condylostomides nigra* Dragesco, 1960—Foissner, Denisia 35: 867 (species transfer, no new description).

Diagnosis summarized from original population: Cell size 180–300 μm; ellipsoid, oral portion slightly widened; peristome large, about 45% of cell length; macronucleus moniliform with around 10 nodules; two types of cortical granules: Type I large, fusiform, blue, Type II small, oval, colorless; about 40 somatic kineties; freshwater habitat.

ZooBank registration: urn:lsid:zoobank.org:act:1326A00C-9CB0-4E3B-92DC-26C1EE63ADEE.

Distribution: Lake Geneva, Excenevex (near Thonon), France (Dragesco 1960).

Comments: In the original report (Dragesco 1960), it was mentioned that there were numerous “vacuoles pulsatiles” on the edge of the cell, which Foissner (2016) later considered to be small contractile vacuoles. We speculate this species has only one contractile vacuole at the posterior end, similar to other congeners, and that other small contractile vacuoles could be either food vacuoles or the expansion of collecting canals as shown in the illustrations for *Condylostomides tardus* in Dragesco (1960) and *Condylostomides coeruleus* in the present study (Fig. 15C). Al-Rasheid (1996) reported a Saudi Arabian population as *Condylostoma nigra* (marked with asterisks in the synonym list), but with limited morphological data to support this identification. Therefore, we did not include this population’s morphological data in the diagnosis as its identity cannot be verified. Foissner (2016) transferred *Condylostoma nigra* to *Condylostomides* when comparing with *C. coeruleus*. As the genus *Condylostomides* is masculine gender, we propose a compulsory change to the name, i.e., *Condylostomides nigrus* (Dragesco, 1960) nom. corr.

**5.** ***Condylostomides coeruleus*** **Foissner, 2016**

[1] 2016 *Condylostomides coeruleus* nov. spec.—Foissner, Denisia 35: 851–867 [Figs. 290–294] (detailed morphological description from living observations, protargol preparations, scanning electron microscopy, and a phylogenetic analysis).

[2] 2020 *Condylostomides coeruleus* Foissner, 2016—Hines et al., Protist 171: 3–8 [Figs. 2, 3] (redescription based on living cells and phylogenetic analyses).

Improved diagnosis: Cell size 110–315 × 40–155 μm; ellipsoidal, obliquely truncated at anterior oral apparatus; peristome large, about 45% of cell length; macronucleus moniliform with 6–13 nodules; one type of cortical granules, blue-green, broadly ellipsoidal, arranged in closely spaced rows between kineties; 37–52 adoral membranelles; 34–52 somatic kineties; soil or freshwater habitat.

Distribution: Morrocoy National Park, Chichiriviche, Venezuela; Costa Rica; Dominican Republic (Foissner 2016); Fort Pierce, Florida, USA (Hines et al. 2020); Lake Weishan, Jining, China (Present study).

Comments: *Condylostomides coeruleus* was originally discovered in slightly saline mud and soil and was recognized by its large size and conspicuous color (Foissner 2016). Hines et al. (2020) rediscovered it in Florida, USA, with diagnostic features matching the original description, except they reported a smaller cell width of 40–64 μm (mean 55 μm). This can be explained by measurements taken in vivo while swimming from soil cultures rather than from fixed cells, and overall slight variation in size within a population. The Weishan population is the first record of this species from Asia and matches closely with the original description in all morphological characters, leaving little doubt as to its identity.

An additional *Condylostomides* species with blue cortical granules, *C. nigrus* nom. corr., should be compared with *C. coeruleus.* The former has a second type of cortical granules that are densely arranged and colorless (Dragesco 1960) and so can easily be distinguished from the latter, which has only one type of cortical granules.

**6.** ***Condylostomides etoschensis*** **Foissner et al., 2002**

[1] 2002 *Condylostomides etoschensis* nov. spec.—Foissner et al., Denisia 5: 893–899 [Figs. 194, 406, 407] (detailed morphological description based on living observations, protargol preparations, and scanning electron microscopy).

[2] 2020 *Condylostomides etoschensis* Foissner et al., 2002—Hines et al., Protist 171: 3–8 [Figs. 1, 3] (redescription based on living cells and the first phylogenetic analyses).

Amended diagnosis: Cell size 160–310 × 70–150 μm; ellipsoidal, posterior end broadly rounded, anterior end bluntly pointed and slightly projecting above ventral surface; peristome about 35–50% of cell length; macronucleus moniliform with 5–12 nodules; one type of cortical granules, citrine, arranged in closely spaced rows between somatic kineties; 33–67 adoral membranelles; 30–42 somatic kineties; soil habitat.

Distribution: Etosha Pan, Namibia; Cotonou, Benin (Foissner et al. 2002); Fort Pierce, Florida, USA (Hines et al. 2020).

Comments: It is noteworthy that this distinct and colorful soil species was, like *C. coeruleus*, thought to be an endemic ciliate with a restricted distribution upon its first discovery in Africa. Despite its apparent absent from South American soil surveys, and indeed the present study in China, it was discovered in Florida on the North American continent (Hines et al. 2020).

**7.** ***Condylostomides terricola*** **(Foissner, 1995) Foissner et al., 2002**

[1] 1995 *Condylostoma terricola* nov. spec.—Foissner, Arch. Protistenkd. 145: 59–63 [Figs. 86–97] (original description based on live and silver-stained specimens).

[2] 2002 *Condylostomides terricola* (Foissner, 1995) nov. comb.—Foissner et al., Denisia 5: 899 (species transfer, no new description).

Diagnosis summarized from original population: Cell size 90–140 × 30–60 μm; narrowly to broadly sac-shaped, posterior end broadly rounded; peristome about 1/3 of cell length (from original drawing); macronucleus moniliform with 4–8 nodules; one type of cortical granules, yellowish, ellipsoidal, arranged in loose rows interspersed with small clusters; 33–40 adoral membranelles; 15–18 somatic kineties; soil habitat.

Distribution: Santa Rosa National Park, Costa Rica (Foissner 1995).

**8.** ***Condylostomides minimus*** **(Dragesco, 1954) comb. nov. & nom. corr.**

[1] 1954 *Condylostoma minuta* n. sp.—Dragesco, Bull. Soc. Zool. Fr. 79: 68, 69 [Fig. 3b] (original description based on living cells).

[2] 1960 *Condylostoma minima* nom. nov.—Dragesco, Trav. Stn. Biol. Roscoff (NS) 12: 296, 297 [Fig. 157] (simple redescription, a name correction suggested to replace the previously occupied name).

Amended diagnosis: Cell size 130–220 μm; ellipsoidal, obliquely truncated anterior end, widest in middle portion; peristome large, about 40–50% of cell length; macronucleus moniliform with six or seven nodules; one type of spindle-shaped cortical granules arranged in loosely spaced rows between kineties; 20–22 somatic kineties.

ZooBank registration: urn:lsid:zoobank.org:act:570CBC5A-C958-4E21-82E5-4404F321EE98.

Distribution: Carantec, France (Dragesco 1954); Roscoff, France (Dragesco 1960).

Comments: This species is characterized by a terminal contractile vacuole, which is the most distinguishing character between *Condylostoma* and *Condylostomides*. In addition, its small cell size, ellipsoidal cell shape, large oral region, and comparatively few macronuclear nodules make it consistent with the diagnosis of *Condylostomides*. Thus, we suggest that this species should be transferred to *Condylostomides* as *Condylostomides minimus* (Dragesco, 1954) comb. nov. & nom. corr. to match the masculine genus. It should be noted that both reported populations of this species were collected from sand, although the exact environmental parameters of these sands were not precisely reported (Dragesco 1954, 1960). Also, as Dragesco (1960) mentioned, the salinity of sandy habitats is highly variable due to the influence of freshwater flow, rainfall, and evaporation, therefore further sampling of potential habitats of this species is needed, despite the original descriptions being collected within a marine beach area.

Three other congeners are brightly colored and have a moniliform macronucleus, namely *C. etoschensis*, *C. coeruleus*, and *C. nigrus* nom. corr. *Condylostomides minimus* comb. nov. & nom. corr. can be distinguished from all of these by having loosely arranged spindle-shaped cortical granules (vs. closely arranged minute cortical granules in *C. etoschensis* vs. closely arranged ellipsoidal cortical granules in *C. coeruleus* vs. two types of cortical granules in *C. nigrus* nom. corr.) and about 20 somatic kineties (vs. 30–42 in *C. etoschensis* vs. 34–52 in *C. coeruleus* vs. ca. 40 in *C. nigrus* nom. corr.) (Dragesco 1960; Foissner 2016; Foissner et al. 2002). *Condylostomides terricola* most closely resembles *C. minimus* comb. nov. & nom. corr. Since the color of cortical granules for *C. minimus* comb. nov. & nom. corr. is not listed, it is assumed to be inconspicuous like other ciliates without mention of pigments (vs. bright yellowish cortical granules in *C. terricola*). In addition, *C. terricola* can still be separated from *C. minimus* comb. nov. & nom. corr. by its smaller size (90–140 μm vs. 130–220 μm), smaller proportion of peristome (ca. 33% vs. 40–50%), and in having fewer somatic kineties (15–18 vs. 20–22) (Dragesco 1954, 1960; Foissner 1995). Therefore, we consider that *C. minimus* comb. nov. & nom. corr. is a valid species.

### Geographic distribution

The global distribution patterns of *Condylostoma* and *Condylostomides* are derived from the data generated from the review of all valid species recognized in the present studies (Fig. 4A). Both genera have been recorded on multiple continents, with the exceptions of *Condylostoma* in Oceania and *Condylostomides* in Antarctica. Europe is the predominant region for records of *Condylostoma*, with more than 70% of the species recorded from this continent. Additionally, approximately 40% of *Condylostoma* species have been found thus far in Asia. In contrast, some members of the genus *Condylostomides* were at first considered representative biogeographical flagship species (Foissner 2016; Foissner et al. 2002). For example, *C. etoschensis* was recorded from Africa and was thought to be restricted there, especially after similar sampling from South America did not recover this species. However, like other species that benefit from intensive sampling, it was later recorded from another geographical region, in this case Florida, USA (Hines et al. 2020). Similarity, *C. coeruleus* was at first found in South America, but later was also found in Florida, USA. Notably, in the present study, we report the first record of *C. coeruleus* on the Asian continent, providing further evidence for the global distribution of *Condylostomides* and indeed ciliates in general when sampling efforts are increased (Finlay 2002).

*Condylostoma* species are characterized as large heterotrichous ciliates with a prominent buccal region (Chen et al. 2007; Chi et al. 2021). This distinctive buccal region allows them to be macrophagous omnivores capable of feeding on a variety of organisms, including diatoms, flagellates, green algae, other ciliates, and small metazoans (Fenchel 1968). Their broad dietary range indicates their adaptability to diverse environments, enabling them to inhabit brackish and marine waters, and even extreme habitats such as those with high salinity and in extreme temperatures found in polar regions. In contrast, the habitats of *Condylostomides* differ completely from those of *Condylostoma*. They are primarily found in terrestrial soils and freshwater environments, occasionally exhibiting some degree of salinity tolerance. It is noteworthy that only two *Condylostoma* species (*C. kasymovi* and *C. caudatum*) have been recorded from freshwater habitats, and no further redescriptions have been reported since their original descriptions. Additionally, the full extent of geographical distribution for *Condylostomides* is not fully understood due to undersampling. Therefore, broader sampling efforts are needed to further explore the occurrence, distribution, and population-specific morphology of these two genera.

### Molecular data and phylogenetic analyses

In the present study, eight new SSU rDNA sequences were obtained and deposited in the GenBank database. The lengths, GC-contents, and accession numbers of these sequences are provided in Table 1. The maximum likelihood (ML) and Bayesian inference (BI) trees based on SSU rDNA sequences are generally congruent, and therefore, only the ML tree with support values from both analyses is presented (Fig. 5A).

Within the class Heterotrichea, eight families, i.e., Stentoridae, Fabreidae, Folliculinidae, Gruberiidae, Condylostomatidae, Peritromidae, Climacostomidae, and Spirostomidae, are revealed to be monophyletic (Fig. 5A). The exceptions are Maristentoridae, which are monotypic, and Blepharismidae, which are paraphyletic with *Blepharisma halophilum* grouping in the family Fabreidae. The five genera with available molecular data within the family Condylostomatidae are divided into two lineages. One lineage consists of *Condylostentor*, *Chattonidium*, and *Condylostoma*, which inhabit brackish or marine habitats. The other lineage comprises *Condylostomides* and *Linostomella*, which are commonly found in soil or freshwater habitats (Fig. 5B). Three genera, namely *Condylostentor*, *Condylostomides*, and *Linostomella*, are monophyletic, while *Condylostoma* are paraphyletic due to the monotypic genus *Chattonidium* nesting within it.

*Condylostoma* species are generally divided into nine clades (Clades I–IX). The newly sequenced *Condylostoma spatiosum* (OR553812) clusters within Clade I, alongside *Condylostoma* sp. (MT175510), *C. magnum* (KM222108), and *C. minutum* (MH934919, KJ651815, KX138648, and DQ822482), rather than Clade III, which consists of *Condylostoma* sp. (MT175511) and *C. spatiosum* (DQ822483, HM140390, and KX138649). Similarly, the two newly sequenced populations of *Condylostoma minutum* (OR553813 and OR553814) form a separate clade (Clade II) together with an unidentified species (MT175511), rather than clustering with the existing *C. minutum* sequences in the GenBank database. The three species, i.e., *C. spatiosum*, *C. minutum*, *and C. magnum*, are very similar in overall appearance (Chen et al. 2007; Shao et al. 2006; Spiegel 1926), making it challenging to distinguish them in vivo. In addition, none of the existing sequences of these species are supported by morphological information or vouchered specimens, raising the potential for misidentification. Based on these analyses, we suggest that: Clade I, exhibiting 0–4 nucleotide differences, represents *C. spatiosum*; Clade II represents *C. minutum*; and Clade III represents a *C. spatiosum*-like species. However, future studies combing both morphological and molecular data are needed to further explore the identifications and classifications of these species within their respective clades.

Although the three newly sequenced Chinese populations of *Condylostoma curvum* (OR553809, OR553810, and OR553811) cluster with other populations of *C. curvum*, they are divided into two distinct clades (Clades VI and VII). The presence of 21–25 stable nucleotide differences between Clade VI and Clade VII supports their significant divergence. Despite this differentiation, the current knowledge and morphometric data obtained from in vivo observations and protargol staining do not allow us to classify them as separate species. This suggests the existence of cryptic species within *C. curvum*, similar to the findings reported for *Spirostomum minus* and *S. teres* (Boscaro et al. 2014; Chi et al. 2020a, 2022b). To accurately determine and define the boundaries of the *C. curvum* species complex, a multidisciplinary integrative approach combining traditional taxonomy and modern molecular technologies is required (Serra et al. 2020).

All populations of *Condylostoma kris* are clustered in Clade IX, which also includes *Condylostoma* sp., *Condylostoma* cf. *arenarium*, and *C. arenarium*. *Condylostoma arenarium* was redefined based on a Brazilian population using an integrative approach including in vivo observation, protargol staining, scanning electron microscopy, and phylogenetic analysis (Fernandes et al. 2015). This Brazilian population (JQ28289) is placed within Clade VIII in our phylogenetic tree. In the literature, many descriptions of populations under the name *C. arenarium* have been recognized as having a “blocky” arrangement of frontal membranelles (Pattern II), which is same as the frontal membranelles of *C. kris*, leading to potential misidentifications of these two species. However, it is noteworthy that the sequence divergence within Clade IX, ranges from 0 to 10 nucleotide differences. Therefore, broader sampling with more detailed morphological information and reliable molecular data is needed to determine whether Clade IX exclusively comprises *C. kris*.

The genus *Condylostomides* is divided into two fully supported clades. One clade consists of three populations of *Condylostomides coeruleus*, which includes the newly sequenced Chinese population (OR553815), as well as an unidentified species (KP970236). The other clade solely comprises the three sequences from a single population of *Condylostomides etoschensis*. However, the Chinese population differs from the American (MK543445) and Venezuelan (AM713188) populations by 10 and 3 nucleotide sites, respectively. These differences may contribute to the inconsistency of the topologies between the ML and BI trees with additional samples being necessary to fully investigate any potential differences between different populations.

Finally, mapping the two patterns of frontal membranelles onto the phylogenetic tree reveals that *Condylostoma* species are roughly divided into two major groups according to different patterns (Fig. 5B). One group (G1) contains Clades I–V and Clade VIII (*C. arenarium*), all of which are Pattern II. The other group (G2) consists of Clade VI, Clade VII, and Clade IX, all of which are Pattern I. Interestingly, *Chattonidium setense* possesses a distinct bundle of cilia in the same location as the frontal membranelles of *Condylostoma* species (Modeo et al. 2006). Based on scanning electron micrographs, this structure appears to be arranged in a striped pattern, suggesting a potential homology with the Pattern II of frontal membranelles in *Condylostoma* (G2). These homologous structures may play important roles in establishing the sister relationship between *Chattonidium setense* and G2 of *Condylostoma*. However, the frontal membranelles of *Condylostomides* do not correspond well with the two patterns observed in *Condylostoma*. It is nearly connected to the paroral membrane and does not seem to be a fully independent structure. As a result, the frontal membranelles serve not only as a crucial taxonomic feature, but also likely play a significant role in the evolution of the family Condylostomatidae. To further investigate and verify this hypothesis, broader sampling from different genera, including detailed morphological characters and robust molecular data, are required.

### Morphological description of Chinese populations

#### ***Condylostomakris* Ozaki & Yagiu in Yagiu, 1944**

***Voucher slides:*** All voucher slides with protargol-stained specimens were deposited in the Laboratory of Protozoology, Ocean University of China (OUC). The number of voucher slides and registration numbers of each population are as follows: *C. kris* pop.1, five slides, CY2019011601-01–05; *C. kris* pop.2, seven slides, YTT2019071502-01–07.

***Description of population 1:*** Cell size 385–560 × 85–110 μm in vivo, on average about 450 × 95 μm with length to width ratio about 5:1, slightly contractile. Cell narrowed ellipsoidal and flattened, truncated at anterior end, widest in mid-region and gradually tapered to rounded posterior end (Figs. 6A, 7A–C). Macronucleus moniliform, composed of 11–15 nodules, located along right side of cell; numerous micronuclei, closely associated with macronuclear nodules (Figs. 6B, D, 7G, H; Table 2). Pellicle flexible and thin, with two types of cortical granules: Type I, ellipsoidal, grayish-green, loosely arranged, about 1.5 μm in length; Type II, spherical, colorless, densely arranged, about 0.5 μm in diameter (Figs. 6F, H, 7I, J). Unknown structures (possibly mitochondria) ellipsoidal, dark gray, about 2.0 μm in length, located underneath cortex (Figs. 6F, 7I). Cytoplasm colorless to slight yellowish at low magnification, with numerous small granules and food vacuoles filled with algae (Figs. 6A, 7A–C). Locomotion by gliding over substratum, occasionally swimming while rotating about the main cell axis.

**Table 2:** Morphometric data of the nine Chinese populations of *Condylostoma kris*, *C. curvum*, *C. spatiosum*, *C. minutum*, and *Condylostomides coeruleus* from protargol-stained specimens

Character	Population	Min	Max	Mean	M	SD	CV	N
Cell, length (μm)	*C. kris* pop.1	269	465	355.6	341	53.0	14.9	27
	*C. kris* pop.2	252	535	379.0	350	85.8	22.6	21
	*C. curvum* pop.1	207	451	312.3	306	61.5	19.7	21
	*C. curvum* pop.2	180	320	240.5	239	35.5	14.7	21
	*C. curvum* pop.3	204	351	282.5	279	41.9	14.8	21
	*C. spatiosum*	264	598	395.9	397	68.4	17.3	25
	*C. minutum* pop.1	232	372	320.1	328	40.0	12.5	21
	*C. minutum* pop.2	225	450	326.3	316	62.5	19.2	21
	*C. coeruleus*	213	317	250.8	243	29.6	11.8	21
Cell, width (μm)	*C. kris* pop.1	164	253	209.7	209	22.6	10.8	27
	*C. kris* pop.2	103	256	159.1	148	42.8	26.9	21
	*C. curvum* pop.1	59	230	115.0	102	42.6	37.0	21
	*C. curvum* pop.2	76	143	107.7	109	20.1	18.6	21
	*C. curvum* pop.3	79	148	105.8	105	15.6	14.7	21
	*C. spatiosum*	104	217	136.4	130	28.1	20.6	25
	*C. minutum* pop.1	67	108	90.9	92	10.9	12.0	21
	*C. minutum* pop.2	86	184	121.8	119	24.9	20.5	21
	*C. coeruleus*	129	229	170.6	171	26.4	15.5	21
Oral area, length (μm)	*C. kris* pop.1	78	119	101.9	101	10.3	10.1	25
	*C. kris* pop.2	70	133	95.0	93	15.9	16.8	21
	*C. curvum* pop.1	47	97	71.4	72	13.3	18.5	21
	*C. curvum* pop.2	56	80	66.4	66	6.9	10.4	21
	*C. curvum* pop.3	62	103	81.1	80	12.2	15.0	21
	*C. spatiosum*	100	186	142.9	142	22.7	15.9	25
	*C. minutum* pop.1	75	110	90.8	90	9.4	10.3	21
	*C. minutum* pop.2	75	137	109.0	113	14.8	13.6	21
	*C. coeruleus*	94	140	112.4	112	11.3	10.0	21
Adoral membranelles, number	*C. kris* pop.1	90	114	98.7	97	6.3	6.4	21
	*C. kris* pop.2	84	108	94.8	96	7.0	7.4	21
	*C. curvum* pop.1	79	115	96.5	98	9.6	9.9	21
	*C. curvum* pop.2	75	95	84.5	85	5.8	6.9	21
	*C. curvum* pop.3	63	90	75.9	75	7.0	9.2	21
	*C. spatiosum*	115	177	139.9	142	15.9	11.4	21
	*C. minutum* pop.1	82	108	96.6	98	7.5	7.8	21
	*C. minutum* pop.2	85	108	95.9	97	5.8	6.1	21
	*C. coeruleus*	43	52	48.0	48	2.4	5.0	21
SK, number (including shortened SK)	*C. kris* pop.1	36	45	38.8	38	2.7	7.0	18
	*C. kris* pop.2	28	43	34.9	34	4.1	11.7	21
	*C. curvum* pop.1	30	41	33.7	33	2.8	8.3	21
	*C. curvum* pop.2	32	38	35.5	36	1.7	4.9	21
	*C. curvum* pop.3	27	32	29.2	29	1.5	5.1	21
	*C. spatiosum*	51	75	60.1	60	6.4	10.7	23
	*C. minutum* pop.1	38	44	41.2	41	1.8	4.4	21
	*C. minutum* pop.2	41	48	43.5	43	2.1	4.8	21
	*C. coeruleus*	42	52	46.0	45	2.9	6.2	21
Shortened SK, number	*C. kris* pop.1	4	15	10.0	10	3.5	35.0	17
	*C. kris* pop.2	4	11	6.7	7	2.0	29.5	21
	*C. curvum* pop.1	6	11	7.7	7	1.2	16.1	21
	*C. curvum* pop.2	5	12	7.1	6	2.0	27.4	21
	*C. curvum* pop.3	5	9	5.9	5	1.3	21.4	15
	*C. spatiosum*	6	33	18.0	17	7.0	38.7	21
	*C. minutum* pop.1	6	12	7.9	7	1.8	22.7	21
	*C. minutum* pop.2	5	12	7.5	8	1.8	24.0	10
	*C. coeruleus*	4	8	5.6	5	1.3	23.2	21
Ma nodules, number	*C. kris* pop.1	11	15	12.7	13	1.3	10.5	9
	*C. kris* pop.2	12	19	14.8	14	1.7	11.4	19
	*C. curvum* pop.1	7	13	9.3	9	1.5	16.0	21
	*C. curvum* pop.2	5	10	7.6	8	1.3	16.9	21
	*C. curvum* pop.3	NA	NA	NA	NA	NA	NA	NA
	*C. spatiosum*	13	25	17.4	17	2.7	15.7	23
	*C. minutum* pop.1	12	18	15.1	15	2.2	14.4	19
	*C. minutum* pop.2	11	22	16.7	16	2.9	17.5	21
	*C. coeruleus*	6	10	7.5	8	1.1	14.0	21
Ma nodules, length (μm)^a^	*C. kris* pop.1	22	52	36.1	35	9.3	25.8	9
	*C. kris* pop.2	20	68	32.2	28	13.5	41.8	19
	*C. curvum* pop.1	23	57	37.6	35	9.1	24.2	21
	*C. curvum* pop.2	16	59	33.1	30	9.8	29.8	21
	*C. curvum* pop.3	NA	NA	NA	NA	NA	NA	NA
	*C. spatiosum*	11	40	25.2	24	6.7	26.6	23
	*C. minutum* pop.1	12	23	16.7	16	3.3	19.9	19
	*C. minutum* pop.2	13	56	25.1	22	9.8	39.0	21
	*C. coeruleus*	14	34	24.4	24	4.6	19.0	21
Ma nodules, width (μm)^a^	*C. kris* pop.1	11	30	20.7	20	5.1	24.5	9
	*C. kris* pop.2	11	41	19.9	18	8.3	41.5	19
	*C. curvum* pop.1	16	39	25.1	24	6.1	24.5	21
	*C. curvum* pop.2	14	32	22.1	22	5.4	24.3	21
	*C. curvum* pop.3	NA	NA	NA	NA	NA	NA	NA
	*C. spatiosum*	9	22	15.5	16	3.7	23.6	23
	*C. minutum* pop.1	7	15	10.8	11	2.1	19.7	19
	*C. minutum* pop.2	9	26	15.1	15	5.2	34.4	21
	*C. coeruleus*	9	20	16.8	18	2.6	15.5	21

*CV* coefficient of variation in %, *M* Median, *Ma* macronucleus, *Max* maximum, *Mean* arithmetic mean, *Min* minimum, *N* number of specimens, *NA* not available, *SD* standard deviation, *SK* somatic kineties

^a^Macronuclear nodules were selected haphazardly in each specimen

Thirty-six to 45 somatic kineties composed of dikinetids, both basal bodies of each dikinetid bear a cilium, respectively (Fig. 7D). Most kineties extend along complete length of cell, with 4–15 shortened kineties both on ventral and dorsal sides (Figs. 6C, D, H, 7K). Several shortened kineties form a conspicuous suture on ventral side near posterior end of cell (Figs. 6C, 7O).

Buccal field prominent, occupies 25–30% of cell length (Figs. 6A, B, 7A–C). Adoral zone of membranelles located on left of buccal field, beginning at apical end and extending spirally into cytopharynx. About 90–114 adoral membranelles, each consisting of one short and two long rows of basal bodies (Figs. 6C, E, G, 7E, L, N). Paroral membrane conspicuous and visible in vivo, occasionally slightly broaden at distal end (Figs. 6E, 7L). Frontal membranelles located at distal end of paroral membrane, consist of 4–7 blocks formed by aggregations of kinetosomes (Pattern I) (Figs. 6E, 7F, L, M).

***Description of population 2***: The morphology of population 2 (Fig. S1A–D) is generally consistent with that of population 1. The main differences are as follows: (1) cell length in vivo (335–540 μm, on average about 405 μm vs. 385–560 μm, on average about 450 μm); (2) number of somatic kineties (variable, 28–43 vs. constant, 36–45); and (3) habitat (brackish vs. marine).

#### ***Condylostomacurvum* Burkovsky, 1970**

***Voucher slides:*** All voucher slides with protargol-stained specimens were deposited in the Laboratory of Protozoology, Ocean University of China (OUC). The number of voucher slides and registration numbers of each population are as follows: *C. curvum* pop.1, six slides, YTT2019120401-01–06; *C. curvum* pop.2, four slides, YTT2019112802-01–04; *C. curvum* pop.3, three slides, YTT2020050101-01–03.

**Fig. 8:**
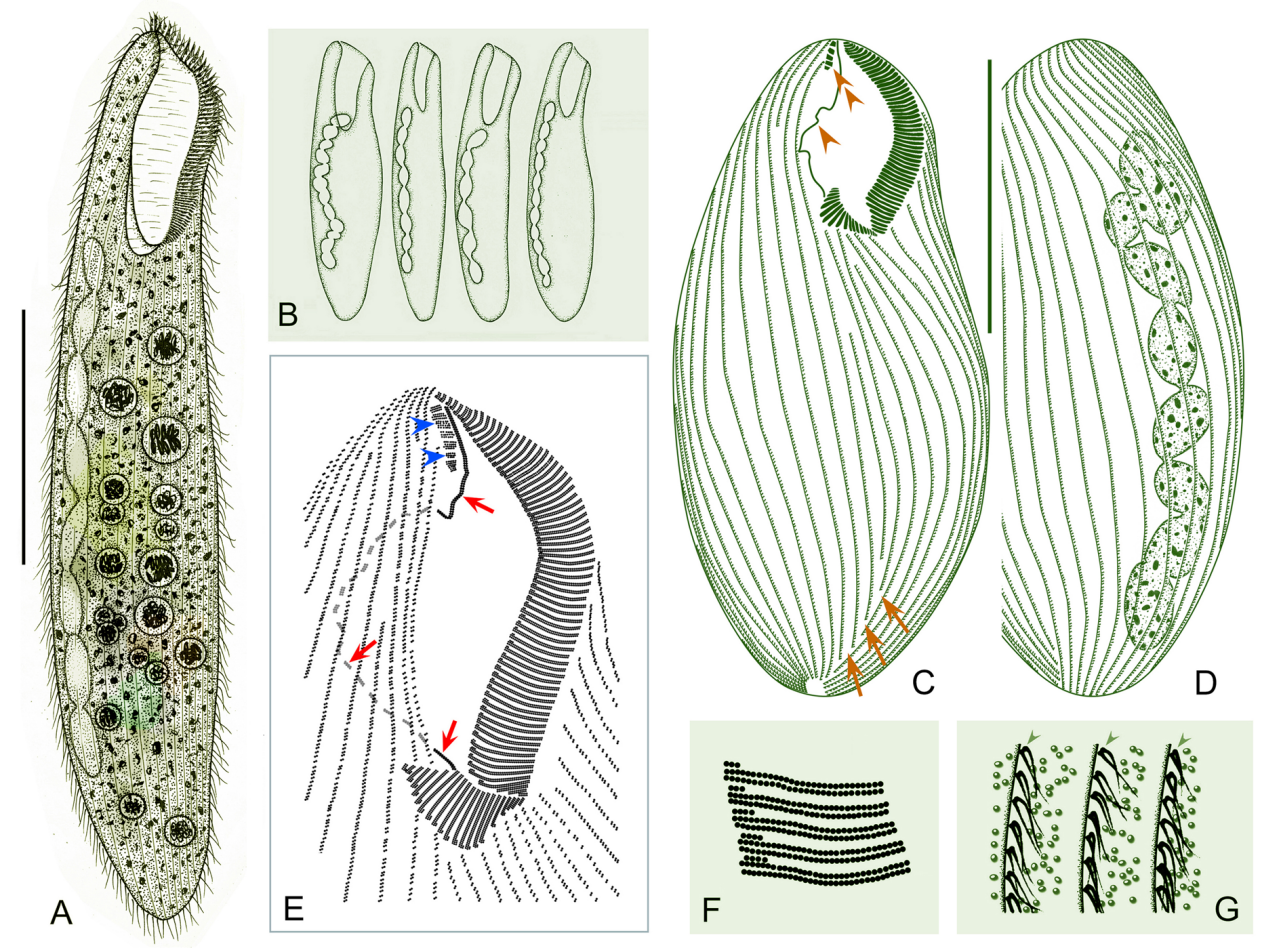
Schematic drawings of Chinese populations of *Condylostoma curvum* from life (**A**,** B**,** G**) and after protargol staining (**C**–**F**). **A** General view of a typical individual.** B** Different cell shapes and moniliform macronuclei.** C**, **D** Ventral (**C**) and dorsal (**D**) views of the same specimen, showing the suture (arrows), paroral membrane (arrowhead), frontal membranelles (double-arrowhead), moniliform macronucleus. **E** Detailed view of anterior portion of cell, arrows mark the paroral membrane, arrowheads mark the frontal membranelles.** F** Each adoral membranelle is composed of three rows (two long and one short) of basal bodies. **G** Showing cortical granules and that each basal body of dikinetids bears a cilium (arrowheads). Scale bars: 100 μm

**Fig. 9:**
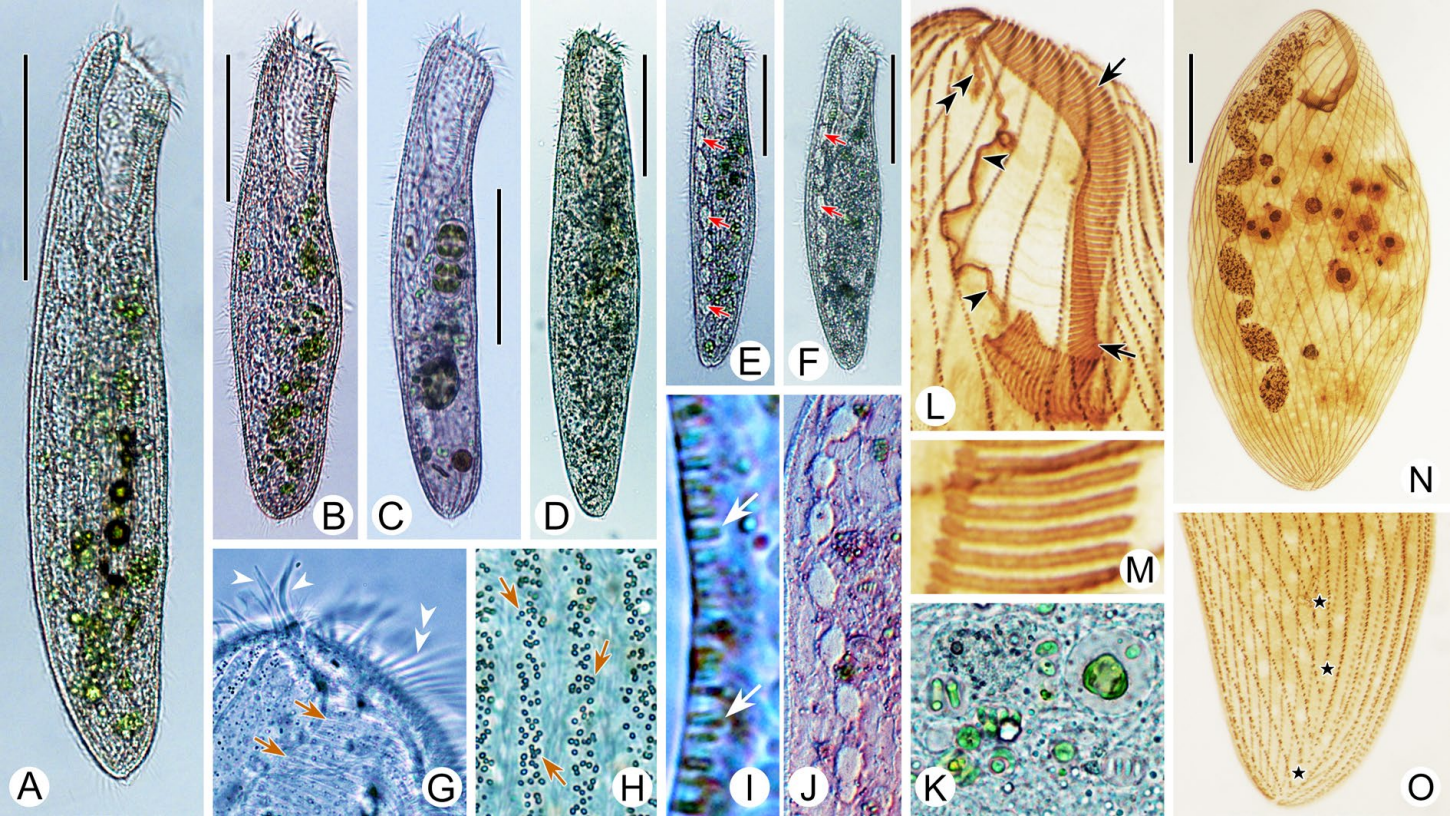
Photomicrographs of *Condylostoma curvum* from life (**A**, **D**, **G**–**K** from pop.1; **E**, **F** from pop.2; **B**, **C** from pop.3) and after protargol staining (**L**–**O** from pop.1).** A**–**F** Various individuals to show different cell shapes, arrows mark the moniliform macronucleus. **G** Anterior portion of cell, arrows mark the paroral membrane, arrowheads show the frontal membranelles, double-arrowhead indicates the adoral membranelles. **H** Cortical granules (arrows) between adjacent somatic kineties. **I** Tangential section of the cell, arrows mark the ellipsoidal cortical granules. **J** Detail of the moniliform macronucleus. **K** Food vacuoles containing algae. **L** Ventral view of anterior portion, arrows mark the adoral zone of membranelles, arrowheads show the paroral membrane, double-arrowhead indicates the frontal membranelles.** M** Details of adoral membranelles.** N** A representative specimen, to show the ciliature and macronucleus.** O** Ventral view of posterior portion, asterisks mark the suture. Scale bars: 100 μm

***Description of population 1:*** Cell size 315–505 × 55–95 μm in vivo, on average about 395 × 70 μm, with length to width ratio about 5:1. Cell elongate-ellipsoidal and dorsoventrally flattened, with a truncated anterior end and a narrowly rounded posterior end (Figs. 8A, B, 9A, D). Macronucleus moniliform, composed of 7–13 nodules, aligned along the right margin of cell; micronuclei difficult to recognize either in vivo or in protargol preparations (Figs. 8A, B, 9J, N; Table 2). Pellicle rough with ellipsoidal, brownish-green cortical granules (about 1 μm in length), distributed with medium density between somatic kineties (Figs. 8G, 9H, I). Cytoplasm brownish at low magnification, with numerous small granules and a few food vacuoles containing algae (Figs. 8A, 9A, K). Movement relatively slow, usually gliding over substrate, occasionally swimming.

Thirty to 41 somatic kineties including 6–11 shortened rows on both ventral and dorsal sides (Figs. 8C, D, 9N). All somatic kineties composed of dikinetids; each basal body of dikinetids bears a cilium (Fig. 8C, D, G). Suture on ventral side of posterior portion of cell formed by shortened somatic kineties (Figs. 8C, 9O).

Buccal field about 25–30% of cell length (Figs. 8A, B, 9A, D). Adoral zone composed of 79–115 membranelles, each consisting of one short and two long rows of basal bodies (Figs. 8E, F, 9L, M). Paroral membrane highly developed and visible in vivo (Figs. 8C, E, 9G, L). Frontal membranelles composed of 5–8 blocks formed by aggregations of kinetosomes (Pattern I) (Figs. 8C, E, 9L).

***Description of populations 2 and 3:*** The key identifying characters of populations 2 (Fig. 9E, F; Supplementary Fig. S1E–H) and 3 (Fig. 9B, C; Supplementary Fig. S1I–L) are almost identical to those of population 1. Considering that *Condylostoma curvum* is a species complex (as shown in Fig. 5A), the detailed differences between these populations are provided here: (1) cell length in vivo (315–505 μm on average 395 μm in pop.1 vs. 295–390 μm on average 330 μm in pop.2 vs. 285–340 μm on average 315 μm in pop.3); (2) number of adoral membranelles (79–115 on average 97 in pop.1 vs. 75–95 on average 85 in pop.2 vs. 63–90 on average 76 in pop.3); (3) habitat salinity (19.1‰ for pop.1 vs. 4.0‰ for pop.2 vs. 4.5‰ for pop.3).

#### ***Condylostomaspatiosum* Ozaki & Yagiu in Yagiu, 1944**

***Voucher slides:*** Four voucher slides with protargol-stained specimens were deposited in the Laboratory of Protozoology, Ocean University of China (OUC) with registration numbers: CY2019041301-01–04.

**Fig. 10:**
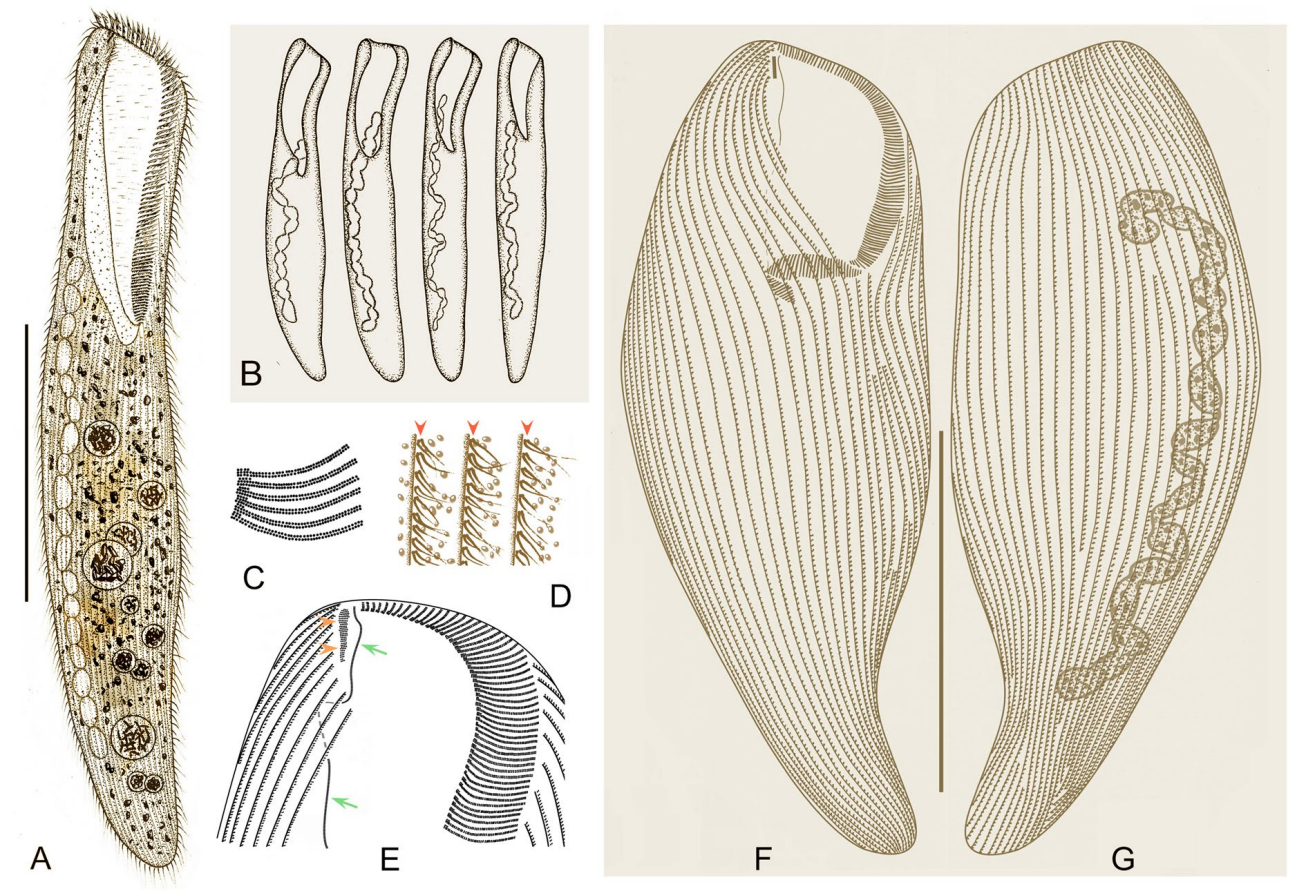
Schematic drawings of Chinese population of *Condylostoma spatiosum* from life (**A**,** B**, **D**) and after protargol staining (**C**, **E**–**G**).** A** General view of a typical individual.** B** Different cell shapes and moniliform macronuclei.** C** Each adoral membranelle is composed of three rows (two long and one short) of basal bodies.** D** Showing cortical granules and that each basal body of dikinetids bears a cilium (arrowheads). **E** Detailed view of anterior portion of cell, arrows mark the paroral membrane, arrowheads mark the frontal membranelles.** F**, **G** Ventral (**F**) and dorsal (**G**) views of same specimen, showing the ciliature and macronucleus. Scale bars: 200 μm

**Fig. 11:**
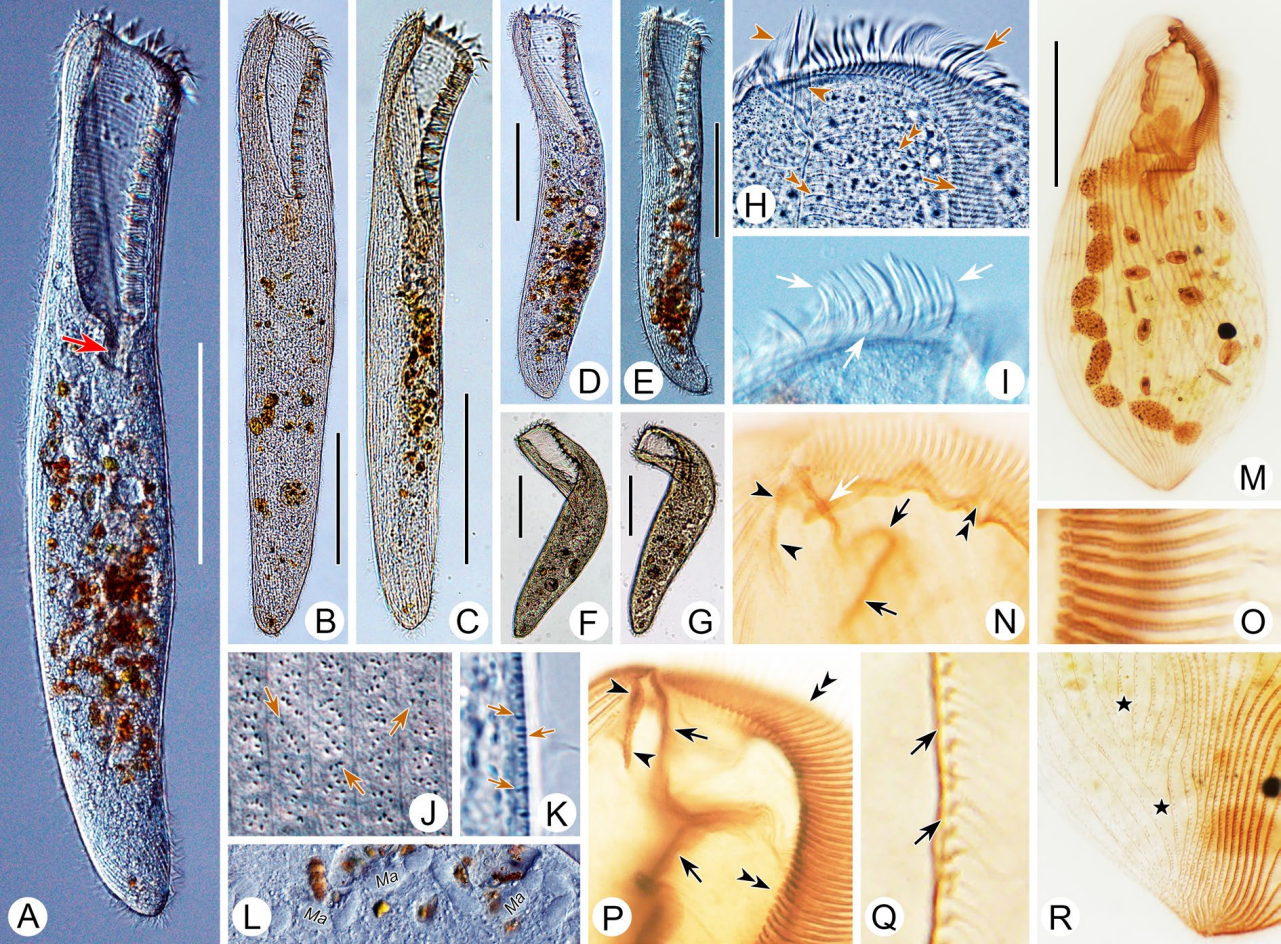
Photomicrographs of Chinese population of *Condylostoma spatiosum* from life (**A**–**L**) and after protargol staining (**M**–**R**).** A**–**G** Various individuals showing different cell shapes, arrow marks the cytopharynx. **H** Anterior portion of cell, arrows mark the adoral zone of membranelles, arrowheads show the paroral membrane, double-arrowheads indicate the fiber-like stripes. **I** Details of frontal membranelles (arrows). **J** Cortical granules (arrows) between adjacent somatic kineties. **K** Tangential section of the cell, arrows mark the ellipsoidal cortical granules. **L** Moniliform macronucleus. **M** Ventral side of a representative specimen, to show the ciliature and macronucleus. **N**,** P** Ventral view of anterior portion, arrows mark the paroral membrane with occasional slightly broadened distal end (white arrow), arrowheads show the frontal membranelles, double-arrowheads indicate the adoral zone of membranelles. **O** Details of adoral membranelles. **Q** Each basal body of dikinetids (arrows) bears a cilium. **R** Ventral view of posterior portion, asterisks mark the suture. *Ma* macronucleus. Scale bars: 100 μm (**M**), 150 μm (**A**–**G**)

***Description:*** Cell size usually 540–710 × 65–115 μm in vivo, on average about 610 × 100 μm; length to width ratio on average about 6:1. Extended cell shape elongate-ellipsoidal, dorsoventrally slightly flattened, anterior end truncated, posterior portion gradually tapered with a rounded end (Figs. 10A, B, 11A–E). Macronucleus moniliform composed of 13–25 (usually 17) nodules, aligned along right margin of cell; micronucleus not detected either in vivo or in protargol preparations (Figs. 10A, B, 11L, M; Table 2). Pellicle flexible and slightly contractile (Fig. 10F, G), with ellipsoidal, dark gray cortical granules (about 1–1.5 μm in length), irregularly arranged with medium density between somatic kineties (Figs. 10D, 11J, K). Cytoplasm somewhat opaque at low magnifications due to numerous inclusions and food vacuoles (Figs. 10A, 11A–G). Movement by gliding on Petri dish bottom, occasionally swimming while rotating about main cell axis.

Fifty-one to 75 longitudinal somatic kineties, of which 6–33 (on average 18) are shortened or fragmented rows both on ventral and dorsal sides, resulting it highly variable numbers of somatic kineties (Figs. 10F, G, 11M). Somatic kineties composed of densely spaced dikinetids; each basal body of dikinetids bears a cilium (Figs. 10D, 11Q). Suture on ventral side near posterior end of cell formed by several shortened kineties (Figs. 10F, G, 11R).

Buccal field about 25–40% of cell length, with a conspicuous cytopharynx (Figs. 10A, B, 11A–E). Adoral zone comprises 115–177 membranelles, each composed of one short and two long rows of basal bodies, twisted in proximal region (Figs. 10C, E, F, 11H, M–P). Transversely arranged fiber-like stripes visible on inner wall of oral cavity (Fig. 11H). Paroral membrane highly developed and visible in vivo, occasionally slightly broaden at distal end (Figs. 10E, 11H, N, P). Frontal membranelles located to right of distal end of paroral membrane, consist of a single stripe formed by aggregations of kinetosomes (Pattern II) (Figs. 10E, F, 11I, N, P).

#### ***Condylostomaminutum* Bullington, 1940**

***Voucher slides:*** All voucher slides with protargol-stained specimens were deposited in the Laboratory of Protozoology, Ocean University of China (OUC). The number of voucher slides and registration numbers of each population are as follows: *C. minutum* pop.1, four slides, YTT2019071101-01–04; *C. minutum* pop.2, eight slides, LJ2018111501-01–08.

**Fig. 12:**
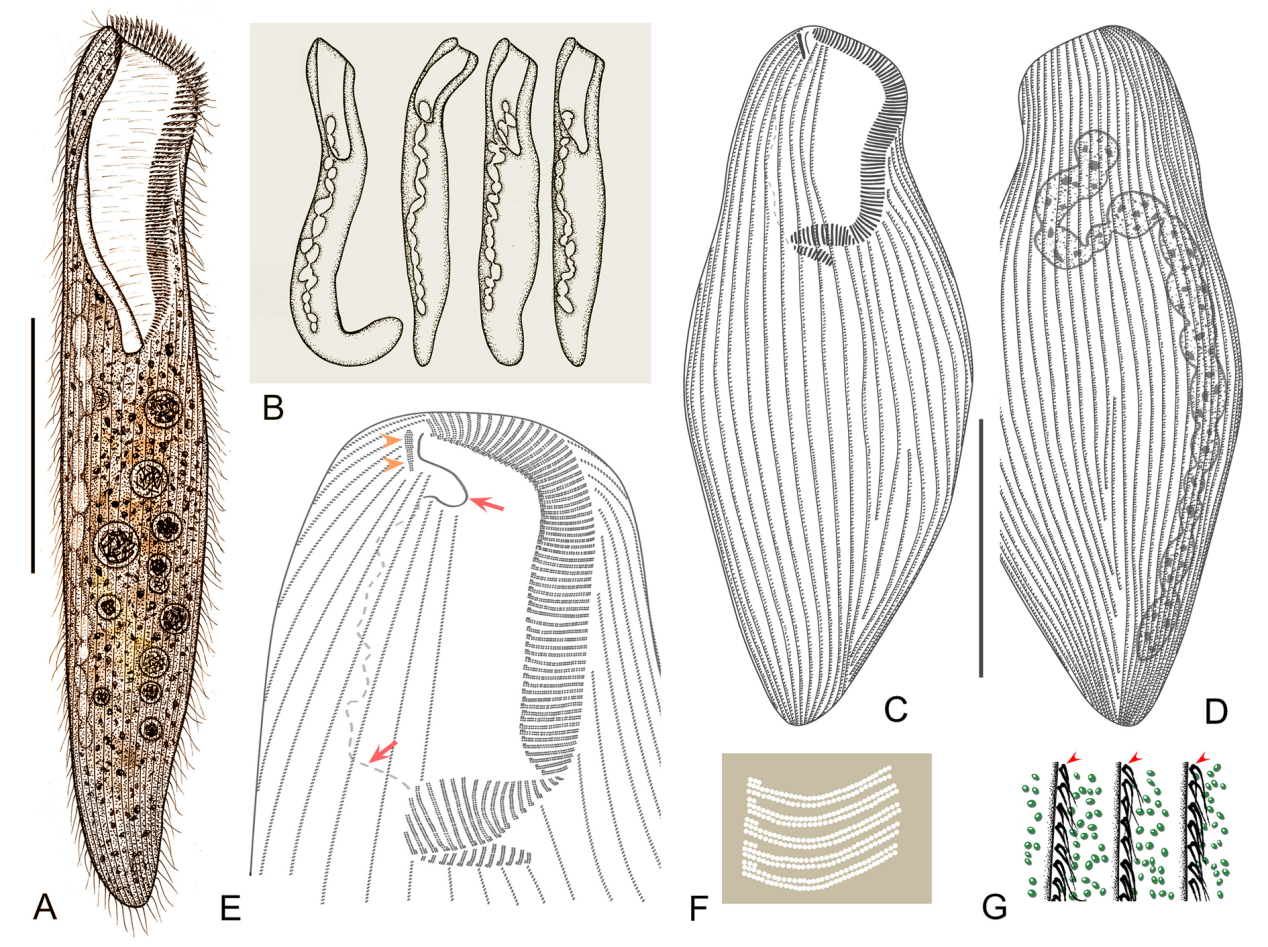
Schematic drawings of Chinese populations of *Condylostoma minutum* from life (**A**,** B**,** G**) and after protargol staining (**C**–**F**).** A** General view of a typical individual.** B** Different cell shapes and moniliform macronuclei.** C**, **D** Ventral (**C**) and dorsal (**D**) views of same specimen, showing the ciliature and macronucleus.** E** Detailed view of anterior portion of cell, arrows mark the paroral membrane, arrowheads mark the frontal membranelles.** F** Each adoral membranelle is composed of three rows (two long and one short) of basal bodies.** G** Showing cortical granules and that each basal body of dikinetids bears a cilium (arrowheads). Scale bars: 100 μm

**Fig. 13:**
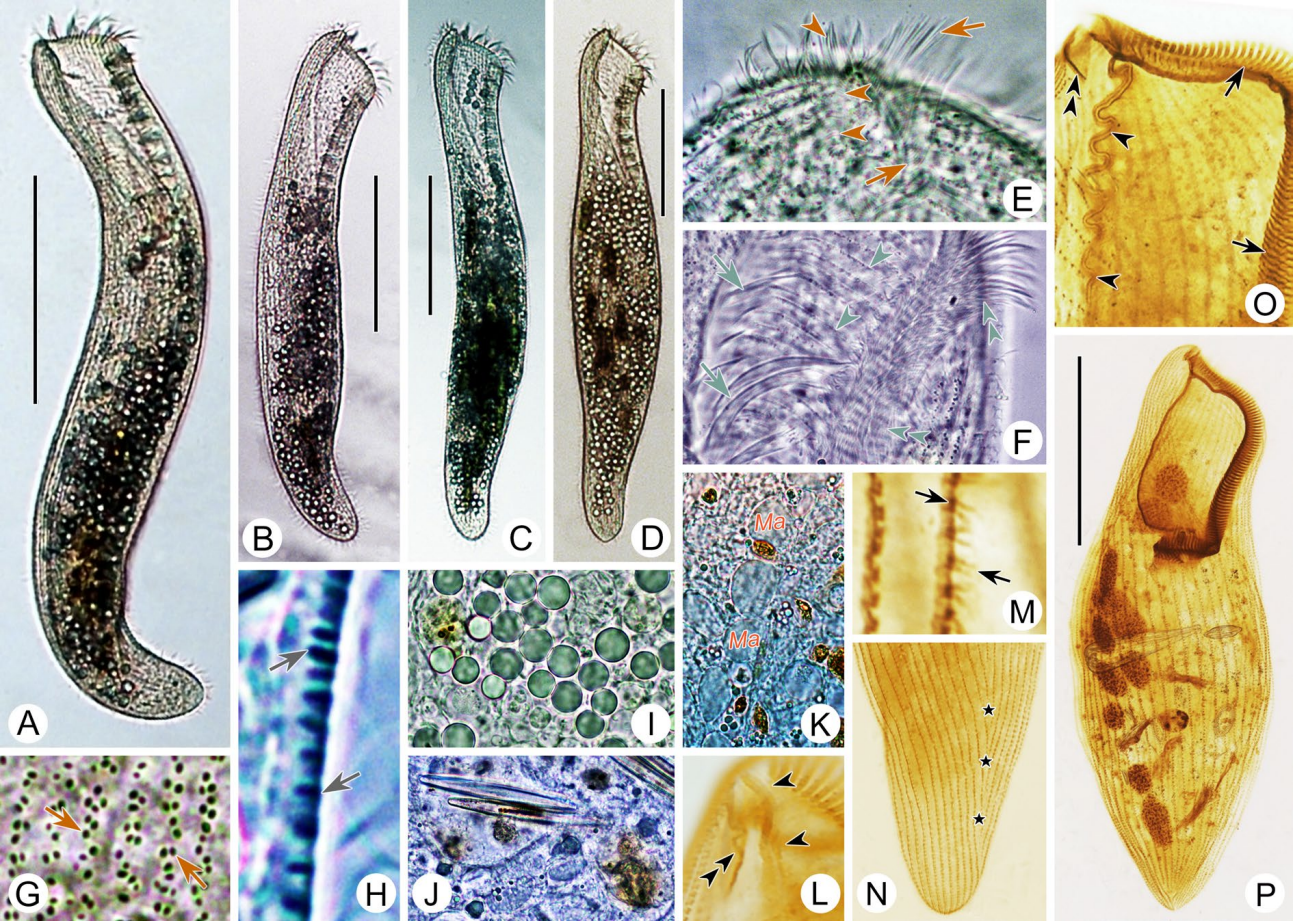
Photomicrographs of *Condylostoma minutum* pop.1 from life (**A**–**K**) and after protargol staining (**L**–**P**).** A**–**D** Various individuals showing different cell shapes. **E** Anterior portion of cell, arrows mark the paroral membrane, arrowheads show the frontal membranelles.** F** Buccal region, arrows mark the paroral membrane, arrowheads show the fiber-like stripes, double-arrowheads indicate the adoral zone of membranelles. **G** Cortical granules (arrows). **H** Tangential section of the cell, arrows mark the ellipsoidal cortical granules. **I**, **J** Cytoplasmic granules and food vacuoles containing algae. **K** Moniliform macronucleus. **L** Details of anterior portion, arrowheads show the slightly broaden distal end of paroral membrane, double-arrowhead indicates the frontal membranelles. **M** Each basal body of dikinetids bears a cilium (arrows). **N** Ventral view of posterior portion, asterisks mark the suture. **O** Ventral view of anterior portion, arrows mark the adoral zone of membranelles, arrowheads show the paroral membrane, double-arrowhead indicates the frontal membranelles. **P** Ventral side of a representative specimen, to show the ciliature and macronucleus. *Ma* macronucleus. Scale bars: 100 μm

***Description of population 1:*** Cell size 305–425 × 45–70 μm in vivo, on average about 365 × 60 μm; cell length to width ratio about 6:1. Cell elongate-ellipsoidal, slightly dorsoventrally flattened, anterior end slightly slanted to left, posterior end tapered (Figs. 12A, B, 13A–D). Macronucleus moniliform, composed of 12–18 nodules, located along right margin of cell; micronuclei not observed (Figs. 12A, B, 13K; Table 2). Pellicle flexible and thin, with ellipsoidal, gray-green cortical granules (about 1 μm in length), irregularly arranged with medium density between somatic kineties (Figs. 12G, 13G, H). Cytoplasm somewhat opaque, usually with numerous cytoplasmic granules and food vacuoles containing algae (Fig. 13I, J). Locomotion relatively slow, gliding on the bottom of Petri dishes or occasionally swimming.

Thirty-eight to 44 longitudinal somatic kineties, including 6–12 shortened rows both on ventral and dorsal sides (Figs. 12C, D, 13P). All somatic kineties composed of dikinetids; each basal body of dikinetids bears a cilium (Figs. 12C, D, G, 13M). Suture on ventral side of posterior portion of cell formed by shortened somatic kineties (Fig. 13N).

Buccal field conspicuous, 30–40% of cell length (Figs. 12A, B, 13A–D). About 82–108 adoral membranelles, each consisting of one short and two long rows of basal bodies (Figs. 12C, E, F, 13F, O). Fiber-like stripes regularly arranged on inner wall of oral cavity (Fig. 13F). Paroral membrane located on right side of buccal region, occasionally slightly broaden at distal end (Figs. 12E, 13E, F, L, O). Frontal membranelles located at distal end of paroral membrane, composed of a single stripe formed by aggregations of kinetosomes (Pattern II) (Figs. 12E, 13E, L, O).

***Description of population 2:*** Population 2 (Supplementary Fig. S1M–P) is generally consistent with population 1 in terms of cell length in vivo (270–435 μm), the ratio of buccal length to cell length (30–40%), the shape, color and arrangement of cortical granules, and the numbers of macronuclear nodules (11–22), somatic kineties (41–48) and adoral membranelles (85–108). However, population 2 has a wider cell (ratio of length to width 4:1 vs. 6:1) and inhabits a higher salinity habitat (22.0‰ vs. 10.4‰).

#### ***Condylostomidescoeruleus* Foissner, 2016**

***Voucher slides:*** Seven voucher slides with protargol-stained specimens were deposited in the Laboratory of Protozoology, Ocean University of China (OUC) with registration numbers: CY2020061501-01–07.

**Fig. 14:**
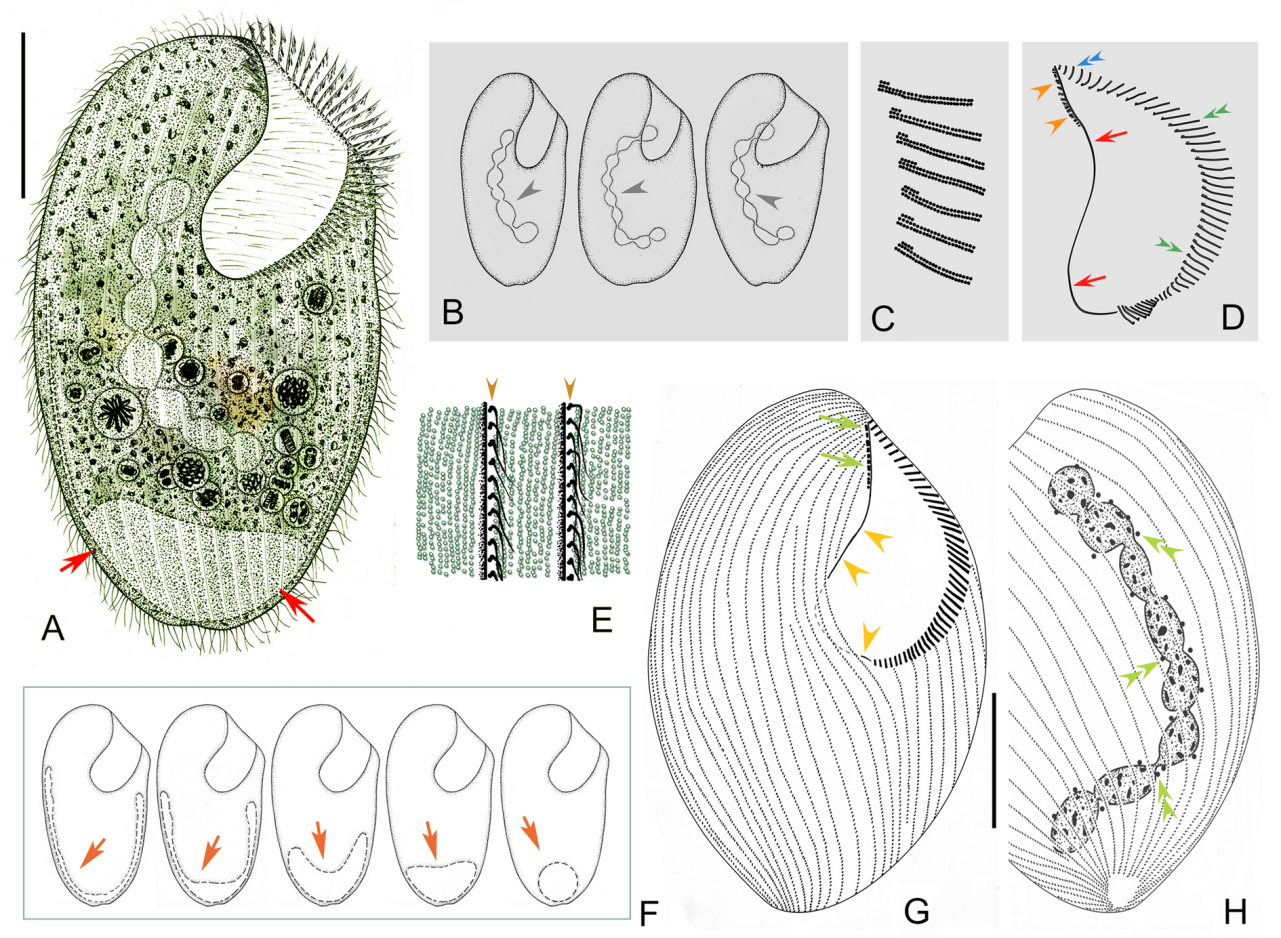
Schematic drawings of Chinese population of *Condylostomides coeruleus* from life (**A**,** B**,** E**, **F**) and after protargol staining (**C**,** D**, **G**,** H**).** A** General view of a typical individual, arrows mark the contractile vacuole. **B** Different cell shapes and moniliform macronuclei (arrowheads).** C** Most adoral membranelles are composed of three rows (two long and one short) of basal bodies. **D** Details of buccal region, arrows mark the paroral membrane, arrowheads show the frontal membranelles, green double-arrowheads indicate the adoral membranelles composed of one short and two long rows of basal bodies, blue double-arrowhead indicates the adoral membranelles composed of only two long rows of basal bodies. **E** Cortical granules regularly arranged in rows between adjacent somatic kineties, arrowheads show only one basal body of dikinetids bears a cilium. **F** The diastolic process of the contractile vacuole (arrows). **G**, **H** Ventral (**G**) and dorsal (**H**) views of same specimen, arrows mark the frontal membranelles, arrowheads show the paroral membrane, double-arrowheads indicate the micronuclei. Scale bars: 60 μm

**Fig. 15:**
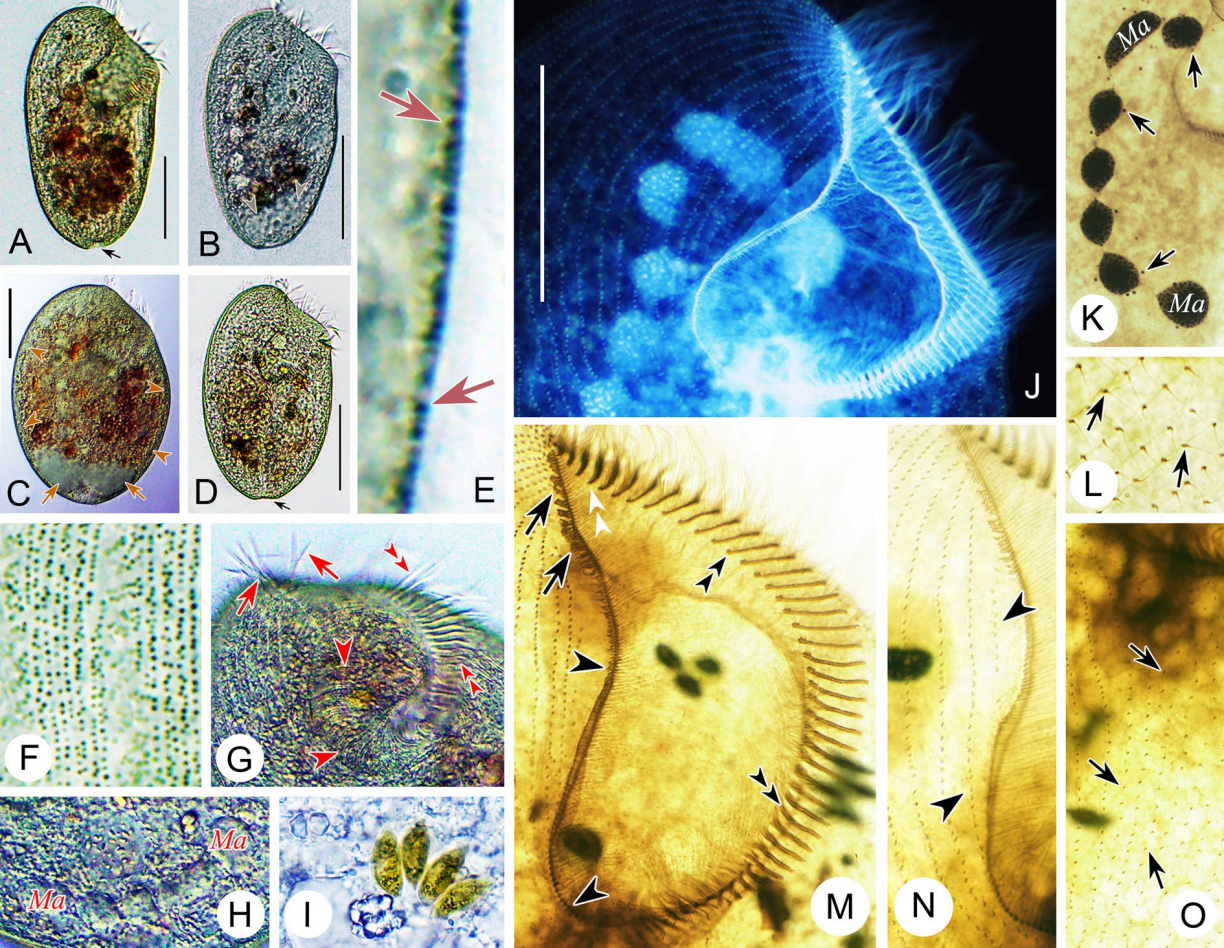
Photomicrographs of Chinese population of *Condylostomides coeruleus* from life (**A**–**I**) and after protargol staining (**J**–**O**).** A**, **B**, **D** Various individuals to show different cell shapes, arrows mark the cytopyges, arrowheads show the contractile vacuole. **C** A squashed cell, arrows mark the contractile vacuole, arrowheads show the collecting canals. **E** Tangential section of the cell, arrows mark the cortical granules. **F** Cortical granules regularly arranged in rows between adjacent somatic kineties. **G** Anterior portion of cell, arrows mark the frontal membranelles, arrowheads show the paroral membrane, double-arrowheads indicate the adoral zone of membranelles. **H** Moniliform macronucleus. **I** Food vacuole with algae. **J** Photomicrographs of ventral view modified with invertible function in Photoshop. **K** Nuclear apparatus, arrows mark the micronuclei. **L** Only one basal body of each dikinetid bears a cilium (arrows). **M** Ventral view of anterior portion, arrows mark the frontal membranelles, arrowheads show the paroral membrane, black double-arrowheads indicate the adoral membranelles composed of one short and two long rows of basal bodies, white double-arrowhead indicates the adoral membranelles composed of only two long rows of basal bodies. **N**, **O** Shortened somatic kineties originate the right of paroral membrane (arrowheads) or below buccal cavity (arrows). *Ma* macronucleus. Scale bars: 80 μm

***Description:*** Cell size 185–245 × 90–135 μm in vivo, on average about 215 × 115 μm; ratio of cell length to width about 2:1. Cell shape ellipsoidal, conspicuously dorso-ventrally flattened, anterior end obliquely truncated, posterior end slightly depressed due to cytopyge (Figs. 14A, B, 15A, B, D). Macronucleus moniliform with 6–10 nodules, located in mid-region of cell; micronuclei nearly spherical and numerous, 14–34, on average about 20, in number (N = 21), about 2 μm in diameter after protargol-staining, closely associated with macronuclear nodules (Figs. 14A, B, H, 15H, K; Table 2). Contractile vacuole in posterior region, varies in shape during diastolic process, with two collecting canals extending to mid-region of cell (Figs. 14A, F, 15C). Pellicle flexible and thin, with one type of broadly ellipsoidal to globular, blue-green cortical granules (about 0.5 μm in diameter), densely arranged in 7–9 rows between somatic kineties (Figs. 14E, 15E, F). Cell usually grayish-green when observed at low magnifications. Cytoplasm somewhat opaque, usually with numerous cytoplasmic granules and food vacuoles (Figs. 14A, 15A–D, I). Locomotion usually by gliding slowly over substratum, occasionally swimming while rotating about the main cell axis.

Somatic cilia about 10 μm long in vivo. Forty-two to 52 somatic kineties composed of densely spaced dikinetids, only one basal body of each dikinetid bears a cilium (Figs. 14E, G, H, 15J, L). About 4–8 shortened or fragmented somatic kineties, originating right of paroral membrane and below oral cavity (Figs. 14G, 15N, O).

Buccal field conspicuous, 40–50% of cell length, beginning at apical end and terminating in mid-cell region (Figs. 14A, B, 15A, B, D). Adoral zone of membranelles conspicuous, composed of 43–52 membranelles, most of which consist of one short and two long rows of basal bodies; several anterior adoral membranelles consist of only two long rows (Figs. 14C, D, 15G, M). Paroral membrane conspicuous and visible in vivo, located at right margin of buccal cavity, commences apically, and extends to proximal end of adoral zone (Figs. 14D, G, 15G, M). Frontal membranelles closely connected to right of distal end of paroral membrane, consisting of relatively indistinct multiple block-like structures formed by aggregations of kinetosomes (Pattern III) (Figs. 14D, G, 15M).

## Conclusions

Limited by a variety of factors, the correct species identification of individuals from the most species-rich heterotrich genus, *Condylostoma*, has long been a challenging task. This difficulty has, in turn, raised concerns regarding potential misidentifications of numerous gene sequences in publicly accessible databases, thus impeding our understanding of their evolutionary relationships. To address this issue, we conducted a comprehensive review of two genera, based on morphological redescriptions and phylogenetic analyses of four highly confusable *Condylostoma* species and one *Condylostomides* species isolated for the first time from the Asian continent. In this review, a total of 43 nominal species and about 130 populations were reassessed from the perspective of modern taxonomy, ultimately identifying 30 valid *Condylostoma* species and eight *Condylostomides* species. Furthermore, we updated the identification criteria for members of these two genera, examined their current documented biogeographical distributions, explored their phylogenetic relationships, and, interestingly, revealed the presence of cryptic species within *Condylostoma* for the first time. The present study provides a solid foundation upon which further future investigations into these and other related genera can be built.

### Supplementary Information

Below is the link to the electronic supplementary material.

Supplementary file1 (DOC 6157 KB)

## Data Availability

Eight newly obtained SSU rDNA sequences were deposited in the GenBank database with accession numbers OR553808–OR553815.
