# Supplementary material for: Exploring the biogeography, morphology, and phylogeny of the condylostomatid ciliates (Alveolata, Ciliophora, Heterotrichea), with establishment of four new Condylostoma species and a revision including redescriptions of five species found in China
Source: Mar Life Sci Technol. 2024 Jun 26;6(3):365–404. doi: 10.1007/s42995-024-00223-3 (PMC11358585; doi:10.1007/s42995-024-00223-3)
Supplement: Supplementary file 1 — Supplementary file1 (DOC 6157 KB) [file 42995_2024_223_MOESM1_ESM.doc]

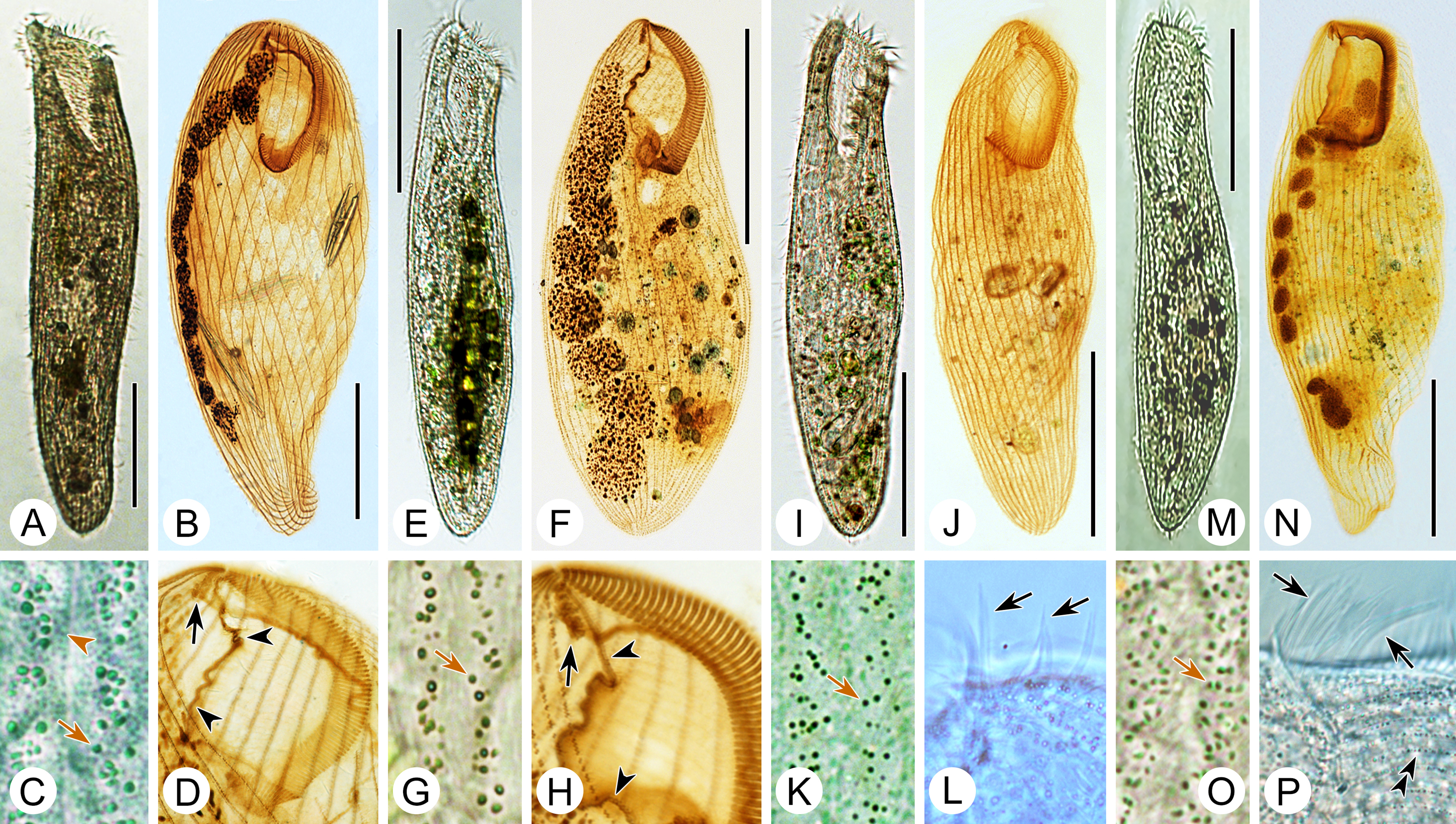


**Supplementary Fig. S1** Photomicrographs of *Condylostoma kris* pop.2 (**A**–**D**), *C. curvum* pop.2 (**E**–**H**), *C. curvum* pop.3 (**I**–**L**), and *C. minutum* pop.2 (**M**–**P**). **A**, **E**, **I**, **M** General views of typical individuals from life. **B**, **F**, **J**, **N** Ventral sides of representative specimens after protargol staining. **C**, **G**, **K**, **O** Details of cortex, arrows mark the large (>1 μm) and ellipsoidal cortical granules, arrowhead shows the small (<1 μm) and spherical cortical granules. **D**, **H**, **L**, **P** Anterior portion of cells, arrows mark the frontal membranelles, arrowheads show the paroral membrane, double-arrowhead indicates the fiber-like stripes. Scale bars: 100 μm.
